# Umbrella review and Delphi study on modifiable factors for dementia risk reduction

**DOI:** 10.1002/alz.13577

**Published:** 2023-12-30

**Authors:** Colin Rosenau, Sebastian Köhler, Lion M. Soons, Kaarin J. Anstey, Carol Brayne, Henry Brodaty, Knut Engedal, Francesca R. Farina, Mary Ganguli, Gill Livingston, Constantine G. Lyketsos, Francesca Mangialasche, Laura E. Middleton, Marcel G. M. Olde Rikkert, Ruth Peters, Perminder S. Sachdev, Nikolaos Scarmeas, Geir Salbæk, Martin P. J. van Boxtel, Kay Deckers

**Affiliations:** ^1^ Alzheimer Centrum Limburg Department of Psychiatry and Neuropsychology School for Mental Health and Neuroscience (MHeNs) Maastricht University Maastricht the Netherlands; ^2^ School of Psychology University of New South Wales Kensington New South Wales Australia; ^3^ Neuroscience Research Australia (NeuRA) Sydney New South Wales Australia; ^4^ UNSW Ageing Futures Institute Kensington New South Wales Australia; ^5^ Cambridge Public Health University of Cambridge Cambridge UK; ^6^ Centre for Healthy Brain Ageing (CHeBA) Discipline of Psychiatry and Mental Health School of Clinical Medicine University of New South Wales Sydney New South Wales Australia; ^7^ Norwegian National Centre for Ageing and Health Vestfold Hospital Trust Tønsberg Norway; ^8^ Feinberg School of Medicine Department of Medical Social Sciences Northwestern University Chicago Illinois USA; ^9^ Departments of Psychiatry Neurology and Epidemiology School of Medicine and School of Public Health University of Pittsburgh Pittsburgh Pennsylvania USA; ^10^ Division of Psychiatry University College London London UK; ^11^ Richman Family Precision Medicine Center of Excellence in Alzheimer's Disease Johns Hopkins Bayview Johns Hopkins Medicine Baltimore Maryland USA; ^12^ Division of Clinical Geriatrics Department of Neurobiology Care Sciences and Society Center for Alzheimer Research Karolinska Institutet Stockholm Sweden; ^13^ Theme Inflammation and Aging Medical Unit Aging Karolinska University Hospital Stockholm Sweden; ^14^ Department of Kinesiology and Health Sciences University of Waterloo Waterloo Ontario Canada; ^15^ Schlegel‐UW Research Institute for Aging Waterloo Ontario Canada; ^16^ Department of Geriatric Medicine Radboud University Medical Center Nijmegen the Netherlands; ^17^ Radboudumc Alzheimer Center Donders Center of Medical Neurosciences Nijmegen the Netherlands; ^18^ The George Institute for Global Health Newtown New South Wales Australia; ^19^ School of Biomedical Sciences University of New South Wales Kensington New South Wales Australia; ^20^ 1st Department of Neurology Aiginition Hospital National and Kapodistrian University of Athens Medical School Athens Greece; ^21^ Department of Neurology Columbia University New York New York USA; ^22^ Department of Geriatric Medicine Oslo University Hospital Oslo Norway; ^23^ Institute of Clinical Medicine University of Oslo Oslo Norway

**Keywords:** brain health, cognitive decline, cognitive impairment, Delphi study, dementia, etiological risk factors, hearing impairment, lifestyle, prevention, protective factors, risk prediction, risk reduction, sleep, social contact, umbrella review

## Abstract

A 2013 systematic review and Delphi consensus study identified 12 modifiable risk and protective factors for dementia, which were subsequently merged into the “LIfestyle for BRAin health” (LIBRA) score. We systematically evaluated whether LIBRA requires revision based on new evidence. To identify modifiable risk and protective factors suitable for dementia risk reduction, we combined an umbrella review of systematic reviews and meta‐analyses with a two‐round Delphi consensus study. The review of 608 unique primary studies and opinions of 18 experts prioritized six modifiable factors: hearing impairment, social contact, sleep, life course inequalities, atrial fibrillation, and psychological stress. Based on expert ranking, hearing impairment, social contact, and sleep were considered the most suitable candidates for inclusion in updated dementia risk scores. As such, the current study shows that dementia risk scores need systematic updates based on emerging evidence. Future studies will validate the updated LIBRA score in different cohorts.

**Highlights:**

- An umbrella review was combined with opinions of 18 dementia experts.
- Various candidate targets for dementia risk reduction were identified.
- Experts prioritized hearing impairment, social contact, and sleep.
- Re‐assessment of dementia risk scores is encouraged.
- Future work should evaluate the predictive validity of updated risk scores.

## 1 BACKGROUND

Considering the anticipated substantial increase in numbers of people with dementia, prevention and treatment of dementia have become global health priorities.^1^ An increasing number of modifiable risk and protective factors for dementia risk reduction has attracted much attention.^2^ According to The Lancet Commission on Dementia Prevention, Intervention, and Care, ≈ 40% of all dementia cases worldwide could theoretically be prevented or delayed by tackling 12 common modifiable risk and protective factors.^3^ The potential for prevention might be even higher (up to 56%) in low‐ and middle‐income countries and in minority groups within countries.^4^, ^5^

Several dementia risk scores have been developed to improve the identification of those at higher risk. The most used and validated risk scores include CAIDE (Cardiovascular Risk Factors, Aging, and Incidence of Dementia score), ANU‐ADRI (Australian National University Alzheimer's Disease Risk Index), and LIBRA (LIfestyle for BRAin health index).^6^ CAIDE originates from 2006 and is based on a 20‐year follow‐up of the CAIDE study.^7^ This score aims to predict late‐life risk of dementia based on a limited set of modifiable and non‐modifiable risk factors (e.g., blood pressure, age, sex, etc.) and has been validated for both cognitive decline^8^, ^9^ and dementia^7^ in multiple cohorts as well as a prevention trial outcome.^10^ ANU‐ADRI was developed in 2013 as a risk score incorporating both non‐modifiable and modifiable risk factors for Alzheimer's disease (AD) that could be readily assessed via self‐report.^11^ It has been used as a predictor for dementia^12^, ^13^ and cognitive decline^14^ in multiple cohorts as well as a surrogate outcome in clinical trials to assess intervention effectiveness.^15^, ^16^

The LIBRA index was also developed in 2013 based on the results of a systematic literature review and Delphi consensus study including eight dementia experts.^17^ The score is composed exclusively of modifiable risk (coronary heart disease, physical inactivity, chronic kidney disease, diabetes, cholesterol, smoking, midlife obesity, midlife hypertension, and depression) and protective factors (low‐to‐moderate alcohol consumption, healthy diet, and high cognitive activity) for cognitive decline and dementia that can be targeted by lifestyle interventions in primary care, thereby capturing lifestyle‐based risk reduction potential on an individual level.^18^ LIBRA has been well validated for dementia risk,^19^, ^20^, ^21^ cognitive decline,^22^ and structural markers of brain damage^23^ in numerous population‐based cohorts. In addition, it has also been used as an outcome measure in multidomain intervention studies.^24^, ^25^

Recently, the World Health Organization (WHO) guidelines on risk reduction of cognitive decline and dementia advocated the use of risk scores for the implementation of risk reduction guidelines.^6^ In the decade after establishment of CAIDE, ANU‐ADRI, and LIBRA, research has identified modifiable risk factors that might be additional targets for dementia risk reduction and hence should be included in contemporary risk scores. Identifying and prioritizing the modifiable risk factors for use in updated risk scores could improve dementia risk prediction and inform the development of more effective risk reduction strategies. Therefore, the aims of the current study are (1) to summarize the evidence on modifiable risk and protective factors for dementia and (2) to identify and prioritize candidate factors for updating dementia risk scores.

## 2 METHODS

### 2.1 Phase 1: umbrella review

#### 2.1.1 Data sources and search strategy

The search strategy was adapted from our previous study for the identification of modifiable dementia risk and protective factors^17^ and was composed of multiple text and Medical Subject Heading (MeSH) terms for exposure (e.g., “risk factor [ALL]”, “epidemiologic factor [MESH]”) and cognitive outcome (e.g., “dementia [ALL]”, “cognition disorders [MESH:noexp]”). The strategy was restricted to studies in humans written in Dutch or English. To ensure adequate coverage of the existing literature, we searched PubMed, Embase, Web of Science, and PsycINFO for the period from January 1, 2015 to May 23, 2021 to identify all systematic reviews (SR) and/or meta‐analyses (MA) that reported modifiable dementia risk and protective factors.^26^ See Appendix A in supporting information for the complete search strategies used in the different databases. The umbrella review was registered in PROSPERO (CRD42021266486) and conducted in line with the Preferred Reporting Items for Systematic Reviews and Meta‐Analyses (PRISMA) guidelines.

**RESEARCH IN CONTEXT**

1. **Systematic review**: The emergence of more recently described modifiable risk and protective factors for dementia highlights the need for continuous re‐assessment of existing dementia risk scores. The authors systematically reviewed the literature using PubMed, Embase, Web of Science, and PsycINFO, combined with dementia experts’ opinions.
2. **Interpretation**: Our findings identified new candidate risk factors, with the strongest support for hearing impairment, social contact, and sleep.
3. **Future directions**: Updating dementia risk scores may improve their predictive validity and allow for more extensive and tailored dementia risk reduction strategies.

#### 2.1.2 Study selection

We included SR/MA reporting studies that: (1) examined modifiable risk and protective factors as the exposures; (2) contained information on dementia, AD dementia, other dementia subtypes, mild cognitive impairment (MCI), cognitive impairment/decline, or change in cognitive performance as outcome; (3) used data from population‐based samples; (4) had prospective cohort study designs with ≥ 2 years follow‐up; (5) included ≥ 200 participants, who were (6) aged ≥ 18 years, and (7) reported either risk estimates for incident dementia, one of its subtypes, or MCI (hazard ratios, risk ratios, odds ratios) or regression coefficients (for continuous cognitive outcomes) with 95% confidence intervals (CIs) in text, tables, or forest plots. Regarding the latter criterion, evidence from risk estimates was considered primary outcomes in studies that reported both risk estimates and regression coefficients.

#### 2.1.3 Data extraction

After de‐duplication of the search hits, two investigators (C.R., L.S.) screened titles and abstracts for broad suitability.^27^, ^28^ Any disagreements were resolved by discussing with other investigators (K.D., S.K., M.v.B.). Subsequently, the following information was extracted from full texts using a predefined data‐extraction spreadsheet (see Appendix B in supporting information): (1) study title, (2) risk or protective factor(s) discussed, (3) exposed group, (4) reference group, (5) cohort, (6) cognitive outcome(s), (7) type of outcome measure(s), (8) risk estimator or regression coefficient with 95% CIs, (9) sample size, (10) follow‐up time, and (11) average baseline age of participants (or approximation). As much information as possible was extracted from the SR and/or MA. In cases that did not report certain information, this was retrieved directly from the included primary studies.

#### 2.1.4 Data synthesis

Based on the direction of the risk estimators or regression coefficients and the width of their 95% CIs, we decided whether an association was labeled a “risk association”, a “protective association”, or a “neutral association”. Consistency of the associations for every factor was calculated as the highest number of either “risk associations” or “protective associations”, divided by the total number of associations encountered for that factor. For feasibility, the number of associations assessed for every primary study was limited to one, unless multiple SR/MA discussed different risk factors that were investigated in the study. In cases in which different exposure levels of a particular factor were compared to the same reference, we selected the association with the most extreme exposure level compared to the reference as the basis of our decision, unless there was substantial evidence for a non‐linear relationship between exposure and outcome. Additionally, when multiple cognitive outcomes were assessed, we used the association with the most generalizable outcome in the order of (1) all‐cause dementia, (2) AD dementia, (3) other dementia subtypes, (4) MCI, (5) cognitive impairment/decline, or (6) change in cognitive performance based on neuropsychological testing. Whenever two studies reported the same study cohort, the association for the most generalizable cognitive outcome was included. If two papers were of the same study cohort and of the same outcome–exposure association, we chose the paper with the largest sample size or longest follow‐up period. Appendix C in supporting information gives a more detailed description of these data harmonization procedures.

#### 2.1.5 Quality assessment

Study quality of the included SR/MA was assessed using the 7/8‐item National Institutes of Health (NIH) Quality Assessment Tool of Systematic Reviews and Meta‐analyses. The quality assessment for all included studies can be found in Appendix D in supporting information.

### 2.2 Phase 2: Delphi consensus study—first round

As in our previous Delphi study, this study was conducted among dementia prevention experts.^17^ A total of 42 experts were invited by e‐mail (with one or two reminders in case of non‐response), all of whom were assistant, associate, or full professors and were considered experts in the field based on their track records. We actively invited experts from different regions across the world with different research and/or clinical backgrounds. The experts gave informed consent and completed an online questionnaire (May 2022–June 2022) through a unique hyperlink using Qualtrics XM (see Appendix E in supporting information). The participants were asked to indicate for every current LIBRA factor whether it should or should not be included in an updated version of this dementia risk score, with a free text field to support their choice in case of non‐inclusion. Subsequently, they were asked to name additional modifiable dementia risk and protective factors and rank them in subjective order of importance. The experts gave each of these newly named risk and protective factors a rank score, which allowed us to aggregate responses across experts to calculate a factor's total rank score (see Appendix F in supporting information). The researchers processed the responses using aggregated results to ensure blinding. This Delphi study was approved by the ethical committee of Maastricht University (FHML‐REC/2022/086).

### 2.3 Phase 3: synthesis of information

Next, a risk factor inventory was constructed, based on the highest‐ranking factors of both the umbrella review and the first Delphi round that were not previously included in the LIBRA index. The risk and protective factors encountered in the umbrella review were ranked based on their frequency in the literature and, subsequently, the consistency of their associations with cognitive outcomes, while the factors encountered in the first Delphi round were ranked according to the risk factor's total rank score (see Appendix F).

### 2.4 Phase 4: Delphi consensus study—second round

The second Delphi round (online, December 2022–January 2023, using Qualtrics XM, Appendix G in supporting information) focused on the risk and protective factors not yet included in LIBRA. The same panel of experts was presented with the aggregated results of the umbrella review and the first Delphi round. The list of factors was composed of the factors mentioned in the first Delphi round (suitable for individual‐level risk reduction), supplemented with the factors identified with our umbrella review that had a “consistency of association” of at least 50%. The experts were asked to weigh the candidate risk and protective factors for dementia risk reduction by freely distributing 100 points (more points = more important) across the inventory. The summation of these points across experts resulted in final scores for all of the different factors, which allowed us to create a definite curated list of the most important modifiable risk and protective factors. In addition, we asked experts’ opinions with regard to the operationalization of some specific factors.

## 3 RESULTS

### 3.1 Umbrella review

**FIGURE 1:**
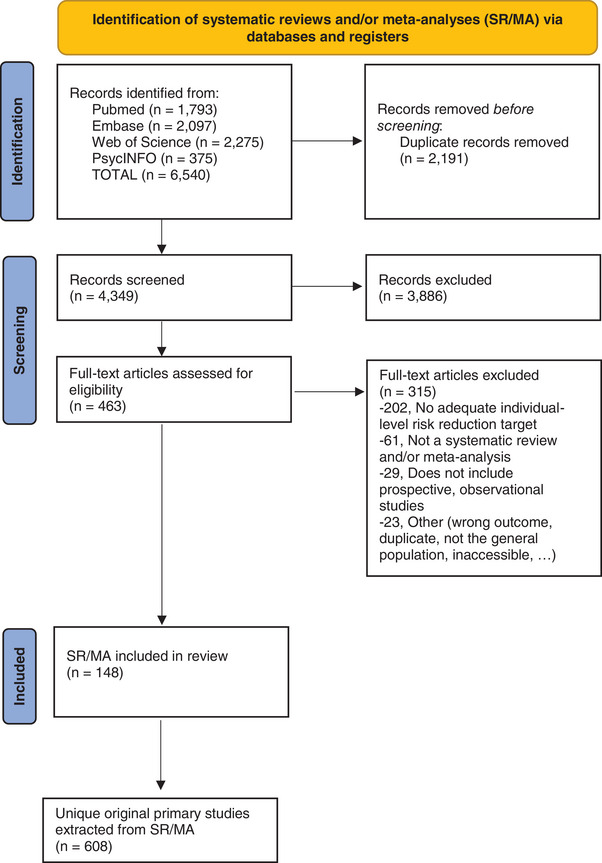
Flow diagram of the study selection process.

With the initial search, a total of 6540 abstracts were retrieved, yielding 4349 unique abstracts after de‐duplication. Of these abstracts, 463 were included for full‐text assessment. From these, 316 were excluded for various reasons (see Figure 1), resulting in a total inclusion of 148 SR/MA in our umbrella review. After scrutinizing these SR/MA, a total of 608 unique primary studies included in those SR/MA were considered eligible for our analysis. For all the different risk and protective factors encountered in these studies, we determined the frequency and consistency of association with cognitive outcomes. For 9 of the 12 factors currently included in the LIBRA index, we found that more than half of the studies included in the SR/MA reported a significant association with cognitive outcomes. For the three other factors (low/moderate alcohol consumption, midlife obesity, and smoking), less than half of the studies included in the SR/MA reported a significant association between exposure and cognitive outcome (Table 1). Besides the existing LIBRA factors, there were many other candidate factors. The most well studied are reported in Table 2 and the overview of the encountered factors together with the primary literature in which they were investigated can be found in Appendix H in supporting information.

**TABLE 1:** Frequency and consistency of the encountered factors currently included in the LIBRA index based on the umbrella review of 148 systematic literature reviews and meta‐analyses.

LIBRA factor	Number of studies	Higher risk/decline	No association	Lower risk/decline	Consistency of association
Diabetes	61	31	30	0	51%
Depression	48	36	12	0	75%
Midlife hypertension	28	17	8	2	63%
High leisure‐time physical activity^a^	22	0	7	15	68%
High alcohol consumption^b^	22	3	15	4	18%
Chronic kidney disease	20	14	6	0	70%
High cognitive activity	20	0	7	13	68%
Healthy diet/Mediterranean diet	18	0	8	10	56%
Coronary heart disease	15	10	5	0	67%
Smoking	14	6	8	0	43%
Midlife obesity	9	2	7	0	22%
High midlife cholesterol	8	5	3	0	63%

*Notes*: These factors were ordered based on their frequency and, subsequently, their consistency of association in the primary literature. For each factor, this was calculated as the highest number of studies that found a significant association in one direction (higher or lower risk) divided by the total amount of studies on that factor.

Abbreviation: LIBRA, LIfestyle for BRAin health.

^a^ Inverse of the operationalization in the original LIBRA index ( = physical inactivity).

^b^ Inverse of the operationalization in the original LIBRA index ( = low/moderate alcohol intake).

**TABLE 2:** Overview of best‐documented (newly identified) modifiable risk and protective factors (not previously included in the LIBRA index) based on the umbrella review of 148 systematic literature reviews and meta‐analyses.

Non‐LIBRA factor	Number of studies	Higher risk/decline	No association	Lower risk/decline	Consistency of association
Low social engagement	28	15	13	0	54%
T2PD or impaired FBG	19	4	15	0	21%
Hearing impairment	18	14	4	0	78%
Long sleep duration	16	7	9	0	44%
Short sleep duration	16	5	11	0	31%
Vision impairment	15	10	5	0	67%
Atrial fibrillation	14	11	3	0	79%
Anxiety	14	10	4	0	71%
Low social network size	11	2	9	0	18%
Loneliness	10	7	3	0	70%
Tooth loss	10	7	3	0	70%
Poor sleep quality	10	5	5	0	50%
Orthostatic hypotension	10	4	6	0	40%
Living alone	10	3	6	1	30%
Olfactory impairment	9	9	0	0	100%
Insomnia	9	4	4	1	44%
Metabolic syndrome	8	3	5	0	38%
Daytime sleepiness	5	5	0	0	100%
High religious involvement	5	0	2	3	60%
Heart failure	5	2	3	0	40%
Sleep‐disordered breathing	5	2	3	0	40%
Low emotional support	5	0	5	0	0%
Any heart disease	4	1	3	0	25%
Periodontal disease	4	1	3	0	25%
High psychological stress	3	3	0	0	100%
Social difficulties	3	3	0	0	100%
Low serum folate	3	1	2	0	33%
Pesticide exposure	3	1	2	0	33%

*Notes*: These factors were ordered based on their frequency and, subsequently, their consistency of association in the primary literature. For each factor, this was calculated as the highest number of studies that found a significant association in one direction (higher or lower risk) divided by the total amount of studies on that factor. Factors encountered with a frequency ≤ 2 or that were too closely related to existing LIBRA factors, were omitted from this table.

Abbreviations: FBG, fasting blood glucose; LIBRA, LIfestyle for BRAin health; T2PD, type 2 prediabetes.

### 3.2 Delphi consensus study—first round

Of the invited 42 experts, 18 agreed to participate. The majority of experts agreed all 12 factors included in the current LIBRA index should continue to be included. Table 3 shows the different factors with the percentage of experts that supported the inclusion of that factor in an updated LIBRA index. The lowest consensus was for the factors chronic kidney disease, coronary heart disease, and high cholesterol, for which consensus varied between 11 (61%) and 13 (72%) of the 18 experts. For the remaining 9 factors, 16 or more experts (≥ 89%) agreed on their inclusion. The main reasons for non‐inclusion according to the experts were life course differences in effects, inconsistent evidence, and limited evidence for modifiability. In addition, experts named 27 new modifiable risk and protective factors for dementia risk reduction. Table 4 shows the answers with the highest rank scores. A complete overview of the experts’ answers can be found in Appendix I in supporting information. Some of the answers provided were omitted from the second Delphi round as they did not align with the rationale behind the LIBRA index, that is, dementia risk reduction on an individual level through self‐management or primary care.

**TABLE 3:** Evaluation of the current LIBRA factors by the Delphi experts (*N* = 18) for inclusion in an updated index.

LIBRA factor	Supports inclusion	Does not support inclusion	Percentage supporting inclusion
Diabetes	18	0	100%
Physical inactivity	18	0	100%
Smoking	18	0	100%
Depression	17	1	94%
Midlife hypertension	17	1	94%
Midlife obesity	17	1	94%
High cognitive activity	16	2	89%
Healthy diet/Mediterranean diet	16	2	89%
Low/moderate alcohol intake	16	2	89%
Cholesterol	13	5	72%
Coronary heart disease	13	5	72%
Chronic kidney disease	11	7	61%

*Notes*: The experts were asked for each of the existing LIBRA factors to indicate whether that particular factor should be included or excluded in an updated version of the LIBRA index. The table provides an overview of these factors, ordered based on the number of experts favoring inclusion.

Abbreviation: LIBRA, LIfestyle for BRAin health.

**TABLE 4:** Ranking of (newly identified) modifiable risk and protective factors (not previously included in the LIBRA index) according to the experts (*N* = 18) in the first Delphi round.

Modifiable risk/protective factor	Frequency^a^	Ranks^b^	Rank score^c^	Final rank^d^
Hearing impairment	11	1,1,1,2,2,3,3,3,4,4,8	761	1
Social isolation/loneliness	13	1,2,2,2,3,3,3,3,3,4,4,5,7	723	2
Traumatic brain injury^e^	11	1,1,2,2,3,3,3,4,4,5,7	704	3
Education^e^	8	1,1,2,2,2,3,4,5	592	4
Sleep	8	1,1,1,2,2,4,5,6	572	5
Stroke/cerebrovascular disease^e^	5	1,1,1,5,8	345	6
Air pollution^e^	7	1,3,4,5,5,6,7	326	7
Psychological stress	3	2,3,8	154	8
Visual impairment	4	3,4,5,12	149	9
Life course inequalities	1	1	100	10
Atrial fibrillation	3	4,6,8	83	11

*Notes*: The experts were asked to list new modifiable risk and protective factors (not previously included in the LIBRA index) in order of subjective importance. The table provides an overview of these factors, ordered based on the factors’ rank scores.

Abbreviation: LIBRA, LIfestyle for BRAin health.

^a^ Frequency = The frequency that a particular factor was named.

^b^ Ranks = The ranks that were given to a particular factor.

^c^ Rank score = The score of a particular factor based on the ranks given (see Appendix F in supporting information for full calculations).

^d^ Final rank = The final rank of a particular factor based on its rank score.

^e^ In alignment with the development of the original LIBRA index, we do not consider these factors to be readily modifiable at an individual level (which does not mean that they are not modifiable at the population level). Because the LIBRA index is a tool to identify an individual's room for improvement in lifestyle, we omitted these factors from further analysis in this study.

### 3.3 Delphi consensus study—second round

In the second round, 17 of the 18 experts of the first round participated. They individually assigned a total of 100 points to the most important modifiable risk and protective factors identified in the umbrella review and first Delphi round. Table 5 shows the average number of points, the range, and the frequency of point allocation to each factor. Most points were allocated to (1) hearing impairment, (2) social contact, (3) sleep, (4) atrial fibrillation, (5) life course inequalities, and (6) psychological stress. The other factors identified in the umbrella review and first Delphi round received considerably fewer points and were therefore not considered for further inclusion in LIBRA. For the factors social contact and sleep, the experts were additionally asked to indicate which operationalization encountered in the literature would be the most suitable for modification. Social engagement (i.e., participation in activities with a social component such as volunteer work or organized group activities) and sleep‐disordered breathing were indicated as the most suitable operationalizations. However, the difference among the most suitable operationalizations for sleep were minimal (see Appendix J in supporting information).

**TABLE 5:** Ranking of emerging modifiable risk and protective factors (not previously included in the LIBRA index) according to the experts (*N* = 17) in the second Delphi round.

Modifiable risk/protective factor	Allocated points (mean)^a^	Standard deviation^b^	Frequency of point allocation^c^	Allocation range^d^
Hearing impairment	17.94	13.29	17	5–65
Life course inequalities	15.00	20.91	14	3–90
Social contact	13.82	6.54	17	5–30
Atrial fibrillation	12.94	8.75	16	5–40
Sleep	12.24	8.88	15	3–30
Psychological stress	12.15	7.15	16	3–30
Visual impairment	6.47	6.13	11	5–20
Anxiety	5.50	5.61	12	2.5–20
Tooth loss	2.94	3.46	8	5–10
Olfactory impairment	1.00	1.91	4	2–3

*Note*s: The experts had to allocate 100 points over the most important factors identified in the umbrella review and the first Delphi round. The table provides an overview of these factors, ordered based on the allocated points to each factor.

Abbreviation: LIBRA, LIfestyle for BRAin health.

^a^ Allocated points (mean) = The mean number of points allocated to each factor.

^b^ Standard deviation = The standard deviation of the mean number of points allocated to each factor.

^c^ Frequency of point allocation = The number of times that points were allocated to each factor.

^d^ Allocation range = The range of the points allocated to each factor.

### 3.4 Summary of the most important candidate risk and protective factors

#### 3.4.1 Hearing impairment

In the umbrella review, one SR^29^ and four MA^30^, ^31^, ^32^, ^33^ focused on hearing impairment and included a total of 18 unique prospective cohort studies.^34^, ^35^, ^36^, ^37^, ^38^, ^39^, ^40^, ^41^, ^42^, ^43^, ^44^, ^45^, ^46^, ^47^, ^48^, ^49^, ^50^, ^51^ Fourteen (78%) of these studies found a significantly increased risk of dementia or cognitive decline.^34^, ^36^, ^37^, ^38^, ^39^, ^40^, ^41^, ^42^, ^43^, ^44^, ^45^, ^48^, ^50^, ^51^ The two meta‐analyses that used dementia as the outcome reported combined effect sizes of 1.49 (95% CI 1.30–1.67) and 1.28 (95% CI 1.02–1.59).^30^, ^31^ Even higher combined effect sizes of 2.82 (95% CI 1.47–5.42) and 3.21 (95% CI 1.49–8.69) were calculated in two MA using cognitive impairment or AD as outcomes.^32^, ^33^ The latter study reported a higher effect estimate for severely versus moderately impaired central auditory processing, suggesting a potential dose–response effect.^33^

Recent cohort studies found that hearing aid use was associated with reduced risk of (progression to) dementia among hearing‐impaired adults.^52^, ^53^, ^54^ The first randomized controlled trial (RCT) on auditory rehabilitation, the Aging and Cognitive Health Evaluation in Elders (ACHIEVE) trial, showed a decrease of 48% in cognitive decline over 3 years in individuals with several risk factors for cognitive decline. However, this did not occur in a cohort of healthier individuals.^55^ Nevertheless, the experts considered hearing impairment the most important factor in both the first and second Delphi round (conducted before results of the ACHIEVE trial were published).

There are some mechanisms that could explain the association between hearing loss and dementia. One of the most well‐described theories is the information‐degradation hypothesis, suggesting that hearing impairment could affect cognitive performance by shifting cognitive resources from higher‐level cognitive processes toward sensory perception as a compensation mechanism. Alternatively, the relationship between hearing impairment and cognitive decline or dementia could be mediated by depression or social isolation. Finally, it has not been excluded that hearing impairment and dementia could have a common cause such as overall neural degeneration or systemic vascular disease, referred to as the common cause hypothesis.^56^, ^57^

#### 3.4.2 Social contact

Different operationalizations of social contact were encountered in this umbrella review. Some SR/MA focused on specific operationalizations of social contact such as loneliness^58^, ^59^ or living alone,^60^ while others investigated multiple operationalizations of social contact.^61^, ^62^ Functional operationalizations (“loneliness” and “social engagement”) of social contact were more consistently significantly associated with risk of dementia or cognitive decline compared to structural operationalizations (“social network size” and “living alone”) according to this umbrella review. One meta‐analysis including data from eight different prospective cohort studies found a relative risk of 1.26 (95% CI 1.14–1.40) for dementia or AD in lonely individuals.^59^ The meta‐analysis by Kuiper et al., found that the operationalizations “social participation”, “frequency of social contact”, and “loneliness” were associated with the risk of incident dementia, while this was not the case for “social network size”.^61^ A recent UK Biobank study reported that social isolation was significantly associated with a 26% increased risk of dementia, independent of loneliness and depression. Additionally, 75% of the dementia risk related to loneliness was mediated by depression.^63^

Despite the potentially overlapping nature of social and cognitively stimulating activities, the study by Duffner et al. reported independent effects for engagement in cognitive and social leisure activities.^64^ These results support the notion that social contact could be a suitable independent factor for dementia risk reduction, although more conclusive evidence is needed to determine which operationalization of social contact would be best targeted. As it appears difficult to address the effect of social contact in a controlled and systematic fashion, it is not possible to draw strong conclusions as to whether intervening on social contact will result in decreased dementia risk.^65^ The experts allocated the second and third highest number of points to social contact in the first and second Delphi study round, respectively. When we asked the experts to choose the most suitable operationalizations for intervention from a predefined list of operationalizations that we encountered in the literature, they also considered the earlier mentioned functional operationalizations to be more important than other operationalizations (see Appendix J). The experts noted the overlapping nature of the different operationalizations and mentioned the need for high‐quality studies with a long follow‐up time to minimize the risk of reverse causation.

Social contact, a form of environmental enrichment, might influence cognitive function through multiple mechanisms. This includes epigenetic regulation of brain‐derived neurotropic factor, which influences synaptic plasticity, neural repair, and neurogenesis.^66^ Alternatively, social contact could also increase brain connectivity and cognitive reserve, thereby improving brain resilience.^67^ Finally, social isolation and loneliness have been postulated to increase dementia risk through systemic inflammation.^68^

#### 3.4.3 Sleep

As with social contact, many different operationalizations of sleep were used in the literature. The majority of the SR/MA focused on sleep duration,^69^, ^70^, ^71^, ^72^, ^73^, ^74^ while others focused on sleep‐disordered breathing,^75^, ^76^, ^77^ insomnia,^78^, ^79^ or investigated multiple operationalizations of sleep.^80^, ^81^ The only operationalization of sleep that was found to be consistently associated with an increased risk of dementia or cognitive decline in the umbrella review was “excessive daytime sleepiness”. For all the other operationalizations of sleep, like “sleep‐disordered breathing”, “long sleep duration”, “short sleep duration”, “insomnia”, or “poor sleep quality”, only half or less of the primary studies found a significant association with cognitive outcomes in later life (Table 2 , Appendix H). The most recent meta‐analysis encountered in our umbrella review which analyzed multiple operationalizations of sleep reported effect sizes of 1.17 (95% CI 0.95–1.43), 1.18 (1.02–1.36), and 1.19 (1.11–1.29) for insomnia, sleep‐disordered breathing, and any sleep disturbance, respectively.^81^ However, there was considerable heterogeneity in how these operationalizations were made and measured in the different primary studies.

In both the first and second Delphi study rounds, sleep was allocated the fifth highest number of points. In contrast to social contact, for which the experts had a clear preference for certain operationalizations over others, there were no operationalizations of sleep that clearly stood out among the rest. From all the operationalizations encountered in our umbrella review, “sleep‐disordered breathing”, “short sleep duration”, “insomnia”, and “general poor sleep quality” were allocated the highest number of points, with minimal differences between them (see Appendix J). Experts expressed their concerns with regard to the overlapping nature of the operationalizations and the risk of reverse causality. Considering the mixed results from both our umbrella review and our Delphi consensus study, multiple operationalizations of sleep could potentially be used as entry points for dementia risk reduction. Whether disturbed sleep is a cause or an effect/symptom of dementia remains to be further elucidated. As such, well‐designed prospective cohort studies with clear definitions for the different operationalizations of sleep and long follow‐up times are warranted to better understand the association between (the different aspects/operationalizations of) sleep and dementia.

#### 3.4.4 Atrial fibrillation

In 11^82^, ^83^, ^84^, ^85^, ^86^, ^87^, ^88^, ^89^, ^90^, ^91^, ^92^ out of the 14^82^, ^83^, ^84^, ^85^, ^86^, ^87^, ^88^, ^89^, ^90^, ^91^, ^92^, ^93^, ^94^, ^95^ (79%) unique primary studies included in four different MA,^96^, ^97^, ^98^, ^99^ atrial fibrillation was significantly associated with worse cognitive outcome in later life. The four MA included in our umbrella review reported effect sizes of 1.39 (95% CI 1.25–1.53), 1.30 (95% CI 1.01–1.59), 1.34 (95% CI 1.24–1.44), and 1.36 (95% CI 1.23–1.51) on risk for dementia, AD, or cognitive decline. Only three experts mentioned atrial fibrillation in the first Delphi round and all gave it rather low priority. Hence, it was not included in the top 10 factors in the first Delphi round. In the second Delphi round, however, atrial fibrillation was allocated the fourth highest number of points. The association between atrial fibrillation and dementia risk is most likely multifactorial and includes mechanisms such as cerebral hypoperfusion, resulting in unfavorable “downstream” mechanisms, and a higher thrombotic state that could lead to silent micro‐infarctions.^100^ A recent large study including 18,813 primary care patients with atrial fibrillation found that use of oral coagulants was associated with a hazard ratio of 0.59 (95% CI 0.28–0.74) for dementia.^101^ Additionally, another recent study found that a healthier lifestyle in patients with new‐onset atrial fibrillation reduced risk for dementia, thereby advocating for the promotion of a healthy lifestyle in atrial fibrillation patient care.^102^

#### 3.4.5 Life course inequalities

Life course inequalities are not easily modifiable at the individual level and, therefore, do not align with the purpose of the LIBRA index. Accordingly, this factor was a priori excluded from our umbrella review. In the first Delphi round, one participant mentioned life course inequalities as a new modifiable factor, while 15 experts allocated points to this factor in the second Delphi round. As such, it ended as the second most important factor in our Delphi consensus study. A recent meta‐analysis of 39 prospective studies by Wang et al. found that individuals with low socioeconomic position have a 40% (95% CI 1.12–1.74) higher risk for all‐cause dementia compared to individuals with high socioeconomic position.^103^ In addition, a study by Klee et al. found that area‐level socioeconomic deprivation (air pollution, green spaces, etc.) was predictive of dementia, even after adjustment for individual‐level socioeconomic deprivation (e.g., household income and housing type).^104^ Mediation analysis within the English Longitudinal Study of Ageing also showed that the dementia risk (based on an algorithm combining physician diagnosis, self‐report, and informant report) difference between the highest and lowest wealth tertile was 52% mediated by differences in LIBRA scores, highlighting the importance of socioeconomic position in dementia risk reduction.^20^ As such, reducing social inequalities by targeting factors on both the individual and area level could play an important part in public health strategies addressing brain health.

#### 3.4.6 Psychological stress

We only identified one suitable SR^105^ on psychological stress, which included three prospective cohort studies that all observed a significant association between psychological stress and increased risk of dementia or cognitive decline.^106^, ^107^, ^108^ Psychological stress was mentioned by three experts, with relatively high priority. As such, psychological stress did reach the factor top 10 in the first Delphi round. In the second Delphi round, the experts allocated the sixth highest number of points to this factor. It has been argued that high psychological stress might influence dementia risk by dysregulation of the hypothalamic–pituitary–adrenal axis, thereby accelerating AD pathogenesis.^109^

## 4 DISCUSSION

Research on prevention of dementia has made major strides in the past 10 years. The present study adds knowledge by identifying and cross‐validating modifiable risk factors by combining quantitative information from an extensive umbrella review with quantitative and qualitative information from a Delphi consensus study including 18 internationally renowned dementia experts. After triangulation, strongest support was found for hearing impairment, social contact, and sleep within the scope of dementia risk reduction on an individual level. These factors could be added to current dementia risk scores (like LIBRA) to inform and advise people on their potential for improvement.

### 4.1 Re‐evaluation of established risk and protective factors

Most of the experts supported the inclusion of the original factors within an updated version of LIBRA. However, for 3 of the 12 factors (low/moderate alcohol intake, smoking, and midlife obesity), we found that less than half of the studies reported a significant association between the factor and cognitive outcome. For low/moderate alcohol intake, this could be due to considerable differences in cut‐off values for defining exposure categories (times/week, drinks/day, g/day, etc.) among the included studies, therefore increasing methodological heterogeneity and hindering direct comparison. In addition, because we only assessed exposure levels with the largest contrast (abstinence vs. highest drinking category reported), we might have missed differences between the exposed and reference group due to the poor general health of abstainers or misclassification bias.^110^, ^111^ Despite the seemingly limited consistency between alcohol consumption and cognitive outcome in our umbrella review, the most recent encountered MA reports a significantly increased risk for dementia among excessive drinkers.^112^ Additionally, high alcohol consumption could lead to other comorbidities such as hypertension, thereby increasing dementia risk even further.^113^ Considering the methodological challenges that might have affected our results, together with the strong evidence from both the MA as well as experts’ opinions, tackling high alcohol consumption remains a relevant factor for dementia risk reduction strategies. This is particularly important in light of increasing alcohol consumption.^114^

For the factors smoking and midlife obesity, the number of included prospective cohort studies was relatively low (*N* = 14 and *N* = 9), with six (43%) and two (22%) unique primary studies reporting a significantly increased risk for dementia or cognitive decline upon exposure, respectively. While these simple consistency percentages do not take effect size and sample size of the primary studies into account, the most recent MA encountered in our review reported effect sizes of 1.52 (95% CI 1.19–1.93) and 1.31 (95% CI 1.02–1.68) for smoking and midlife obesity, respectively.^115^, ^116^ As such, the evidence from both the MA and the experts indicate that smoking and midlife obesity remain relevant targets for reducing dementia risk.

### 4.2 Identification and operationalization of candidate risk factors

#### 4.2.1 Modifiable factors suitable for dementia risk reduction on an individual level

Hearing impairment is rather unidimensional, can be objectively assessed with clearly defined cut‐off values, and hence can be conveniently used to identify individuals at elevated risk for dementia. Additionally, both primary and secondary prevention of hearing impairment can be readily achieved by reducing exposure to excessive noise and, where unavoidable, promoting the use of hearing protection and hearing aids. The latter has been found to be associated with less cognitive decline compared to individuals with hearing impairment who do not use hearing aids.^54^ In contrast to hearing impairment, sleep and social contact are multidimensional.

Based on the current umbrella review and Delphi study, different aspects of social contact and sleep appear to be suitable targets for dementia risk reduction. It appears that functional aspects of social contact (i.e., loneliness, social engagement) are more consistently associated with cognitive outcome than structural aspects (i.e., living alone, social network size), and they might, therefore, be more suitable targets for dementia risk reduction. This factor could also be readily and inexpensively implemented in daily practice by stimulating individuals to participate in activities with a social component, such as attending cultural events, volunteering, or becoming member of an association or club. Additionally, this could have positive impact on other interrelated dementia risk factors, such as depression.^117^

In contrast to social contact, results for sleep were ambiguous, as no operationalization was clearly preferred over others by experts. Within the context of identifying and informing individuals at risk for this factor, it might, therefore, be more suitable to approach sleep from a more holistic perspective instead of focusing on one specific operationalization. This can, for example, be done with broad instruments, such as the Pittsburg Sleep Quality Index, which captures several sleep‐related operationalizations that we encountered in our umbrella review.^118^ Although promoting sleep hygiene could be an inexpensive strategy that can be readily incorporated in daily practice, it remains to be investigated which operationalization of sleep is most suitable for effective intervention.

#### 4.2.2 Modifiable factors less suitable for dementia risk reduction on an individual level

Life course inequalities, atrial fibrillation, and psychological stress are considered less suitable for individual‐level dementia risk reduction. Life course inequalities were considered outside of the scope of LIBRA, considering that they are not easily modifiable at the individual level and more likely an outcome of exposure to both up‐ and downstream determinants of health. Thus, they could be a suitable factor for population‐wide risk reduction strategies.

In contrast, atrial fibrillation could be considered a suitable risk factor for individual‐level dementia risk reduction. However, based on a relative risk of 1.39^97^ and an estimated prevalence of 1.77% among western Europeans between 60 and 65 years old,^119^ the population attributable fraction of atrial fibrillation (approximately 0.7%) is substantially lower than the other factors previously included in dementia risk scores.^3^ As such, the added value in updated risk scores would be rather limited.

Given the current evidence, the risk factor psychological stress might also be less suitable for inclusion in updated risk scores for multiple reasons. First, the most recent meta‐analysis on high psychological stress and risk for dementia (identified with a separate search after our umbrella review) based itself on only two studies for either high perceived stress or stressful life events.^120^ Thus, the evidence for stress as a risk factor is considered too low compared to other established factors. Second, psychological stress is an ill‐defined concept, and therefore difficult to measure, with potential distinctions among chronic psychological stress, acute psychological stress, stressful life events, and gray areas between.^121^ Third, psychological stress is highly correlated with depression, which itself is considered a well‐validated risk factor for dementia.^122^ As such, more long‐term cohort studies that investigate associations between clearly defined operationalizations of psychological stress and cognitive outcomes are highly encouraged to make more definite conclusions possible.

Finally, there were four factors (visual impairment, anxiety, tooth loss, and olfactory impairment) that were identified as potentially important factors in the umbrella review (frequently reported with consistent results), but were scored rather low during the second Delphi round. This discrepancy might be caused by “confirmation bias” toward factors that are more commonly described in recent reports on modifiable dementia risk factors (e.g., the 2020 report of the Lancet Commission on Dementia Prevention, Intervention, and Care or the 2019 WHO Guidelines on Risk Reduction of Cognitive Decline and Dementia). As a result, the importance of the aforementioned four factors might have been underestimated.

#### 4.2.3 Modifiable factors suitable for population‐level dementia risk reduction

Some of the factors that were encountered in the first Delphi round and the umbrella review were excluded from further evaluation because they were not considered suitable for dementia risk reduction on an individual level through self‐management or primary care. However, those factors still may be great entry points for risk reduction on a population level. For instance, while the risk of traumatic brain injury (TBI) can be modified (e.g., wearing a helmet when cycling), it is not possible to reverse the effect after its first occurrence.^123^ This factor would, therefore, have a static negative impact on someone's dementia risk score, without being amenable to change and providing room for improvement (except for preventing recurring TBI). For similar reasons, cerebrovascular disorders were not included as a risk factor in the original LIBRA index.^17^ Formal education was also excluded from further evaluation, as it is less amenable to change after adolescence.^3^ Instead, the LIBRA index focuses on encouraging cognitively stimulating activities at midlife. Although there is reasonable evidence for associations between various air pollutants and dementia, this field of research mainly focuses on ambient outdoor exposure, over which there is little personal control and which tends to be correlated with socioeconomic position.^124^ Despite its limited modifiability on an individual level, outdoor air pollution remains an urgent public health priority that should be addressed by policy makers, especially considering that the WHO estimates that 99% of the global population breathes air that exceeds the WHO guideline limits.^125^ In conclusion, the degree of modifiability has prompted us to exclude certain factors from consideration for an updated LIBRA index. However, such factors should be considered alongside individualized indices as part of a multi‐level approach to dementia prevention.

### 4.3 Strengths and weaknesses

The main strength of our research design is that quantitative data from an extensive umbrella review including prospective cohort studies was combined with quantitative and qualitative data from 18 world‐renowned dementia experts. As such, we acquired the best possible evidence with regard to emerging etiological factors for dementia and cognitive decline, enabling us to prioritize factors for incorporation in dementia risk scores and risk reduction strategies. Second, the extensive search strategy of our umbrella review ensured wide coverage of the literature by comparing primary studies included in all encountered SR and MA. Third, expert opinions on different operationalizations of multidimensional factors such as sleep and social contact provided insight into how these factors could be best implemented in daily practice. Finally, the relatively high number of experts included compared to the previous Delphi study,^17^ both with and without clinical background, ensured that different perspectives and research areas were well represented.

Despite the scrupulous design of our study, limitations remain. First, the umbrella review limited itself to SR/MA including observational studies, which are more vulnerable to methodological biases, such as publication bias, information bias, reverse causation (as some studies have short follow‐up times), selection bias, and confounding, compared to RCTs.^126^ However, because we applied strict inclusion and exclusion criteria to the included primary studies of each SR/MA, we selected only the most suitable studies for causal inference. We realize that by excluding RCTs, some information was lost. Considering the nature of our methods, we decided to limit the review to observational studies for improved comparability among the identified factors, as RCTs are subject to short follow‐up times, small sample sizes, limited generalizability, and not all factors can be subjected to RCTs. Second, as we conducted an umbrella review that included other SR/MA, our coverage of the primary literature was dependent on the inclusion and exclusion criteria applied in those SR/MA. As a result, it remains possible that some primary literature has not been included in our umbrella review. However, considering our wide search strategy we expect the amount of missed literature to be limited. Third, we only took the direction of effect sizes into account, and not their magnitude, level of adjustment (as individuals frequently have multiple risks), nor the size of the populations from which they were computed. As such, the consistency that we used to order the encountered risk factors might have been low, despite large, well‐designed studies or MA reporting significant effect sizes. By cross‐validating the findings from the umbrella review in the Delphi study, we limited the methodological bias that might have arisen in our review. Fourth, because multiple ethnic minorities are underrepresented across literature addressing dementia risk factors, the generalizability of our results is limited to a selected group of Western individuals living in middle‐ and high‐income economies. Fifth, despite our efforts to include Delphi experts from diverse backgrounds, most participants originated from high‐income countries. Additionally, it is possible that seminal reports on modifiable factors (like the 2020 report of the Lancet Commission on Dementia Prevention, Intervention, and Care or the 2019 WHO dementia risk reduction guidelines) might have biased the experts’ opinions toward particular factors. As such, not well‐known factors and factors that might be particularly helpful for dementia risk reduction in low‐ and middle‐income countries might have been underrepresented in this study. Sixth, interactions between modifiable factors and non‐modifiable factors (e.g., biological sex, age, genetics) were not considered in this study. Therefore, it remains to be determined whether the (magnitudes of the) associations are the same within different populations. This makes a strong case for unravelling severity of risk factors, gene–environment interactions, and epigenetic mechanisms, which might explain the potentially differential effects of specific risk factors and indices on the risk for dementia or cognitive decline in distinct genetic strata.

### 4.4 Knowledge gaps and future directions

This Delphi study and umbrella review highlights that there is considerable high‐quality observational evidence for the factors previously included in the LIBRA index and new candidate risk and protective factors for dementia risk reduction. However, for some multidimensional factors, it's not entirely clear which operationalization should be targeted. Therefore, we would benefit from more long‐term cohort studies investigating the association between clearly defined operationalizations of the multidimensional factors social contact and sleep with dementia or cognitive decline, to identify the most impactful components and make more tailored recommendations with regard to brain health promotion. Additionally, the vast majority of dementia prevention literature focuses on mid‐ and late‐life exposure, even though exposure to many dementia risk factors starts or even peaks throughout early adulthood and prolongs into very late life. This highlights the importance of future initiatives addressing brain health from a life course perspective to better tailor preventive strategies to specific age groups.^127^, ^128^

Despite the rather consistent observational evidence for the factors highlighted in the current study, the evidence from RCTs intervening on these factors is limited. Some clinical trials that address single modifiable risk factors in selected groups have found success (e.g., pharmacological treatment of hypertension), while others didn't (e.g., pharmacological treatment of type 2 diabetes or hypercholesterolemia).^129^ Whenever possible, more well‐designed RCTs are needed to establish that there are indeed causal, reversible associations between some of the modifiable factors and dementia. It should be noted, however, that some of these factors could never be the subject of RCTs because of practical or ethical restraints (e.g., smoking, TBI, social contact, etc.) and some of the most successful public health interventions, like smoking cessation, have taken place without RCTs. As for the most relevant factors identified in this study, these will be tested for incorporation in an updated version of the LIBRA index. We plan to externally validate the updated LIBRA index in prospective (population‐based) cohort studies to compare its predictive validity to the original LIBRA index.

To date, multiple studies have been undertaken to assess the effect of multifactorial lifestyle interventions on cognitive decline and dementia. One of the first was the Prevention of Dementia by Intensive Vascular Care (preDIVA) trial, which found no reduction of all‐cause dementia risk after a 6‐year multidomain cardiovascular intervention in a non‐selected population.^130^ Similarly, the Multidomain Alzheimer Preventive Trial (MAPT) investigating the effect of a multidomain intervention, either alone or combined with omega 3 polyunsaturated fatty acid supplementation, did not show a reduction in cognitive decline over a 3‐year period in people recruited from memory clinics.^131^ In contrast, the Finnish Geriatric Intervention Study to Prevent Cognitive Impairment and Disability (FINGER) trial is currently being conducted in multiple countries around the world (World‐Wide FINGER network) after a successful trial in Finland that showed that a multidomain lifestyle intervention reduced the rate of cognitive decline compared to a control group that received regular lifestyle advice.^132^ Alongside validating the factors identified in the current study in RCTs and prioritizing them for public health, the challenge remains to translate this knowledge into societal action at appropriate levels, whether at population, community, household, or individual levels. This highlights the need for close collaboration between diverse players in all our societies across research disciplines but also policy makers, third and fourth sectors to health care and public health, to create an environment supporting sustainable lifestyle changes.

## 5 CONCLUSION

Based on the triangulation of all the current evidence, hearing impairment, social contact, and sleep have strong evidence for integration in risk scores and preventive interventions addressing brain health on the individual level. Simultaneously, more research on the different components of social contact and sleep is needed to identify the most suitable targets, considering their multidimensionality. Future studies will use these factors in an updated version of the LIBRA index for external validation. While life course inequalities, TBI, education, and air pollution were considered less suitable as targets for individualized dementia risk reduction, they appear to be relevant for population‐level risk reduction.

## CONFLICT OF INTEREST STATEMENT

The authors declare no conflicts of interest. Author disclosures are available in the supporting information.

## Supporting information

Supporting Information

Supporting Information

Supporting Information

Supporting Information

Supporting Information

Supporting Information

Supporting Information

Supporting Information

Supporting Information

Supporting Information

Supporting Information
