# Supplementary material for: Umbrella review and Delphi study on modifiable factors for dementia risk reduction
Source: Alzheimers Dement. 2023 Dec 30;20(3):2223–39. doi: 10.1002/alz.13577 (PMC10984497; doi:10.1002/alz.13577)
Supplement: Supplementary file 1 — Supporting Information [file ALZ-20-2223-s003.docx]

**Appendix A: Umbrella Review Search Strategies**Below, the different search strategies for the four different databases can be found, together with the number of hits for each database. Database-specific filters or restrictions are indicated separately underneath each strategy. Some Boolean operators are indicated in red to distinguish search terms relating to exposures, outcomes, and publication types. All of the databases were searched on the 23^rd^ of May 2021

**PubMed | 1,793 hits**

(risk factor [ALL] **OR** epidemiologic factor [MESH] **OR** expos* [ALL] **OR** determinant* [ALL] **OR** predict* [ALL]) **AND** (dementia [ALL] **OR** alzheimer*[ALL] **OR** “cognition disorders” [MESH:noexp] **OR** “pre-clinical AD” [ALL] **OR** “preclinical AD” [ALL] **OR** ((cognitive* **OR** cognition **OR** memory) **AND** (declin* **OR** impair* **OR** deteriora* **OR** change* **OR** deficit* **OR** complaint))) **NOT** (schizophrenia [ALL] **OR** “down syndrome” [ALL] **OR** “psychotic disorders” [MESH] **OR** “psychosis” [ALL] **OR** “substance-related disorders” [MESH] **OR** “substance abuse” [ALL]**OR** epilepsy [ALL] **OR** “seizure disorder” [ALL] **OR** “Parkinson disease” [ALL] **OR** “bipolar disorder” [ALL]) **AND** (systematic [SB] **OR** “meta-analysis” [PT]) **AND** ((Dutch [LANG] **OR** English [LANG]) **AND** (“2015/01/01” [PDAT] : “2021” [PDAT]))

**Embase | 2,097 hits**

(risk factor/ **OR** epidemiology/ **OR** (expos* or determinant* or predict*).af.) **AND** (dementia/ **OR** cognitive defect/ **OR** alzheimer disease/ **OR** (dementia **OR** alzheimer* **OR** pre-clinical AD **OR** preclinical AD **OR** ((cognitive* **OR** cognition **OR** memory) **AND** (declin* **OR** impair* **OR** deteriora* **OR** change* **OR** deficit* **OR** complaint))).af.) **NOT** (exp schizophrenia/ **OR** down syndrome/ **OR** exp psychosis/ **OR** exp drug dependence/ **OR** exp epilepsy/ **OR** exp bipolar disorder/ **OR** (schizophrenia **OR** down syndrome **OR** psychosis **OR** substance abuse **OR** epilepsy **OR** epilepsy **OR** seizure disorder **OR** Parkinson disease **OR** bipolar disorder).af.)

***limit x*** to ((meta analysis **OR** "systematic review") **AND** (Dutch **OR** English) **AND** yr="2015 – Current")

**Web of Science | 2,275 hits**

ALL = ("risk factor*" **OR** epidemiolog* **OR** expos* **OR** determinant* **OR** predict*) **AND** ALL = (dementia **OR** "cognitive defect*" **OR** Alzheimer* **OR** "pre-clinical AD" **OR** "preclinical AD" **OR** ((cognitive* **OR** cognition **OR** memory) **AND** (declin* **OR** impair* **OR** deteriora* **OR** change* **OR** deficit* **OR** complaint))) **NOT** ALL = (schizophrenia **OR** "down syndrome" **OR** "psychosis" **OR** "drug dependence" **OR** "epilepsy" **OR** "bipolar disorder" **OR** "substance abuse" **OR** "seizure disorder" **OR** "Parkinson disease") **AND** ALL = ("systematic review" **OR** "meta analysis" **OR** "meta-analysis")

***Restrict results by languages:*** “English” **OR** “Dutch” **AND** custom time range: 2015-2020

**PsychINFO | 375 hits**

MA (dementia **OR** Alzheimer’s **OR** cognitive impairment **OR** memory loss) **AND** MR (systematic review **OR** meta analysis)

***Published date:*** 20150101 – 20201231

**Total | 6,540 hits**
