# Supplementary material for: Umbrella review and Delphi study on modifiable factors for dementia risk reduction
Source: Alzheimers Dement. 2023 Dec 30;20(3):2223–39. doi: 10.1002/alz.13577 (PMC10984497; doi:10.1002/alz.13577)
Supplement: Supplementary file 2 — Supporting Information [file ALZ-20-2223-s005.docx]

**Appendix B: Data Extraction Spreadsheet Sample**Below, an example can be found of the spreadsheet that was used for extracting the data, together with some guiding information on how each field was completed. These study characteristics were extracted from the systematic review or meta-analysis (SR/MA) and always double-checked in the primary studies whenever available. In case of discrepancies, the values reported in the primary studies were leading.


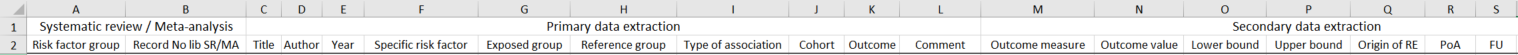


**Risk factor group**The different SR/MA were topically categorized for easy comparison of their included primary literature within the spreadsheet. Whenever the SR/MA were not topic-specific, they were categorized in a separate “combination” category. Those SR/MA were the last ones to be analyzed within our umbrella review, to see whether they included primary literature that was not included in the topic-specific SR/MA.

**Record No lib SR/MA**Each SR/MA which was imported into Endnote automatically got an identification number assigned. We continued using these numbers for the identification of the related SR/MA.

**Title**The title of the included primary literature for a particular SR/MA.

**Author**First author of the included primary study.

**Year**Year that the included primary study was published.

**Specific factor**The specific factor(s) of that included primary study which was/were investigated in the related SR/MA. We limited the amount of factors discussed for a primary study to one, unless there were multiple SR/MA investigating different aspects of that particular primary study. More detailed information on this approach can be found in Appendix C.

**Exposed group**The reported definition of the exposed group for that particular risk or protective factor within the primary study (example: systolic blood pressure ≥ 140 mmHg). Whenever no categorical exposure was reported, we completed this field with the continuous increase or decrease in that particular factor (example: systolic blood pressure + 10 mmHg).

**Reference group**The reported definition of the reference group for that particular risk or protective factor within the primary study (example: systolic blood pressure < 120 mmHg). There was no reference group in the case of continuous exposure variables.

**Type of association**For each exposure, we determined whether that particular risk or protective factor is significantly associated with increased or decreased risk of dementia/cognitive impairment/cognitive decline based on the columns *outcome value, lower bound,* and *upper bound*. This categorization formed the basis of our analyses.

**Cohort**The cohort in which the primary study was conducted. Whenever multiple primary studies reported similar exposure-outcome associations for the same cohort, we (CR, SK, MvB, KD) discussed which study to select based on other study characteristics and their methodological approach. As such, we prevented double counting highly similar studies using the same cohort to reduce bias.

**Outcome**The outcome which was assessed within the primary study (dementia, cognitive impairment, cognitive decline, …). Studies that did not report a binary outcome for cognition or which did not report longitudinal changes in cognition were omitted.

**Comment**Free text field used for particularities within that primary study (example: study limited to women) or for reporting the instrument and threshold value used for assessing cognitive impairment (whenever no formal dementia diagnosis was used).

**Outcome measure**The outcome measure used for assessing the exposure-outcome relationship. Binary measures were preferred (relative risks, hazard ratios, odds ratios), otherwise, continuous measures were used (regression coefficients).

**Outcome value**The value for the *outcome measure* reported in the SR/MA or primary source in case of discrepancy.

**Lower bound**The value for the lower bound of the *outcome value* reported in the SR/MA or primary source in case of discrepancy.

**Upper bound**The value for the upper bound of the *outcome value* reported in the SR/MA or primary source in case of discrepancy.

**Origin of RE**Indication whether the outcome value and corresponding lower and upper bound were only identified in the SR/MA or whether these outcomes were confirmed by us in the primary study.

**Population for analysis (study selection)**The number of participants reported in the SR/MA or primary source in case of discrepancy. Studies with a population of <200 participants were omitted.

**FU (study selection)**The number of follow-up years reported in the SR/MA or primary source in case of discrepancy. Studies with a follow-up of <2 years were omitted.

**Follow up estimator**Indication whether the follow-up time was expressed as a mean, median, maximum, or approximation (in case it was not specified).

**Age**The mean age of the study population. Approximated in cases where no overall mean for the whole population was provided (rounded down to the nearest 5-year “interval” based on the youngest reported sub-group). Based on age, exposures with considerable evidence for a life-course approach were categorized in either midlife exposure (≤65 years) or late-life exposure (>65 years)
