# Supplementary material for: Umbrella review and Delphi study on modifiable factors for dementia risk reduction
Source: Alzheimers Dement. 2023 Dec 30;20(3):2223–39. doi: 10.1002/alz.13577 (PMC10984497; doi:10.1002/alz.13577)
Supplement: Supplementary file 3 — Supporting Information [file ALZ-20-2223-s010.docx]

**Appendix C: Data Extraction and Harmonisation Procedures**

**General**There are multiple ways in which data extraction and harmonization can be approached. To ensure full replicability of our approach, our methods for data extraction and harmonization are discussed in this appendix for every risk or protective factor independently. To ensure that each primary study was only included once in our table of associations (unless multiple aspects of that study were discussed in different systematic reviews/meta-analyses), there were a couple of general rules that applied to all factors, including the following:

1. Whenever both a categorical model and a continuous model were reported for a particular exposure, we used the categorical model to determine the association between exposure and cognitive outcome. **Example**: If a study reported both the risk estimates for *dichotomized hypertension* (categorical model) and an *increase of 10mmHg in systolic blood pressure* (continuous model), then the risk estimate for dichotomized hypertension was used to determine the association between exposure and cognitive outcome.
2. Whenever more exposure levels were discussed in a categorical model, we selected extrema with the biggest contrast for determining the risk estimate. **Example**: If a study compared *stage I hypertension* with *normotensive individuals* and *stage II hypertension* with *normotensive individuals*, then the risk estimate for stage II hypertension was used to determine the association between exposure and cognitive outcome.
3. Whenever there was substantial evidence of a U-shaped relationship between the exposure and outcome, we compared the extrema on both sides with the reference in the middle, such that two associations were included from the same primary study in our review. **Example**: If a study compared both *underweight* and *obesity* with *normal weight*, then the risk estimates for both exposures were used to determine the associations between both exposures and cognitive outcome.
4. Whenever risk estimates were reported for multiple cognitive outcomes, we choose the most generalizable outcome to determine the association between exposure and cognitive outcome. We considered *(all-cause) dementia* the most generalizable outcome, followed by *Alzheimer’s Disease (AD)*, *vascular dementia (VaD)*, *other dementia*, *(mild) cognitive impairment (MCI),* and *cognitive decline*. Whenever studies did not formally assess a binary cognitive outcome, the study must have assessed cognitive decline (as a change in cognitive performance at two or more time points) to be eligible for inclusion. **Example:** If a study reports both the risk estimates for the effect of stage II hypertension on all-cause dementia and MCI, then the risk estimate for all-cause dementia was used to determine the association between exposure and cognitive outcome. Studies assessing dementia-related mortality, progression to dementia from MCI, and cognitive performance were excluded.
5. When a systematic review/meta-analysis (SR/MA) focussed on the relationship between the exposure and a dementia subtype (mainly AD or VaD), but the primary studies also report risk estimates for all-cause dementia, we used the risk estimate for all-cause dementia to determine the association between exposure and cognitive outcome.
6. The studies needed to have a clear prospective, longitudinal design. As such, retrospective studies, data-linkage studies, registry studies, studies solely based on insurance databases, and case-control studies nested in a prospective cohort were excluded. Cohort studies that used registries/databases only for the assessment of cognitive outcomes, were included.
7. When there was a discordance between the conclusion of a SR/MA reporting a primary study and the conclusion of the primary study itself, we analyzed the primary study to ensure that the most appropriate risk estimate was included. The risk estimate from the most adjusted model with the biggest contrast between exposure and reference was considered to be the most appropriate.
8. Whenever the SR/MA distinguished between midlife exposure and late-life exposure, we registered this dichotomization as well. Whenever the SR/MA did not make this distinction, we dichotomized midlife exposure (<65 years old) and late-life exposure (≥65 years old) based on the mean baseline age of the participants reported in the SR/MA. This was only done for the factors with substantial evidence for a life course approach: blood pressure, cholesterol, and BMI.

**Alcohol**

The amount of alcohol consumption was **reported in multiple ways** (times/week, drinks/day, g/day, etc.) In our table of associations, we defined high alcohol consumption as the group of individuals reporting the highest amount of alcohol consumption compared with the group of individuals reporting the lowest amount of alcohol consumption (excluding the abstainer’s group). Whenever a study also reported the effect for abstinence, this was included as an independent factor.

**Blood pressure**Because there is substantial evidence that the effect of blood pressure might differ over the life course, a distinction was made between midlife and late-life exposure. When a primary study reports on both midlife and late-life exposure, both associations were included in our table of associations.
Constructs of blood pressure encountered in the literature: **hypertension, systolic blood pressure (SBP), diastolic blood pressure (DBP), orthostatic hypertension (OH)**. SBP and DBP were evaluated as sub-constructs of hypertension, OH was evaluated as an independent construct. In rare cases, low SBP/DBP was reported as the exposure of interest, those were also evaluated as independent constructs. The decision process for selecting the most appropriate associations:

1)Was “*hypertension*” reported as exposure?

If yes: Use risk estimate for “*hypertension*” 🡪 Reported as hypertension in our table of associations

If no: Continue to question 2

2)Was “*SBP*” reported as exposure?

If yes: Continue to question 3

If no: Continue to question 4

3)Was dichotomized “*high SBP*” reported as exposure?

If yes: Use risk estimate for “*high SBP*” as exposure 🡪 Reported as hypertension in our table of associations

If no: Use risk estimate for “*x increase in SBP*” as exposure 🡪 Reported as hypertension in our table of associations

4)Was dichotomized “high DBP” reported as exposure?

If yes: Use risk estimate for “*high DBP*” as exposure 🡪 Reported as hypertension in our table of associations

If no: Use risk estimate for “*x increase in DBP*” as exposure 🡪 Reported as hypertension in our table of associations

**Cholesterol**Because there is substantial evidence that the effect of cholesterol might differ over the life course, a distinction was made between midlife and late-life exposure. When a primary study reports on both midlife and late-life exposure, both associations were included in our table of associations.
Constructs of cholesterol encountered in the literature: **total cholesterol, high-density lipoprotein cholesterol (HDL-C), low-density lipoprotein cholesterol (LDL-C), and triglycerides.** HDL-C, LDL-C, and triglycerides were all evaluated as sub-constructs of total cholesterol. The decision process for selecting the most appropriate associations:

1)Was “*high total cholesterol*” reported as exposure?

If yes: Use risk estimate for “*high total cholesterol*” 🡪 Reported as high total cholesterol in our table of associations

If no: Continue to question 2

2)Was “*high HDL-C*” reported as exposure?

If yes: Use risk estimate for “*High HDL-C*” as exposure 🡪 Reported as high HDL-C in our table of associations

If no: Continue to question 3

3)Was “*High LDL-C*” reported as exposure?

If yes: Use risk estimate for “*high LDL-C*” as exposure 🡪 Reported as high LDL-C in our table of associations

If no: Use risk estimate for “*high triglycerides*” as exposure 🡪 Reported as high triglycerides in our table of associations

**Cognitive activity**

Cognitive activity was encountered in the literature in **numerous different forms**. It can entail high engagement in **one particular form of cognitive activity** (for example playing a musical instrument vs not playing an instrument) or high engagement in a **composite of stimulating leisure activities**. Whenever both the risk estimates for engagement in one particular activity and engagement in a composite were reported, the risk estimate for the composite was used to assess the relationship between exposure and cognitive outcome. All the encountered constructs were reported as high engagement in cognitively stimulating activities in our review.

**CVDs | Atrial fibrillations**

The presence of atrial fibrillations was **always clearly dichotomized** (present vs absent) in the included studies. Therefore, we did not apply any harmonisation processes ourselves.

**CVDs | Coronary heart disease**

Coronary heart diseases (CHD) were encountered in the literature in three main constructs: **coronary heart diseases (CHD), myocardial infarction (MI), and angina pectoris (AP)**. Whenever a risk estimate was reported for CHD, this risk estimate was used to assess the relationship between exposure and cognitive outcome. Otherwise, the risk estimate for MI was used, followed by AP. All of these constructs were reported as CHD in our review.

**CVDs | Heart failure**

The presence of heart failure was **always clearly dichotomized** (present vs absent) in the included studies. Therefore, we did not apply any harmonisation processes ourselves.

**Diet | Caffeine**

Constructs of caffeine consumption encountered in the literature: **caffeine consumption, coffee consumption, (green) tea consumption.** There were only a couple of studies that reported on overall intake of caffeine (in mg/day), whilst the majority of studies focussed on either tea and/or coffee consumption (in units/day or times/week). Whenever a study reported on green tea consumption, this was considered to be an independent factor. A study could have been included in our table of associations for all three beverage constructs independently, depending on what was reported in the systematic review or meta-analysis. The decision process for selecting the most appropriate associations:

1)Was “*high caffeine consumption*” reported as exposure?

If yes: Use risk estimate for “*high caffeine consumption*” 🡪 Reported as high caffeine consumption in our table of associations

If no: Continue to question 2

2)Was only *“high (green) tea consumption*” **or** “*high coffee consumption*” reported as exposure?

If yes: Use risk estimate for “*high (green )tea consumption*” **or** “*high coffee consumption*” 🡪 Reported as high (green) tea consumption **or** high coffee consumption respectively

If no: Use risk estimate for “*high (green) tea consumption*” **and** “*high coffee consumption*” 🡪 Reported as high (green) tea consumption **and** high coffee consumption respectively

**Diet | Coffee**

Same procedure applied as indicated under the heading “Diet | Caffeine”.

**Diet | Dairy**

Dairy consumption was encountered in the literature in as either **general dairy consumption** or **consumption of specific dairy products**. When a risk estimate for the former was reported, this risk estimate was used to assess the relationship between exposure and cognitive outcome. When risk estimates were reported for specific dairy products only, we used the risk estimate for milk consumption (and variations like milk and yogurt consumption) to assess the relationship between exposure and cognitive outcome. Both constructs were reported as high dairy consumption in our review.

**Diet | Fish**

Fish consumption was **always clearly dichotomized** (high vs low consumption) in the included studies. Therefore, we did not apply any harmonisation processes ourselves.

**Diet | Fruit and vegetable consumption**

Fruit and vegetable consumption was encountered in the literature as **fruit consumption**, **vegetable consumption**, or **fruit and vegetable consumption**. All three of these constructs were reported as “high fruit and vegetable consumption” in our review. In cases where low fruit and vegetable consumption was reported as a risk factor, we inverted the relationship such that high fruit and vegetable consumption was reported as a protective factor in our review.

**Diet | Lipids**

Constructs of lipid consumption encountered in the literature: **total fat consumption**, **saturated fat consumption**, **mono-unsaturated fat consumption**, **poly-unsaturated fat consumption**. The latter two constructs were reported very sparingly and were, therefore, beyond the scope of this review. The former two were considered as high total fat consumption and high saturated fat consumption independently in our review.

**Diet | Meat**

Constructs of meat consumption encountered in the literature: **total meat consumption**, **poultry consumption**, and **red- and processed meat consumption**. The former two constructs together were considered as high meat consumption, whilst the latter was considered as high red- and processed meat consumption in our review.

**Diet | Mediterranean**

Adherence to Mediterranean diet (MeDi) was **always clearly dichotomized** (high vs low adherence) in the included studies. In the rare cases where low adherence to MeDi was reported as a risk factor, we inverted the relationship such that high adherence to MeDi was reported as a protective factor. In some studies, other dietary patterns and scores, such as the Dietary Approaches to Stop Hypertension (DASH) diet, Mediterranean-DASH (MIND) diet, or Healthy Eating Index (HEI) were investigated. Considering that the vast majority of studies included in our umbrella review focussed on the MeDi pattern, we omitted the other patterns (which had a maximum of three occurrences) from further analysis in our umbrella review.

**Diet | Nuts**

Nut consumption was **always clearly dichotomized** (high vs low consumption) in the included studies. Therefore, we did not apply any harmonisation processes ourselves.

**Diet | Omega-3 fatty acids**

The majority of studies overlapped with the heading “Diet | Fish”. For the ones that did not overlap, total omega-3 fatty acid intake was **always clearly dichotomized** (high vs low consumption) in the included studies. Therefore, we did not apply any harmonisation processes ourselves.

**Diet | Tea**

Same procedure applied as indicated under the heading “Diet | Caffeine”.

**Medical | Diabetes**

Diabetes status was reported as either **pre-diabetes/impaired fasted blood glucose** or **diabetes**. Both constructs were **always clearly dichotomized** (present vs absent), however, some studies assessed both constructs, whilst others only assessed one of them. Whenever both constructs were reported in a study, they were independently included in our review. The majority of studies explicitly reported the risk estimate for type-2 diabetes (T2D), whilst other studies did not clearly report the subtype of diabetes. It is most likely that those latter studies still assessed the effect of type-2 diabetes, considering that the average baseline age within the included cohorts can be classified as midlife or older. Therefore, all of the studies were reported as T2D in our review.

**Medical | Metabolic syndrome**

The presence of metabolic syndrome was **always clearly dichotomized** (present vs absent) in the included studies. Therefore, we did not apply any harmonisation processes ourselves.

**Medical | Chronic kidney disease**

Impaired kidney function was encountered in the literature in two main constructs: **Glomerular filtration rate (GFR), and proteinuria**. Whenever a risk estimate was reported for GFR, this risk estimate was used to assess the relationship between exposure and cognitive outcome. Otherwise, the risk estimate for proteinuria was used, followed by other variables for assessing impaired kidney function. All of these constructs were reported as impaired kidney function in our review.

**Obesity/BMI**

Because there is substantial evidence that the effect of BMI might differ over the life course, a distinction was made between midlife and late-life exposure. BMI was mainly categorically reported as either **underweight** or **obesity**. Both constructs were **always clearly dichotomized** (present vs absent), however, some studies assessed both constructs, whilst others only assessed one of them. Whenever both constructs were reported in a study, they were independently included in our review. In rare cases, only a continuous model was reported investigating the relationship between a continuous increase in BMI and cognitive outcome. Therefore, we also reported high BMI in our table of associations besides the categorical exposures.

**Oral health**

Oral health was mainly reported as either **tooth loss** or **periodontal disease (PD)**. Other constructs were reported very sparingly and were, therefore, beyond the scope of this review. Both constructs were **always clearly dichotomized** (high degree of tooth loss/PD present vs low degree of tooth loss/PD absent), however, some studies assessed both constructs, whilst others only assessed one of them. Whenever both constructs were reported in a study, they were independently included in our review. In the rare cases where tooth retention was reported as a protective factor, we inverted the relationship such that tooth loss was reported as a risk factor in our review.

**Physical activity**

Physical activity (PA) was mainly reported as either **leisure time PA** or **mixed PA**. Other constructs were reported very sparingly and were, therefore, beyond the scope of this review. Whenever both constructs were reported in a study, they were independently included in our review.

**Psychological | Anxiety**

The presence of anxiety was **always clearly dichotomized** (present vs absent) in the included studies. Therefore, we did not apply any harmonisation processes ourselves

**Psychological | Depression**

The presence of depression was **almost always clearly dichotomized** (present vs absent) in the included studies. Therefore, we did not apply any harmonisation processes ourselves. In rare cases, only a continuous model was reported, investigating the relationship between a continuous increase in depressive symptomatology and cognitive outcome. In such a situation, an increase in depressive symptomatology was reported as depression in our review.

**Psychological | Stress**

The presence of psychological stress was **almost always clearly dichotomized** (present vs absent) in the included studies. Therefore, we did not apply any harmonisation processes ourselves. In rare cases, only a continuous model was reported, investigating the relationship between a continuous increase in psychological stress and cognitive outcome. In such a situation, an increase in stress score or proneness was reported as high psychological stress in our review.

**Sedentary behaviour**

The presence of sedentary behaviour was **always clearly dichotomized** (present vs absent) in the included studies. Therefore, we did not apply any harmonisation processes ourselves.

**Sensory functioning | Hearing impairment**

The presence of hearing impairment was **almost always clearly dichotomized** (present vs absent) in the included studies. Therefore, we did not apply any harmonisation processes ourselves. In rare cases, only a continuous model was reported, investigating the relationship between a continuous increase in hearing threshold and cognitive outcome. In such a situation, an increase in hearing threshold was reported as hearing impairment in our review.

**Sensory functioning | Olfactory impairment**

The presence of olfactory impairment was **almost always clearly dichotomized** (present vs absent) in the included studies. Therefore, we did not apply any harmonisation processes ourselves. In rare cases, only a continuous model was reported, investigating the relationship between a continuous increase in olfactory threshold and cognitive outcome. In such a situation, an increase in olfactory threshold was reported as olfactory impairment in our review.

**Sensory functioning | Visual impairment**

The presence of visual impairment was **always clearly dichotomized** (present vs absent) in the included studies. Therefore, we did not apply any harmonisation processes ourselves.

**Sleep | Duration**

Sleep duration was mainly categorically reported as either **short sleep** or **long sleep**. Both constructs were **almost always clearly dichotomized** (present vs absent), however, some studies assessed both constructs, whilst others only assessed one of them. Whenever both constructs were reported in a study, they were independently included in our review. In rare cases, only a continuous model was reported investigating the relationship between a continuous increase/decrease in sleep and cognitive outcome. In such a situation, an increase in sleep duration was reported as long sleep duration and a decrease in sleep duration was reported as short sleep duration.

**Sleep | General**

Sleep was encountered in the literature in **numerous different forms**. Some aspects of sleep were particularly frequently reported, such as **insomnia** and **daytime sleepiness**. Those were respectively reported as insomnia and daytime sleepiness in our review. All other constructs like **(self-reported) sleep problems**, **(self-reported) poor sleep**, **sleep fragmentation**, **sleep-related movement disorders**, and variations on these constructs were reported as poor sleep quality in our review. The decision process for selecting the most appropriate associations:

1)Was “*insomnia*” and/or “*daytime sleepiness*” reported as exposure?

If yes: Use risk estimate for *“insomnia*” and/or “*daytime sleepiness*” as exposure 🡪 Reported as insomnia and/or daytime sleepiness respectively in our table of associations

If no: Continue to question 2

2)Was “*(self-reported) sleep problems*” or “*(self-reported) sleep quality*” reported as exposure?

If yes: Use risk estimate for “*(self-reported) sleep problems*” or “*(self-reported) sleep quality*” as exposure 🡪 Reported as poor sleep quality in our table of associations

If no: Use risk estimate for other reported sleep construct as exposure 🡪 reported as poor sleep quality in our table of associations

**Sleep | SDB**

The presence of sleep-disordered breathing (SDB) was **always clearly dichotomized** (present vs absent) in the included studies. Therefore, we did not apply any harmonisation processes ourselves.

**Smoking**

Smoking was **always clearly dichotomized** (smoker vs non-smoker) in the included studies. Therefore, we did not apply any harmonisation processes ourselves.

**Social contact**

Social contact was encountered in the literature in **numerous different forms**. The forms most frequently encountered were **low social activities**, **social (dis)engagement**, **loneliness**, **low social network size**, **living alone**, **low emotional support**, and **social vulnerability**. All of these constructs were independently included in our review, however, the first two constructs were combined into one category (social engagement). All constructs were considered risk factors, such that we inverted the relationships for high social engagement, high social network size, and high emotional support.
