# Supplementary material for: Umbrella review and Delphi study on modifiable factors for dementia risk reduction
Source: Alzheimers Dement. 2023 Dec 30;20(3):2223–39. doi: 10.1002/alz.13577 (PMC10984497; doi:10.1002/alz.13577)
Supplement: Supplementary file 4 — Supporting Information [file ALZ-20-2223-s002.docx]

**Appendix D: Quality Assessment of Included Studies
Supplementary table 1:** Quality assessment of the 148 systematic reviews (SR) and meta-analyses (MA) included in our umbrella review.

| **Study** | **SR or MA** | **1. Is the review based on a focused question that is adequately formulated and described?** | **2. Were eligibility criteria for included and excluded studies predefined and specified?** | **3. Did the literature search strategy use a comprehensive, systematic approach?** | **4. Were titles, abstracts, and full-text articles dually and independently reviewed for inclusion and exclusion to minimize bias?** | **5. Was the quality of each included study rated independently by two or more reviewers using a standard method to appraise its internal validity?** | **6. Were the included studies listed along with important characteristics and study results of each study?** | **7. Was publication bias assessed?** | **8. Was heterogeneity assessed? (This question applies only to meta-analyses.)** | **Total score** | **Max score** | **% Score** |
| --- | --- | --- | --- | --- | --- | --- | --- | --- | --- | --- | --- | --- |
| Lao, 2021 [1] | MA | 1 | 1 | 1 | 1 | 1 | 1 | 1 | 1 | 8 | 8 | 100,00% |
| Wiegmann, 2020 [2] | SR | 1 | 1 | NR | NR | NR | 1 | NR | NA | 3 | 7 | 42,86% |
| Xu, 2017 [3] | MA | 1 | 1 | 1 | NR | NR | 1 | 1 | 1 | 6 | 8 | 75,00% |
| Forte, 2020 [4] | SR | 1 | 1 | 1 | 1 | NR | 1 | NR | NA | 5 | 7 | 71,43% |
| Ou, 2020 [5] | MA | 1 | 1 | 1 | 1 | 1 | 1 | 1 | 1 | 8 | 8 | 100,00% |
| Min, 2020 [6] | MA | 1 | 1 | 1 | 1 | 1 | 1 | 1 | 1 | 8 | 8 | 100,00% |
| Lennon, 2019 [7] | MA | 1 | 1 | 1 | 1 | NR | 1 | 1 | 1 | 7 | 8 | 87,50% |
| Iseli, 2019 [8] | MA | 1 | 1 | 1 | 1 | 1 | 1 | 1 | 1 | 8 | 8 | 100,00% |
| Agarwal, 2020 [9] | SR | 1 | 1 | 1 | NR | NR | 1 | NR | NA | 4 | 7 | 57,14% |
| Anstey, 2017 [10] | MA | 1 | 1 | 1 | 1 | 1 | 1 | 1 | 1 | 8 | 8 | 100,00% |
| Koch, 2016 [11] | SR | 1 | 1 | 1 | NR | NR | 1 | NR | NA | 4 | 7 | 57,14% |
| Walsh, 2021 [12] | MA | 1 | 1 | 1 | 1 | 1 | 1 | NA | NR | 6 | 8 | 75,00% |
| Anderson, 2020 [13] | MA | 1 | 1 | 1 | NR | NR | 1 | 1 | NR | 5 | 8 | 62,50% |
| Brini, 2020 [14] | MA | 1 | 1 | 1 | 1 | 1 | 1 | 1 | 1 | 8 | 8 | 100,00% |
| Mukadam, 2017 [15] | MA | 1 | 1 | 1 | 0 | 1 | 1 | 0 | NR | 5 | 8 | 62,50% |
| Yates, 2016 [16] | MA | 1 | 1 | 1 | 1 | 1 | 1 | NA | 1 | 7 | 8 | 87,50% |
| Sajeev, 2016 [17] | SR | 1 | 1 | 1 | NR | NR | 1 | NR | NA | 4 | 7 | 57,14% |
| Yu, 2020 [18] | MA | 1 | 1 | 1 | 1 | NR | 1 | 1 | 1 | 7 | 8 | 87,50% |
| Hudon, 2020 [19] | MA | 1 | 1 | 1 | 1 | 1 | 1 | NA | 1 | 7 | 8 | 87,50% |
| Olayinka, 2019 [20] | 1 | 1 | 1 | 1 | 1 | 1 | 1 | NR | NR | 6 | 7 | 85,71% |
| Hersi, 2017 [21] | MA | 1 | 1 | 1 | 0 | 0 | 1 | 1 | 1 | 6 | 8 | 75,00% |
| Lafortune, 2016 [22] | SR | 1 | 1 | 1 | NR | 1 | 1 | NR | 1 | 6 | 7 | 85,71% |
| Xu, 2015 [23] | MA | 1 | 1 | 1 | NR | NR | 1 | 1 | 1 | 6 | 8 | 75,00% |
| Proietti, 2020 [24] | MA | 1 | 1 | 1 | NR | NR | 1 | 1 | 1 | 6 | 8 | 75,00% |
| Liu, 2019 [25] | MA | 1 | 1 | 1 | 1 | NR | 1 | 1 | 1 | 7 | 8 | 87,50% |
| Islam, 2019 [26] | MA | 1 | 1 | 1 | 1 | 1 | 1 | 1 | 1 | 8 | 8 | 100,00% |
| Koh, 2022 [27] | MA | 1 | 1 | 1 | 1 | NR | 1 | 1 | 1 | 7 | 8 | 87,50% |
| Liang, 2021 [28] | MA | 1 | 1 | 1 | 1 | NR | 1 | 1 | 1 | 7 | 8 | 87,50% |
| Xia, 2020 [29] | MA | 1 | 1 | 1 | 1 | NR | 1 | 1 | 1 | 7 | 8 | 87,50% |
| Wolters, 2018 [30] | MA | 1 | 1 | 1 | 1 | 1 | 1 | 1 | 1 | 8 | 8 | 100,00% |
| Deckers, 2017 [31] | MA | 1 | 1 | 1 | 1 | NR | 1 | 1 | 1 | 7 | 8 | 87,50% |
| Stefanidis, 2018 [32] | MA | 1 | 1 | 1 | NR | 1 | 1 | NA | 1 | 6 | 8 | 75,00% |
| Li, 2020 [33] | MA | 1 | 1 | 1 | NR | NR | 1 | 1 | 1 | 6 | 8 | 75,00% |
| Cannon, 2017 [34] | MA | 1 | 1 | 1 | 1 | NR | 1 | 1 | 1 | 7 | 8 | 87,50% |
| Chen, 2020 [35] | MA | 1 | 1 | 1 | NR | 1 | 1 | NR | NR | 5 | 8 | 62,50% |
| Larsson, 2018 [36] | MA | 1 | 1 | 1 | 1 | NR | 1 | 1 | 1 | 7 | 8 | 87,50% |
| Wu, 2017 [37] | MA | 1 | 1 | 1 | 1 | NR | 1 | 1 | 1 | 7 | 8 | 87,50% |
| Liu, 2016 [38] | MA | 1 | 1 | 1 | NR | NR | 1 | 1 | 1 | 6 | 8 | 75,00% |
| Cuesta-Triana, 2019 [39] | SR | 1 | 1 | 1 | 1 | 1 | 1 | NR | NA | 6 | 7 | 85,71% |
| Lee, 2018 [40] | MA | 1 | 1 | 1 | 1 | 1 | 1 | NR | 1 | 7 | 8 | 87,50% |
| Wu, 2016 [41] | MA | 1 | 1 | 1 | 1 | 1 | 1 | 1 | 1 | 8 | 8 | 100,00% |
| Bakre, 2018 [42] | MA | 1 | 1 | 1 | 1 | NR | 1 | 1 | NR | 6 | 8 | 75,00% |
| Zeng, 2017 [43] | MA | 1 | 1 | 1 | 1 | NR | 1 | 1 | NR | 6 | 8 | 75,00% |
| Zhang, 2021 [44] | MA | 1 | 1 | 1 | NR | NR | 1 | 1 | 1 | 6 | 8 | 75,00% |
| Mottaghi, 2018 [45] | MA | 1 | 1 | 1 | 1 | NR | 1 | 1 | 1 | 7 | 8 | 87,50% |
| Wu, 2017 [46] | MA | 1 | 1 | 1 | 1 | 1 | 1 | 1 | 1 | 8 | 8 | 100,00% |
| Jiang, 2017 [47] | MA | 1 | 1 | 1 | NR | 1 | 1 | 1 | 1 | 7 | 8 | 87,50% |
| Ran, 2021 [48] | MA | 1 | 1 | 1 | 1 | NR | 1 | 1 | 1 | 7 | 8 | 87,50% |
| Moradi, 2020 [49] | MA | 1 | 1 | 1 | 1 | NR | 1 | 1 | 1 | 7 | 8 | 87,50% |
| Vicente, 2020 [50] | SR | 1 | 1 | 1 | 1 | 1 | 1 | NR | NA | 6 | 7 | 85,71% |
| Liu, 2020 [51] | MA | 1 | 1 | 1 | 1 | NR | 1 | 1 | 1 | 7 | 8 | 87,50% |
| Kheirouri, 2019 [52] | SR | 1 | 1 | 1 | 1 | NR | NR | NR | NA | 4 | 7 | 57,14% |
| Samadi, 2019 [53] | SR | 1 | 1 | 1 | NR | NR | 1 | NR | NA | 4 | 7 | 57,14% |
| Nowson, 2018 [54] | SR | 1 | NR | 1 | 1 | 1 | 1 | NR | NA | 5 | 7 | 71,43% |
| Solfrizzi, 2017 [55] | SR | 1 | 1 | 1 | NR | 0 | 1 | NR | NA | 4 | 7 | 57,14% |
| Yusufov, 2017 [56] | SR | 1 | 1 | 1 | NR | NR | 1 | NR | NA | 4 | 7 | 57,14% |
| Masana, 2017 [57] | SR | 1 | 1 | 1 | 1 | NR | 1 | NR | NA | 5 | 7 | 71,43% |
| Cao, 2016 [58] | MA | 1 | 1 | 1 | 1 | NR | 1 | NA | 1 | 6 | 8 | 75,00% |
| van de Rest, 2015 [59] | SR | 1 | NR | 1 | 1 | NR | 1 | NR | NA | 4 | 7 | 57,14% |
| Panza, 2015 [60] | SR | 1 | NR | 1 | 1 | NR | 1 | NR | NA | 4 | 7 | 57,14% |
| Kim, 2015 [61] | MA | 1 | 1 | 1 | 1 | NR | 1 | 1 | 1 | 7 | 8 | 87,50% |
| Cao, 2019 [62] | MA | 1 | 1 | 1 | 1 | 1 | 1 | 1 | 1 | 8 | 8 | 100,00% |
| Zhang, 2020 [63] | MA | 1 | 1 | 1 | 1 | 1 | 1 | 1 | 1 | 8 | 8 | 100,00% |
| Limongi, 2020 [64] | SR | 1 | 1 | 1 | 1 | 1 | 1 | NR | NA | 6 | 7 | 85,71% |
| Aridi, 2017 [65] | SR | 1 | 1 | 1 | NR | NR | 1 | NR | NA | 4 | 7 | 57,14% |
| Wu, 2017 [66] | MA | 1 | 1 | 1 | 1 | NR | 1 | 1 | 1 | 7 | 8 | 87,50% |
| Petersson, 2016 [67] | SR | 1 | 1 | 1 | 1 | NR | 1 | NR | NA | 5 | 7 | 71,43% |
| Theodore, 2020 [68] | SR | 1 | 1 | 1 | 1 | 1 | 1 | NR | NA | 6 | 7 | 85,71% |
| Wu, 2015 [69] | MA | 1 | 1 | 1 | NR | NR | 1 | 1 | 1 | 6 | 8 | 75,00% |
| Kakutani, 2019 [70] | SR | 1 | 1 | 1 | NR | NR | 1 | NR | NA | 4 | 7 | 57,14% |
| Liu, 2017 [71] | MA | 1 | 1 | 1 | NR | 1 | 1 | 1 | 1 | 7 | 8 | 87,50% |
| Ma, 2016 [72] | MA | 1 | 1 | 1 | 1 | 1 | 1 | 1 | 1 | 8 | 8 | 100,00% |
| Papunen, 2020 [73] | SR | 1 | 1 | 1 | 1 | 1 | 1 | NR | NA | 6 | 7 | 85,71% |
| Xue, 2019 [74] | MA | 1 | 1 | 1 | 1 | 1 | 1 | 1 | 1 | 8 | 8 | 100,00% |
| Tanaka, 2019 [75] | SR | 1 | 1 | 1 | NR | NR | 1 | NR | NA | 4 | 7 | 57,14% |
| Klimova, 2018 [76] | SR | 1 | NR | NR | NR | NR | NR | NR | NA | 1 | 7 | 14,29% |
| Zhang, 2017 [77] | MA | 1 | 1 | 1 | NR | 1 | 1 | 1 | 1 | 7 | 8 | 87,50% |
| Chatterjee, 2016 [78] | MA | 1 | NR | 1 | NR | NR | 1 | 1 | 1 | 5 | 8 | 62,50% |
| Atti, 2019 [79] | MA | 1 | 1 | 1 | 1 | NR | NR | 1 | 1 | 6 | 8 | 75,00% |
| Assuncao, 2018 [80] | SR | 1 | 1 | 1 | 1 | NR | 1 | NR | NA | 5 | 7 | 71,43% |
| Deckers, 2017 [81] | MA | 1 | 1 | 1 | 1 | NR | 1 | 1 | 1 | 7 | 8 | 87,50% |
| Georgakis, 2017 [82] | MA | 1 | 1 | 1 | NR | NR | 1 | 1 | 1 | 6 | 8 | 75,00% |
| Zhuang, 2021 [83] | MA | 1 | 1 | 1 | 1 | NR | 1 | 1 | 1 | 7 | 8 | 87,50% |
| Qu, 2020 [84] | MA | 1 | 1 | 1 | NR | NR | 1 | 1 | 1 | 6 | 8 | 75,00% |
| Danat, 2019 [85] | MA | 1 | 1 | 1 | 1 | NR | 1 | 1 | 1 | 7 | 8 | 87,50% |
| Pedditzi, 2016 [86] | MA | 1 | 1 | 1 | 1 | NR | 1 | NR | NR | 5 | 8 | 62,50% |
| Wang, 2020 [87] | MA | 1 | 1 | 1 | NR | NR | 1 | 1 | 1 | 6 | 8 | 75,00% |
| Nadim, 2020 [88] | MA | 1 | 1 | 1 | 1 | 1 | 1 | 1 | NR | 7 | 8 | 87,50% |
| Lauritano, 2019 [89] | SR | 1 | 1 | 1 | 1 | NR | 1 | NR | NA | 5 | 7 | 71,43% |
| Fang, 2018 [90] | MA | 1 | 1 | 1 | NR | 1 | 1 | 1 | 1 | 7 | 8 | 87,50% |
| Oh, 2018 [91] | MA | 1 | NR | 1 | 1 | NR | 1 | 1 | 1 | 6 | 8 | 75,00% |
| Chen, 2018 [92] | SR | 1 | 1 | 1 | 1 | NR | 1 | 1 | NA | 6 | 7 | 85,71% |
| Tonsekar, 2017 [93] | SR | 1 | 1 | 1 | 1 | 1 | 1 | NR | NA | 6 | 7 | 85,71% |
| Yan, 2016 [94] | MA | 1 | 1 | 1 | NR | 1 | 1 | 1 | 1 | 7 | 8 | 87,50% |
| Della Gatta, 2021 [95] | SR | 1 | 1 | 1 | 1 | 1 | 1 | NR | NA | 6 | 7 | 85,71% |
| Lee, 2018 [96] | MA | 1 | 1 | 1 | 1 | NR | 1 | 1 | 1 | 7 | 8 | 87,50% |
| Xu, 2017 [97] | MA | 1 | 1 | 1 | 1 | NR | 1 | 1 | 1 | 7 | 8 | 87,50% |
| Stephen, 2017 [98] | SR | 1 | 1 | 1 | 1 | NR | 1 | NR | NA | 5 | 7 | 71,43% |
| Santos-Lozano, 2016 [99] | MA | 1 | 1 | 1 | NR | 1 | 1 | 1 | 1 | 7 | 8 | 87,50% |
| Lu, 2016 [100] | SR | 1 | 1 | 1 | NR | NR | 1 | NR | NA | 4 | 7 | 57,14% |
| Santabarbara, 2020 [101] | MA | 1 | 1 | 1 | 1 | 1 | 1 | 1 | 1 | 8 | 8 | 100,00% |
| Santabarbara, 2020 [102] | MA | 1 | 1 | 1 | 1 | 1 | 1 | 1 | 1 | 8 | 8 | 100,00% |
| Santabarbara, 2020 [103] | MA | 1 | 1 | NR | NR | NR | 1 | 1 | 1 | 5 | 8 | 62,50% |
| Santabarbara, 2019 [104] | MA | 1 | 1 | 1 | 1 | 1 | 1 | 1 | 1 | 8 | 8 | 100,00% |
| Becker, 2018 [105] | MA | 1 | 1 | 1 | 1 | NR | 1 | 1 | 1 | 7 | 8 | 87,50% |
| Gimson, 2018 [106] | SR | 1 | 1 | 1 | 1 | 1 | 1 | NR | NA | 6 | 7 | 85,71% |
| Gulpers, 2016 [107] | MA | 1 | 1 | 1 | NR | 1 | 1 | 1 | 1 | 7 | 8 | 87,50% |
| Saiz-Vazquez, 2021 [108] | MA | 1 | 1 | 1 | NR | NR | 1 | 1 | 1 | 6 | 8 | 75,00% |
| Wiels, 2020 [109] | SR | 1 | 1 | 1 | 1 | NR | 1 | NR | NA | 5 | 7 | 71,43% |
| Santabarbara, 2020 [110] | MA | 1 | 1 | 1 | NR | NR | 1 | 1 | 1 | 6 | 8 | 75,00% |
| Chan, 2019 [111] | MA | 1 | 1 | 1 | 1 | NR | 1 | NR | 1 | 6 | 8 | 75,00% |
| Cherbuin, 2015 [112] | MA | 1 | 1 | 1 | 1 | 1 | 1 | 1 | 1 | 8 | 8 | 100,00% |
| Kuring, 2020 [113] | MA | 1 | 1 | 1 | 0 | 0 | 1 | 1 | 1 | 6 | 8 | 75,00% |
| Cruz Freire, 2019 [114] | SR | 1 | 1 | 1 | NR | NR | NR | NR | NA | 3 | 7 | 42,86% |
| Stuart, 2020 [115] | SR | 1 | 1 | 1 | 1 | NR | 1 | NR | NA | 5 | 7 | 71,43% |
| Olanrewaju, 2020 [116] | SR | 1 | 1 | 1 | 1 | 1 | 1 | NR | NA | 6 | 7 | 85,71% |
| Yan, 2020 [117] | MA | 1 | 1 | 1 | NR | 1 | 1 | 1 | 1 | 7 | 8 | 87,50% |
| Falck, 2017 [118] | SR | 1 | 1 | 1 | 1 | 1 | 1 | NR | NA | 6 | 7 | 85,71% |
| Ford, 2018 [119] | MA | 1 | 1 | 1 | NR | NR | 1 | NR | 1 | 5 | 8 | 62,50% |
| Yuan, 2018 [120] | MA | 1 | 1 | 1 | 1 | 1 | 1 | NA | 1 | 7 | 8 | 87,50% |
| Loughrey, 2018 [121] | MA | 1 | 1 | 1 | 1 | 1 | 1 | NR | 1 | 7 | 8 | 87,50% |
| Thomson, 2017 [122] | SR | 1 | 1 | 1 | NR | NR | 1 | NR | NA | 4 | 7 | 57,14% |
| Zheng, 2017 [123] | MA | 1 | 1 | 1 | 1 | NR | 1 | 1 | 1 | 7 | 8 | 87,50% |
| Chen, 2021 [124] | MA | 1 | NR | 1 | 1 | 1 | 1 | 1 | 1 | 7 | 8 | 87,50% |
| Windon, 2020 [125] | SR | 1 | 1 | 1 | 1 | NR | 1 | 1 | NA | 6 | 7 | 85,71% |
| Shang, 2021 [126] | MA | 1 | 1 | 1 | 1 | NR | 1 | 1 | 1 | 7 | 8 | 87,50% |
| Vu, 2021 [127] | MA | 1 | 1 | 1 | 1 | 1 | 1 | 1 | 1 | 8 | 8 | 100,00% |
| Fan, 2019 [128] | MA | 1 | 1 | 1 | 1 | 1 | 1 | 1 | 1 | 8 | 8 | 100,00% |
| Liang, 2019 [129] | MA | 1 | 1 | 1 | 1 | 1 | 1 | NA | 1 | 7 | 8 | 87,50% |
| Wu, 2018 [130] | MA | 1 | 1 | 1 | 1 | NR | 1 | 1 | 1 | 7 | 8 | 87,50% |
| Kim, 2016 [131] | MA | 1 | 1 | 1 | 1 | NR | 1 | 1 | 1 | 7 | 8 | 87,50% |
| Lo, 2018 [132] | MA | 1 | 1 | 1 | NR | NR | 1 | 1 | 1 | 6 | 8 | 75,00% |
| Devore, 2018 [133] | SR | 1 | 1 | 1 | 1 | NR | 1 | NR | NA | 5 | 7 | 71,43% |
| Shi, 2018 [134] | MA | 1 | 1 | 1 | 1 | 1 | 1 | 1 | 1 | 8 | 8 | 100,00% |
| Bubu, 2017 [135] | MA | 1 | 1 | 1 | 1 | NR | 1 | 1 | 1 | 7 | 8 | 87,50% |
| Kitamura, 2020 [136] | SR | 1 | 1 | 1 | NR | NR | 1 | NR | NA | 4 | 7 | 57,14% |
| de Almondes, 2016 [137] | MA | 1 | 1 | 1 | 1 | NR | 1 | 1 | 1 | 7 | 8 | 87,50% |
| Zhu, 2018 [138] | MA | 1 | 1 | 1 | NR | NR | 1 | 1 | 1 | 6 | 8 | 75,00% |
| Leng, 2017 [139] | MA | 1 | 1 | 1 | 1 | 1 | 1 | 1 | 1 | 8 | 8 | 100,00% |
| Lam, 2017 [140] | SR | 1 | 1 | 1 | 1 | 1 | 1 | NR | NA | 6 | 7 | 85,71% |
| Niu, 2018 [141] | MA | 1 | 1 | 1 | 1 | NR | 1 | 1 | 1 | 7 | 8 | 87,50% |
| Stirland, 2018 [142] | SR | 1 | 1 | 1 | 1 | 1 | 1 | NR | NA | 6 | 7 | 85,71% |
| Desai, 2020 [143] | MA | 1 | 1 | 1 | NR | NR | 1 | 1 | 1 | 6 | 8 | 75,00% |
| Lara, 2019 [144] | MA | 1 | 1 | 1 | 1 | NR | 1 | 1 | 1 | 7 | 8 | 87,50% |
| Hosseini, 2019 [145] | SR | 1 | 1 | 1 | 1 | NR | 1 | NA | NA | 5 | 7 | 71,43% |
| Kuiper, 2016 [146] | MA | 1 | 1 | 1 | 1 | 1 | 1 | 1 | 1 | 8 | 8 | 100,00% |
| Kuiper, 2015 [147] | MA | 1 | 1 | 1 | 1 | 1 | 1 | 1 | 1 | 8 | 8 | 100,00% |
| Boss, 2015 [148] | SR | 1 | 1 | 1 | NR | NR | 1 | NR | NA | 4 | 7 | 57,14% |

NOTE. The quality of included SR/MA was assessed using the 7/8-item National Institute of Health (NIH) Quality Assessment Tool of Systematic Reviews and Meta-analyses. Depending on whether the study was a systematic review or meta-analysis, it could be scored on seven or eight criteria, respectively. Total % scores were calculated as the total score for a study divided by the maximum possible score (seven or eight).
Abbreviations: MA, Meta-analysis; NA, Not applicable; NR, Not reported; SR, Systematic review

**References**

[1] Lao Y, Hou L, Li J, Hui X, Yan P, Yang K. Association between alcohol intake, mild cognitive impairment and progression to dementia: a dose-response meta-analysis. Aging Clin Exp Res. 2021;33:1175-85.

[2] Wiegmann C, Mick I, Brandl EJ, Heinz A, Gutwinski S. Alcohol and Dementia - What is the Link? A Systematic Review. Neuropsychiatr Dis Treat. 2020;16:87-99.

[3] Xu W, Wang H, Wan Y, Tan C, Li J, Tan L, et al. Alcohol consumption and dementia risk: a dose-response meta-analysis of prospective studies. Eur J Epidemiol. 2017;32:31-42.

[4] Forte G, Casagrande M. Effects of Blood Pressure on Cognitive Performance in Aging: A Systematic Review. Brain Sci. 2020;10.

[5] Ou YN, Tan CC, Shen XN, Xu W, Hou XH, Dong Q, et al. Blood Pressure and Risks of Cognitive Impairment and Dementia: A Systematic Review and Meta-Analysis of 209 Prospective Studies. Hypertension. 2020;76:217-25.

[6] Min M, Shi T, Sun C, Liang M, Zhang Y, Tian S, et al. The association between orthostatic hypotension and cognition and stroke: a meta-analysis of prospective cohort studies. Blood Press. 2020;29:3-12.

[7] Lennon MJ, Makkar SR, Crawford JD, Sachdev PS. Midlife Hypertension and Alzheimer's Disease: A Systematic Review and Meta-Analysis. J Alzheimers Dis. 2019;71:307-16.

[8] Iseli R, Nguyen VTV, Sharmin S, Reijnierse EM, Lim WK, Maier AB. Orthostatic hypotension and cognition in older adults: A systematic review and meta-analysis. Exp Gerontol. 2019;120:40-9.

[9] Agarwal M, Khan S. Plasma Lipids as Biomarkers for Alzheimer's Disease: A Systematic Review. Cureus. 2020;12:e12008.

[10] Anstey KJ, Ashby-Mitchell K, Peters R. Updating the Evidence on the Association between Serum Cholesterol and Risk of Late-Life Dementia: Review and Meta-Analysis. J Alzheimers Dis. 2017;56:215-28.

[11] Koch M, Jensen MK. HDL-cholesterol and apolipoproteins in relation to dementia. Curr Opin Lipidol. 2016;27:76-87.

[12] Walsh S, Causer R, Brayne C. Does playing a musical instrument reduce the incidence of cognitive impairment and dementia? A systematic review and meta-analysis. Aging Ment Health. 2021;25:593-601.

[13] Anderson JAE, Hawrylewicz K, Grundy JG. Does bilingualism protect against dementia? A meta-analysis. Psychon Bull Rev. 2020;27:952-65.

[14] Brini S, Sohrabi HR, Hebert JJ, Forrest MRL, Laine M, Hämäläinen H, et al. Bilingualism Is Associated with a Delayed Onset of Dementia but Not with a Lower Risk of Developing it: a Systematic Review with Meta-Analyses. Neuropsychol Rev. 2020;30:1-24.

[15] Mukadam N, Sommerlad A, Livingston G. The Relationship of Bilingualism Compared to Monolingualism to the Risk of Cognitive Decline or Dementia: A Systematic Review and Meta-Analysis. J Alzheimers Dis. 2017;58:45-54.

[16] Yates LA, Ziser S, Spector A, Orrell M. Cognitive leisure activities and future risk of cognitive impairment and dementia: systematic review and meta-analysis. Int Psychogeriatr. 2016;28:1791-806.

[17] Sajeev G, Weuve J, Jackson JW, VanderWeele TJ, Bennett DA, Grodstein F, et al. Late-life Cognitive Activity and Dementia: A Systematic Review and Bias Analysis. Epidemiology. 2016;27:732-42.

[18] Yu JT, Xu W, Tan CC, Andrieu S, Suckling J, Evangelou E, et al. Evidence-based prevention of Alzheimer's disease: systematic review and meta-analysis of 243 observational prospective studies and 153 randomised controlled trials. J Neurol Neurosurg Psychiatry. 2020;91:1201-9.

[19] Hudon C, Escudier F, De Roy J, Croteau J, Cross N, Dang-Vu TT, et al. Behavioral and Psychological Symptoms that Predict Cognitive Decline or Impairment in Cognitively Normal Middle-Aged or Older Adults: a Meta-Analysis. Neuropsychol Rev. 2020;30:558-79.

[20] Olayinka O, Olayinka O, Alemu B, Akpinar-Elci M, Grossberg G. Toxic Environmental Risk Factors for Alzheimer’s Disease: A Systematic Review. Aging Medicine and Healthcare. 2019;10:4-17.

[21] Hersi M, Irvine B, Gupta P, Gomes J, Birkett N, Krewski D. Risk factors associated with the onset and progression of Alzheimer's disease: A systematic review of the evidence. Neurotoxicology. 2017;61:143-87.

[22] Lafortune L, Martin S, Kelly S, Kuhn I, Remes O, Cowan A, et al. Behavioural Risk Factors in Mid-Life Associated with Successful Ageing, Disability, Dementia and Frailty in Later Life: A Rapid Systematic Review. PLoS One. 2016;11:e0144405.

[23] Xu W, Tan L, Wang HF, Jiang T, Tan MS, Tan L, et al. Meta-analysis of modifiable risk factors for Alzheimer's disease. J Neurol Neurosurg Psychiatry. 2015;86:1299-306.

[24] Proietti R, AlTurki A, Vio R, Licchelli L, Rivezzi F, Marafi M, et al. The association between atrial fibrillation and Alzheimer's disease: fact or fallacy? A systematic review and meta-analysis. J Cardiovasc Med (Hagerstown). 2020;21:106-12.

[25] Liu DS, Chen J, Jian WM, Zhang GR, Liu ZR. The association of atrial fibrillation and dementia incidence: a meta-analysis of prospective cohort studies. J Geriatr Cardiol. 2019;16:298-306.

[26] Islam MM, Poly TN, Walther BA, Yang HC, Wu CC, Lin MC, et al. Association Between Atrial Fibrillation and Dementia: A Meta-Analysis. Front Aging Neurosci. 2019;11:305.

[27] Koh YH, Lew LZW, Franke KB, Elliott AD, Lau DH, Thiyagarajah A, et al. Predictive role of atrial fibrillation in cognitive decline: a systematic review and meta-analysis of 2.8 million individuals. Europace. 2022;24:1229-39.

[28] Liang X, Huang Y, Han X. Associations between coronary heart disease and risk of cognitive impairment: A meta-analysis. Brain Behav. 2021;11:e02108.

[29] Xia C, Vonder M, Sidorenkov G, Oudkerk M, de Groot JC, van der Harst P, et al. The Relationship of Coronary Artery Calcium and Clinical Coronary Artery Disease with Cognitive Function: A Systematic Review and Meta-Analysis. J Atheroscler Thromb. 2020;27:934-58.

[30] Wolters FJ, Segufa RA, Darweesh SKL, Bos D, Ikram MA, Sabayan B, et al. Coronary heart disease, heart failure, and the risk of dementia: A systematic review and meta-analysis. Alzheimers Dement. 2018;14:1493-504.

[31] Deckers K, Schievink SHJ, Rodriquez MMF, van Oostenbrugge RJ, van Boxtel MPJ, Verhey FRJ, et al. Coronary heart disease and risk for cognitive impairment or dementia: Systematic review and meta-analysis. PLoS One. 2017;12:e0184244.

[32] Stefanidis KB, Askew CD, Greaves K, Summers MJ. The Effect of Non-Stroke Cardiovascular Disease States on Risk for Cognitive Decline and Dementia: A Systematic and Meta-Analytic Review. Neuropsychol Rev. 2018;28:1-15.

[33] Li J, Wu Y, Zhang D, Nie J. Associations between heart failure and risk of dementia: A PRISMA-compliant meta-analysis. Medicine (Baltimore). 2020;99:e18492.

[34] Cannon JA, Moffitt P, Perez-Moreno AC, Walters MR, Broomfield NM, McMurray JJV, et al. Cognitive Impairment and Heart Failure: Systematic Review and Meta-Analysis. J Card Fail. 2017;23:464-75.

[35] Chen JQA, Scheltens P, Groot C, Ossenkoppele R. Associations Between Caffeine Consumption, Cognitive Decline, and Dementia: A Systematic Review. J Alzheimers Dis. 2020;78:1519-46.

[36] Larsson SC, Orsini N. Coffee Consumption and Risk of Dementia and Alzheimer's Disease: A Dose-Response Meta-Analysis of Prospective Studies. Nutrients. 2018;10.

[37] Wu L, Sun D, He Y. Coffee intake and the incident risk of cognitive disorders: A dose-response meta-analysis of nine prospective cohort studies. Clin Nutr. 2017;36:730-6.

[38] Liu QP, Wu YF, Cheng HY, Xia T, Ding H, Wang H, et al. Habitual coffee consumption and risk of cognitive decline/dementia: A systematic review and meta-analysis of prospective cohort studies. Nutrition. 2016;32:628-36.

[39] Cuesta-Triana F, Verdejo-Bravo C, Fernández-Pérez C, Martín-Sánchez FJ. Effect of Milk and Other Dairy Products on the Risk of Frailty, Sarcopenia, and Cognitive Performance Decline in the Elderly: A Systematic Review. Adv Nutr. 2019;10:S105-s19.

[40] Lee J, Fu Z, Chung M, Jang DJ, Lee HJ. Role of milk and dairy intake in cognitive function in older adults: a systematic review and meta-analysis. Nutr J. 2018;17:82.

[41] Wu L, Sun D. Meta-Analysis of Milk Consumption and the Risk of Cognitive Disorders. Nutrients. 2016;8.

[42] Bakre AT, Chen R, Khutan R, Wei L, Smith T, Qin G, et al. Association between fish consumption and risk of dementia: a new study from China and a systematic literature review and meta-analysis. Public Health Nutr. 2018;21:1921-32.

[43] Zeng LF, Cao Y, Liang WX, Bao WH, Pan JK, Wang Q, et al. An exploration of the role of a fish-oriented diet in cognitive decline: a systematic review of the literature. Oncotarget. 2017;8:39877-95.

[44] Zhang X, Bao G, Liu D, Yang Y, Li X, Cai G, et al. The Association Between Folate and Alzheimer's Disease: A Systematic Review and Meta-Analysis. Front Neurosci. 2021;15:661198.

[45] Mottaghi T, Amirabdollahian F, Haghighatdoost F. Fruit and vegetable intake and cognitive impairment: a systematic review and meta-analysis of observational studies. Eur J Clin Nutr. 2018;72:1336-44.

[46] Wu L, Sun D, Tan Y. Intake of Fruit and Vegetables and the Incident Risk of Cognitive Disorders: A Systematic Review and Meta-Analysis of Cohort Studies. J Nutr Health Aging. 2017;21:1284-90.

[47] Jiang X, Huang J, Song D, Deng R, Wei J, Zhang Z. Increased Consumption of Fruit and Vegetables Is Related to a Reduced Risk of Cognitive Impairment and Dementia: Meta-Analysis. Front Aging Neurosci. 2017;9:18.

[48] Ran LS, Liu WH, Fang YY, Xu SB, Li J, Luo X, et al. Alcohol, coffee and tea intake and the risk of cognitive deficits: a dose-response meta-analysis. Epidemiol Psychiatr Sci. 2021;30:e13.

[49] Moradi S, Moloudi J, Moradinazar M, Sarokhani D, Nachvak SM, Samadi M. Adherence to Healthy Diet Can Delay Alzheimer's Diseases Development: A Systematic Review and Meta-Analysis. Prev Nutr Food Sci. 2020;25:325-37.

[50] Vicente BM, Lucio Dos Santos Quaresma MV, Maria de Melo C, Lima Ribeiro SM. The dietary inflammatory index (DII®) and its association with cognition, frailty, and risk of disabilities in older adults: A systematic review. Clin Nutr ESPEN. 2020;40:7-16.

[51] Liu YH, Gao X, Na M, Kris-Etherton PM, Mitchell DC, Jensen GL. Dietary Pattern, Diet Quality, and Dementia: A Systematic Review and Meta-Analysis of Prospective Cohort Studies. J Alzheimers Dis. 2020;78:151-68.

[52] Kheirouri S, Alizadeh M. Dietary Inflammatory Potential and the Risk of Neurodegenerative Diseases in Adults. Epidemiol Rev. 2019;41:109-20.

[53] Samadi M, Moradi S, Moradinazar M, Mostafai R, Pasdar Y. Dietary pattern in relation to the risk of Alzheimer's disease: a systematic review. Neurol Sci. 2019;40:2031-43.

[54] Nowson CA, Service C, Appleton J, Grieger JA. The Impact of Dietary Factors on Indices of Chronic Disease in Older People: A Systematic Review. J Nutr Health Aging. 2018;22:282-96.

[55] Solfrizzi V, Custodero C, Lozupone M, Imbimbo BP, Valiani V, Agosti P, et al. Relationships of Dietary Patterns, Foods, and Micro- and Macronutrients with Alzheimer's Disease and Late-Life Cognitive Disorders: A Systematic Review. J Alzheimers Dis. 2017;59:815-49.

[56] Yusufov M, Weyandt LL, Piryatinsky I. Alzheimer's disease and diet: a systematic review. Int J Neurosci. 2017;127:161-75.

[57] Masana MF, Koyanagi A, Haro JM, Tyrovolas S. n-3 Fatty acids, Mediterranean diet and cognitive function in normal aging: A systematic review. Exp Gerontol. 2017;91:39-50.

[58] Cao L, Tan L, Wang HF, Jiang T, Zhu XC, Lu H, et al. Dietary Patterns and Risk of Dementia: a Systematic Review and Meta-Analysis of Cohort Studies. Mol Neurobiol. 2016;53:6144-54.

[59] van de Rest O, Berendsen AA, Haveman-Nies A, de Groot LC. Dietary patterns, cognitive decline, and dementia: a systematic review. Adv Nutr. 2015;6:154-68.

[60] Panza F, Solfrizzi V, Barulli MR, Bonfiglio C, Guerra V, Osella A, et al. Coffee, tea, and caffeine consumption and prevention of late-life cognitive decline and dementia: a systematic review. J Nutr Health Aging. 2015;19:313-28.

[61] Kim YS, Kwak SM, Myung SK. Caffeine intake from coffee or tea and cognitive disorders: a meta-analysis of observational studies. Neuroepidemiology. 2015;44:51-63.

[62] Cao GY, Li M, Han L, Tayie F, Yao SS, Huang Z, et al. Dietary Fat Intake and Cognitive Function among Older Populations: A Systematic Review and Meta-Analysis. J Prev Alzheimers Dis. 2019;6:204-11.

[63] Zhang H, Hardie L, Bawajeeh AO, Cade J. Meat Consumption, Cognitive Function and Disorders: A Systematic Review with Narrative Synthesis and Meta-Analysis. Nutrients. 2020;12.

[64] Limongi F, Siviero P, Bozanic A, Noale M, Veronese N, Maggi S. The Effect of Adherence to the Mediterranean Diet on Late-Life Cognitive Disorders: A Systematic Review. J Am Med Dir Assoc. 2020;21:1402-9.

[65] Aridi YS, Walker JL, Wright ORL. The Association between the Mediterranean Dietary Pattern and Cognitive Health: A Systematic Review. Nutrients. 2017;9.

[66] Wu L, Sun D. Adherence to Mediterranean diet and risk of developing cognitive disorders: An updated systematic review and meta-analysis of prospective cohort studies. Sci Rep. 2017;7:41317.

[67] Petersson SD, Philippou E. Mediterranean Diet, Cognitive Function, and Dementia: A Systematic Review of the Evidence. Adv Nutr. 2016;7:889-904.

[68] Theodore LE, Kellow NJ, McNeil EA, Close EO, Coad EG, Cardoso BR. Nut Consumption for Cognitive Performance: A Systematic Review. Adv Nutr. 2021;12:777-92.

[69] Wu S, Ding Y, Wu F, Li R, Hou J, Mao P. Omega-3 fatty acids intake and risks of dementia and Alzheimer's disease: a meta-analysis. Neurosci Biobehav Rev. 2015;48:1-9.

[70] Kakutani S, Watanabe H, Murayama N. Green Tea Intake and Risks for Dementia, Alzheimer's Disease, Mild Cognitive Impairment, and Cognitive Impairment: A Systematic Review. Nutrients. 2019;11.

[71] Liu X, Du X, Han G, Gao W. Association between tea consumption and risk of cognitive disorders: A dose-response meta-analysis of observational studies. Oncotarget. 2017;8:43306-21.

[72] Ma QP, Huang C, Cui QY, Yang DJ, Sun K, Chen X, et al. Meta-Analysis of the Association between Tea Intake and the Risk of Cognitive Disorders. PLoS One. 2016;11:e0165861.

[73] Papunen S, Mustakallio-Könönen A, Auvinen J, Timonen M, Keinänen-Kiukaanniemi S, Sebert S. The association between diabetes and cognitive changes during aging. Scand J Prim Health Care. 2020;38:281-90.

[74] Xue M, Xu W, Ou YN, Cao XP, Tan MS, Tan L, et al. Diabetes mellitus and risks of cognitive impairment and dementia: A systematic review and meta-analysis of 144 prospective studies. Ageing Res Rev. 2019;55:100944.

[75] Tanaka H, Ihana-Sugiyama N, Sugiyama T, Ohsugi M. Contribution of Diabetes to the Incidence and Prevalence of Comorbid Conditions (Cancer, Periodontal Disease, Fracture, Impaired Cognitive Function, and Depression): A Systematic Review of Epidemiological Studies in Japanese Populations. J Epidemiol. 2019;29:1-10.

[76] Klimova B, Kuca K, Maresova P. Global View on Alzheimer's Disease and Diabetes Mellitus: Threats, Risks and Treatment Alzheimer's Disease and Diabetes Mellitus. Curr Alzheimer Res. 2018;15:1277-82.

[77] Zhang J, Chen C, Hua S, Liao H, Wang M, Xiong Y, et al. An updated meta-analysis of cohort studies: Diabetes and risk of Alzheimer's disease. Diabetes Res Clin Pract. 2017;124:41-7.

[78] Chatterjee S, Peters SA, Woodward M, Mejia Arango S, Batty GD, Beckett N, et al. Type 2 Diabetes as a Risk Factor for Dementia in Women Compared With Men: A Pooled Analysis of 2.3 Million People Comprising More Than 100,000 Cases of Dementia. Diabetes Care. 2016;39:300-7.

[79] Atti AR, Valente S, Iodice A, Caramella I, Ferrari B, Albert U, et al. Metabolic Syndrome, Mild Cognitive Impairment, and Dementia: A Meta-Analysis of Longitudinal Studies. Am J Geriatr Psychiatry. 2019;27:625-37.

[80] Assuncao N, Sudo FK, Drummond C, de Felice FG, Mattos P. Metabolic Syndrome and cognitive decline in the elderly: A systematic review. PLoS One. 2018;13:e0194990.

[81] Deckers K, Camerino I, van Boxtel MP, Verhey FR, Irving K, Brayne C, et al. Dementia risk in renal dysfunction: A systematic review and meta-analysis of prospective studies. Neurology. 2017;88:198-208.

[82] Georgakis MK, Dimitriou NG, Karalexi MA, Mihas C, Nasothimiou EG, Tousoulis D, et al. Albuminuria in Association with Cognitive Function and Dementia: A Systematic Review and Meta-Analysis. J Am Geriatr Soc. 2017;65:1190-8.

[83] Zhuang QS, Meng L, Wang Z, Shen L, Ji HF. Associations Between Obesity and Alzheimer's Disease: Multiple Bioinformatic Analyses. J Alzheimers Dis. 2021;80:271-81.

[84] Qu Y, Hu HY, Ou YN, Shen XN, Xu W, Wang ZT, et al. Association of body mass index with risk of cognitive impairment and dementia: A systematic review and meta-analysis of prospective studies. Neurosci Biobehav Rev. 2020;115:189-98.

[85] Danat IM, Clifford A, Partridge M, Zhou W, Bakre AT, Chen A, et al. Impacts of Overweight and Obesity in Older Age on the Risk of Dementia: A Systematic Literature Review and a Meta-Analysis. J Alzheimers Dis. 2019;70:S87-s99.

[86] Pedditzi E, Peters R, Beckett N. The risk of overweight/obesity in mid-life and late life for the development of dementia: a systematic review and meta-analysis of longitudinal studies. Age Ageing. 2016;45:14-21.

[87] Wang T, Zhang Q, Liu X, Ma L, Fu J, Gao Y, et al. Periodontal disease and cognitive deficits: A systematic review and meta-analysis. Neurology Asia. 2020;25:341-52.

[88] Nadim R, Tang J, Dilmohamed A, Yuan S, Wu C, Bakre AT, et al. Influence of periodontal disease on risk of dementia: a systematic literature review and a meta-analysis. Eur J Epidemiol. 2020;35:821-33.

[89] Lauritano D, Moreo G, Della Vella F, Di Stasio D, Carinci F, Lucchese A, et al. Oral Health Status and Need for Oral Care in an Aging Population: A Systematic Review. Int J Environ Res Public Health. 2019;16.

[90] Fang WL, Jiang MJ, Gu BB, Wei YM, Fan SN, Liao W, et al. Tooth loss as a risk factor for dementia: systematic review and meta-analysis of 21 observational studies. BMC Psychiatry. 2018;18:345.

[91] Oh B, Han DH, Han KT, Liu X, Ukken J, Chang C, et al. Association between residual teeth number in later life and incidence of dementia: A systematic review and meta-analysis. BMC Geriatr. 2018;18:48.

[92] Chen J, Ren CJ, Wu L, Xia LY, Shao J, Leng WD, et al. Tooth Loss Is Associated With Increased Risk of Dementia and With a Dose-Response Relationship. Front Aging Neurosci. 2018;10:415.

[93] Tonsekar PP, Jiang SS, Yue G. Periodontal disease, tooth loss and dementia: Is there a link? A systematic review. Gerodontology. 2017;34:151-63.

[94] Yan D, Zhang Y, Liu L, Yan H. Pesticide exposure and risk of Alzheimer's disease: a systematic review and meta-analysis. Sci Rep. 2016;6:32222.

[95] Della Gatta F, Lacorte E, Fabrizi E, Remoli G, Cipollini V, Troili F, et al. Exploring the association of early life physical activity and risk of dementia: a systematic review. Minerva Med. 2021;112:448-55.

[96] Lee J. The Relationship Between Physical Activity and Dementia: A Systematic Review and Meta-Analysis of Prospective Cohort Studies. J Gerontol Nurs. 2018;44:22-9.

[97] Xu W, Wang HF, Wan Y, Tan CC, Yu JT, Tan L. Leisure time physical activity and dementia risk: a dose-response meta-analysis of prospective studies. BMJ Open. 2017;7:e014706.

[98] Stephen R, Hongisto K, Solomon A, Lönnroos E. Physical Activity and Alzheimer's Disease: A Systematic Review. J Gerontol A Biol Sci Med Sci. 2017;72:733-9.

[99] Santos-Lozano A, Pareja-Galeano H, Sanchis-Gomar F, Quindós-Rubial M, Fiuza-Luces C, Cristi-Montero C, et al. Physical Activity and Alzheimer Disease: A Protective Association. Mayo Clin Proc. 2016;91:999-1020.

[100] Lü J, Fu W, Liu Y. Physical activity and cognitive function among older adults in China: A systematic review. J Sport Health Sci. 2016;5:287-96.

[101] Santabárbara J, Lipnicki DM, Olaya B, Villagrasa B, Gracia-García P, Bueno-Notivol J, et al. Association between Anxiety and Vascular Dementia Risk: New Evidence and an Updated Meta-Analysis. J Clin Med. 2020;9.

[102] Santabárbara J, Lipnicki DM, Bueno-Notivol J, Olaya-Guzmán B, Villagrasa B, López-Antón R. Updating the evidence for an association between anxiety and risk of Alzheimer's disease: A meta-analysis of prospective cohort studies. J Affect Disord. 2020;262:397-404.

[103] Santabárbara J, Lipnicki DM, Olaya B, Villagrasa B, Bueno-Notivol J, Nuez L, et al. Does Anxiety Increase the Risk of All-Cause Dementia? An Updated Meta-Analysis of Prospective Cohort Studies. J Clin Med. 2020;9.

[104] Santabárbara J, Lipnicki DM, Villagrasa B, Lobo E, Lopez-Anton R. Anxiety and risk of dementia: Systematic review and meta-analysis of prospective cohort studies. Maturitas. 2019;119:14-20.

[105] Becker E, Orellana Rios CL, Lahmann C, Rücker G, Bauer J, Boeker M. Anxiety as a risk factor of Alzheimer's disease and vascular dementia. Br J Psychiatry. 2018;213:654-60.

[106] Gimson A, Schlosser M, Huntley JD, Marchant NL. Support for midlife anxiety diagnosis as an independent risk factor for dementia: a systematic review. BMJ Open. 2018;8:e019399.

[107] Gulpers B, Ramakers I, Hamel R, Köhler S, Oude Voshaar R, Verhey F. Anxiety as a Predictor for Cognitive Decline and Dementia: A Systematic Review and Meta-Analysis. Am J Geriatr Psychiatry. 2016;24:823-42.

[108] Sáiz-Vázquez O, Gracia-García P, Ubillos-Landa S, Puente-Martínez A, Casado-Yusta S, Olaya B, et al. Depression as a Risk Factor for Alzheimer's Disease: A Systematic Review of Longitudinal Meta-Analyses. J Clin Med. 2021;10.

[109] Wiels W, Baeken C, Engelborghs S. Depressive Symptoms in the Elderly-An Early Symptom of Dementia? A Systematic Review. Front Pharmacol. 2020;11:34.

[110] Santabárbara J, Villagrasa B, Gracia-García P. Does depression increase the risk of dementia? Updated meta-analysis of prospective studies. Actas Esp Psiquiatr. 2020;48:169-80.

[111] Chan JYC, Yiu KKL, Kwok TCY, Wong SYS, Tsoi KKF. Depression and Antidepressants as Potential Risk Factors in Dementia: A Systematic Review and Meta-analysis of 18 Longitudinal Studies. J Am Med Dir Assoc. 2019;20:279-86.e1.

[112] Cherbuin N, Kim S, Anstey KJ. Dementia risk estimates associated with measures of depression: a systematic review and meta-analysis. BMJ Open. 2015;5:e008853.

[113] Kuring JK, Mathias JL, Ward L. Risk of Dementia in persons who have previously experienced clinically-significant Depression, Anxiety, or PTSD: A Systematic Review and Meta-Analysis. J Affect Disord. 2020;274:247-61.

[114] Cruz Freire A, Couto K, da Silva E, Pondé M. Association between anxiety, depression and cognitive impairment in community-dwelling older adults: a systematic review. Revista Brasileira de Neurologia e Psiquiatria. 2019;23:242-54.

[115] Stuart KE, Padgett C. A Systematic Review of the Association Between Psychological Stress and Dementia Risk in Humans. J Alzheimers Dis. 2020;78:335-52.

[116] Olanrewaju O, Stockwell S, Stubbs B, Smith L. Sedentary behaviours, cognitive function, and possible mechanisms in older adults: a systematic review. Aging Clin Exp Res. 2020;32:969-84.

[117] Yan S, Fu W, Wang C, Mao J, Liu B, Zou L, et al. Association between sedentary behavior and the risk of dementia: a systematic review and meta-analysis. Transl Psychiatry. 2020;10:112.

[118] Falck RS, Davis JC, Liu-Ambrose T. What is the association between sedentary behaviour and cognitive function? A systematic review. Br J Sports Med. 2017;51:800-11.

[119] Ford AH, Hankey GJ, Yeap BB, Golledge J, Flicker L, Almeida OP. Hearing loss and the risk of dementia in later life. Maturitas. 2018;112:1-11.

[120] Yuan J, Sun Y, Sang S, Pham JH, Kong WJ. The risk of cognitive impairment associated with hearing function in older adults: a pooled analysis of data from eleven studies. Sci Rep. 2018;8:2137.

[121] Loughrey DG, Kelly ME, Kelley GA, Brennan S, Lawlor BA. Association of Age-Related Hearing Loss With Cognitive Function, Cognitive Impairment, and Dementia: A Systematic Review and Meta-analysis. JAMA Otolaryngol Head Neck Surg. 2018;144:115-26.

[122] Thomson RS, Auduong P, Miller AT, Gurgel RK. Hearing loss as a risk factor for dementia: A systematic review. Laryngoscope Investig Otolaryngol. 2017;2:69-79.

[123] Zheng Y, Fan S, Liao W, Fang W, Xiao S, Liu J. Hearing impairment and risk of Alzheimer's disease: a meta-analysis of prospective cohort studies. Neurol Sci. 2017;38:233-9.

[124] Chen Z, Xie H, Yao L, Wei Y. Olfactory impairment and the risk of cognitive decline and dementia in older adults: a meta-analysis. Braz J Otorhinolaryngol. 2021;87:94-102.

[125] Windon MJ, Kim SJ, Oh ES, Lin SY. Predictive value of olfactory impairment for cognitive decline among cognitively normal adults. Laryngoscope. 2020;130:840-7.

[126] Shang X, Zhu Z, Wang W, Ha J, He M. The Association between Vision Impairment and Incidence of Dementia and Cognitive Impairment: A Systematic Review and Meta-analysis. Ophthalmology. 2021;128:1135-49.

[127] Vu TA, Fenwick EK, Gan ATL, Man REK, Tan BKJ, Gupta P, et al. The Bidirectional Relationship between Vision and Cognition: A Systematic Review and Meta-analysis. Ophthalmology. 2021;128:981-92.

[128] Fan L, Xu W, Cai Y, Hu Y, Wu C. Sleep Duration and the Risk of Dementia: A Systematic Review and Meta-analysis of Prospective Cohort Studies. J Am Med Dir Assoc. 2019;20:1480-7.e5.

[129] Liang Y, Qu LB, Liu H. Non-linear associations between sleep duration and the risks of mild cognitive impairment/dementia and cognitive decline: a dose-response meta-analysis of observational studies. Aging Clin Exp Res. 2019;31:309-20.

[130] Wu L, Sun D, Tan Y. A systematic review and dose-response meta-analysis of sleep duration and the occurrence of cognitive disorders. Sleep Breath. 2018;22:805-14.

[131] Kim HB, Myung SK, Lee SM, Park YC. Longer Duration of Sleep and Risk of Cognitive Decline: A Meta-Analysis of Observational Studies. Neuroepidemiology. 2016;47:171-80.

[132] Lo JC, Groeger JA, Cheng GH, Dijk DJ, Chee MW. Self-reported sleep duration and cognitive performance in older adults: a systematic review and meta-analysis. Sleep Med. 2016;17:87-98.

[133] Devore EE, Grodstein F, Schernhammer ES. Sleep Duration in Relation to Cognitive Function among Older Adults: A Systematic Review of Observational Studies. Neuroepidemiology. 2016;46:57-78.

[134] Shi L, Chen SJ, Ma MY, Bao YP, Han Y, Wang YM, et al. Sleep disturbances increase the risk of dementia: A systematic review and meta-analysis. Sleep Med Rev. 2018;40:4-16.

[135] Bubu OM, Brannick M, Mortimer J, Umasabor-Bubu O, Sebastião YV, Wen Y, et al. Sleep, Cognitive impairment, and Alzheimer's disease: A Systematic Review and Meta-Analysis. Sleep. 2017;40.

[136] Kitamura T, Miyazaki S, Sulaiman HB, Akaike R, Ito Y, Suzuki H. Insomnia and obstructive sleep apnea as potential triggers of dementia: is personalized prediction and prevention of the pathological cascade applicable? Epma j. 2020;11:355-65.

[137] de Almondes KM, Costa MV, Malloy-Diniz LF, Diniz BS. Insomnia and risk of dementia in older adults: Systematic review and meta-analysis. J Psychiatr Res. 2016;77:109-15.

[138] Zhu X, Zhao Y. Sleep-disordered breathing and the risk of cognitive decline: a meta-analysis of 19,940 participants. Sleep Breath. 2018;22:165-73.

[139] Leng Y, McEvoy CT, Allen IE, Yaffe K. Association of Sleep-Disordered Breathing With Cognitive Function and Risk of Cognitive Impairment: A Systematic Review and Meta-analysis. JAMA Neurol. 2017;74:1237-45.

[140] Lam EWK, Chung F, Wong J. Sleep-Disordered Breathing, Postoperative Delirium, and Cognitive Impairment. Anesth Analg. 2017;124:1626-35.

[141] Niu H, Qu Y, Li Z, Wang R, Li L, Li M, et al. Smoking and Risk for Alzheimer Disease: A Meta-Analysis Based on Both Case-Control and Cohort Study. J Nerv Ment Dis. 2018;206:680-5.

[142] Stirland LE, O'Shea CI, Russ TC. Passive smoking as a risk factor for dementia and cognitive impairment: systematic review of observational studies. Int Psychogeriatr. 2018;30:1177-87.

[143] Desai R, John A, Stott J, Charlesworth G. Living alone and risk of dementia: A systematic review and meta-analysis. Ageing Res Rev. 2020;62:101122.

[144] Lara E, Martín-María N, De la Torre-Luque A, Koyanagi A, Vancampfort D, Izquierdo A, et al. Does loneliness contribute to mild cognitive impairment and dementia? A systematic review and meta-analysis of longitudinal studies. Ageing Res Rev. 2019;52:7-16.

[145] Hosseini S, Chaurasia A, Oremus M. The Effect of Religion and Spirituality on Cognitive Function: A Systematic Review. Gerontologist. 2019;59:e76-e85.

[146] Kuiper JS, Zuidersma M, Zuidema SU, Burgerhof JG, Stolk RP, Oude Voshaar RC, et al. Social relationships and cognitive decline: a systematic review and meta-analysis of longitudinal cohort studies. Int J Epidemiol. 2016;45:1169-206.

[147] Kuiper JS, Zuidersma M, Oude Voshaar RC, Zuidema SU, van den Heuvel ER, Stolk RP, et al. Social relationships and risk of dementia: A systematic review and meta-analysis of longitudinal cohort studies. Ageing Res Rev. 2015;22:39-57.

[148] Boss L, Kang DH, Branson S. Loneliness and cognitive function in the older adult: a systematic review. Int Psychogeriatr. 2015;27:541-53.
