# Supplementary material for: Umbrella review and Delphi study on modifiable factors for dementia risk reduction
Source: Alzheimers Dement. 2023 Dec 30;20(3):2223–39. doi: 10.1002/alz.13577 (PMC10984497; doi:10.1002/alz.13577)
Supplement: Supplementary file 6 — Supporting Information [file ALZ-20-2223-s009.docx]

**Appendix F: Rank Score Calculation**
In the first Delphi round, the 18 participating experts were asked to freely name new modifiable risk and protective factors which are not currently included in the LIBRA index. To readily identify the most important factors according to the experts, we asked them to give their answers in subjective order of importance. Subsequently, their responses were given a “rank score (RS)” by using the following exponential scale:

- rank 1 = 100 points (10^2^)
- rank 2 = 81 points (9^2^)
- rank 3 = 64 points (8^2^)
- rank 4 = 49 points (7^2^)
- rank 5 = 36 points (6^2^)
- rank 6 = 25 points (5^2^)
- rank 7 = 16 points (4^2^)
- rank 8 = 9 points (3^2^)
- rank 9 = 4 points (2^2^)
- rank 10 = 2 points (1^2^)

Ultimately, the RS for each factor was summed among the different experts to come to a final total RS

**Example**As illustrated in Table 4 of the main text, “Atrial fibrillation” was mentioned three times by the Delphi experts and was given the following ranks: 4, 6, and 8. This results in the following rank scores: rank 4 = 49 points (7^2); rank 6 = 25 points (5^2); rank 8 = 9 points (3^2). The total rank score for “Atrial fibrillation” is 83 points.
