# Supplementary material for: Umbrella review and Delphi study on modifiable factors for dementia risk reduction
Source: Alzheimers Dement. 2023 Dec 30;20(3):2223–39. doi: 10.1002/alz.13577 (PMC10984497; doi:10.1002/alz.13577)
Supplement: Supplementary file 8 — Supporting Information [file ALZ-20-2223-s008.docx]

**Appendix H: Overview of associations in primary literature**

**Supplementary table 2:** Frequency and consistency of the factors (that are currently included in the LIBRA index) encountered in the primary literature based on the umbrella review of 148 systematic literature reviews and meta-analyses.

| **LIBRA factor** | **Number of studies** | **Higher risk/decline** | **No association** | **Lower risk/decline** | **Consistency of association** |
| --- | --- | --- | --- | --- | --- |
| Diabetes | 61 | 31 [1-31] | 30 [32-61] | 0 | 51% |
| Depression | 48 | 36 [47, 62-96] | 12 [97-108] | 0 | 75% |
| Midlife hypertension | 27 | 17 [109-125] | 8 [53, 126-132] | 2 [126, 133] | 63% |
| ^†^High leisure-time physical activity | 22 | 0 | 7 [49, 134-139] | 15 [12, 140-153] | 68% |
| ^*^High alcohol consumption | 22 | 3 [154-156] | 15 [141, 157-170] | 4 [103, 166, 171, 172] | 18% |
| Chronic kidney disease | 20 | 14 [173-186] | 6 [56, 187-191] | 0 | 70% |
| High cognitive activity | 20 | 0 | 7 [106, 192-197] | 13 [153, 198-209] | 68% |
| Healthy diet/Mediterranean diet | 18 | 0 | 8 [163, 210-216] | 10 [217-226] | 56% |
| Coronary heart disease | 15 | 10 [35, 44, 114, 227-233] | 5 [234-238] | 0 | 67% |
| Smoking | 14 | 6 [156, 239-243] | 8 [56, 103, 122, 244-248] | 0 | 43% |
| Midlife obesity | 9 | 2 [249, 250] | 7 [251-257] | 0 | 22% |
| High midlife cholesterol | 8 | 5 [114, 258-261] | 3 [122, 262, 263] | 0 | 63% |

NOTE. These factors were ordered based on their frequency and, subsequently, their consistency of association in the primary literature**.** For each factor, this was calculated as the highest number of studies that found a significant association in one direction (higher or lower risk) divided by the total amount of studies on that factor.

^*^Inverse of the operationalisation in the original LIBRA index (= low/moderate alcohol intake)
^†^Inverse of the operationalisation in the original LIBRA index (= physical inactivity)

Abbreviations: LIBRA, LIfestyle for BRAin health

**Supplementary table 3:** Frequency and consistency of the factors (that are currently not included in the LIBRA index) encountered in the primary literature based on the umbrella review of 148 systematic literature reviews and meta-analyses.

| **Non-LIBRA factor** | **Number of studies** | **Higher risk/decline** | **No association** | **Lower risk/decline** | **Consistency  of association** |
| --- | --- | --- | --- | --- | --- |
| Low social engagement | 28 | 15 [194, 204, 264-276] | 13 [198, 203, 206, 227, 277-285] | 0 | 54% |
| T2PD or impaired FBG | 19 | 4 [12, 15, 286, 287] | 15 [9, 20, 22-24, 45, 122, 288-295] | 0 | 21% |
| Hearing impairment | 18 | 14 [296-309] | 4 [47, 310-312] | 0 | 78% |
| Long sleep duration | 16 | 7 [163, 313-318] | 9 [319-327] | 0 | 44% |
| Short sleep duration | 16 | 5 [314, 315, 320, 324, 326] | 11 [163, 313, 316-319, 321-323, 325, 327] | 0 | 31% |
| Vision impairment | 15 | 10 [14, 300, 328-335] | 5 [311, 336-339] | 0 | 67% |
| Atrial fibrillation | 14 | 11 [27, 237, 340-348] | 3 [229, 349, 350] | 0 | 79% |
| Anxiety | 14 | 10 [62, 67, 75, 95, 351-356] | 4 [66, 357-359] | 0 | 71% |
| Low social network size | 11 | 2 [360, 361] | 9 [274, 281, 362-368] | 0 | 18% |
| Loneliness | 10 | 7 [27, 274, 369-373] | 3 [227, 281, 374] | 0 | 70% |
| Tooth loss | 10 | 7 [375-381] | 3 [382-384] | 0 | 70% |
| Poor sleep quality | 10 | 5 [68, 326, 385-387] | 5 [317, 319, 370, 388, 389] | 0 | 50% |
| Orthostatic hypotension | 10 | 4 [390-393] | 6 [128, 394-398] | 0 | 40% |
| Living alone | 10 | 3 [227, 369, 399] | 6 [281, 374, 400-403] | 1 [404] | 30% |
| Olfactory impairment | 9 | 9 [231, 300, 405-411] | 0 | 0 | 100% |
| Insomnia | 9 | 4 [316, 412-414] | 4 [415-418] | 1 [419] | 44% |
| Metabolic syndrome | 8 | 3 [420-422] | 5 [11, 39, 289, 294, 423] | 0 | 38% |
| Daytime sleepiness | 5 | 5 [322, 415, 416, 418, 419] | 0 | 0 | 100% |
| High religious involvement | 5 | 0 | 2 [424, 425] | 3 [273, 282, 426] | 60% |
| Heart failure | 5 | 2 [427, 428] | 3 [229, 237, 429] | 0 | 40% |
| Sleep-disordered breathing | 5 | 2 [430, 431] | 3 [415, 432, 433] | 0 | 40% |
| Low emotional support | 5 | 0 | 5 [265, 283, 362, 365, 366] | 0 | 0% |
| Any heart disease | 4 | 1 [228] | 3 [56, 69, 103] | 0 | 25% |
| Periodontal disease | 4 | 1 [434] | 3 [378, 381, 435] | 0 | 25% |
| High psychological stress | 3 | 3 [436-438] | 0 | 0 | 100% |
| Social difficulties | 3 | 3 [439-441] | 0 | 0 | 100% |
| Low serum folate | 3 | 1 [442] | 2 [443, 444] | 0 | 33% |
| Pesticide exposure | 3 | 1 [445] | 2 [56, 446] | 0 | 33% |
|  |  |  |  |  |  |

NOTE. These factors were ordered based on their frequency and, subsequently, their consistency of association in the primary literature. For each factor, this was calculated as the highest number of studies that found a significant association in one direction (higher or lower risk) divided by the total amount of studies on that factor. Factors encountered with a frequency ≤ 2 or that were too closely related to existing LIBRA factors, were omitted from this table
Abbreviations: FBG, Fasting blood glucose; LIBRA, LIfestyle for BRAin health; T2PD, Type-2 prediabetes

**References**

[1] Ahtiluoto S, Polvikoski T, Peltonen M, Solomon A, Tuomilehto J, Winblad B, et al. Diabetes, Alzheimer disease, and vascular dementia: a population-based neuropathologic study. Neurology. 2010;75:1195-202.

[2] Arvanitakis Z, Wilson RS, Bienias JL, Evans DA, Bennett DA. Diabetes mellitus and risk of Alzheimer disease and decline in cognitive function. Arch Neurol. 2004;61:661-6.

[3] Crane PK, Walker R, Larson EB. Glucose levels and risk of dementia. N Engl J Med. 2013;369:1863-4.

[4] Espeland MA, Miller ME, Goveas JS, Hogan PE, Coker LH, Williamson J, et al. Cognitive function and fine motor speed in older women with diabetes mellitus: results from the women's health initiative study of cognitive aging. J Womens Health (Larchmt). 2011;20:1435-43.

[5] Irie F, Fitzpatrick AL, Lopez OL, Kuller LH, Peila R, Newman AB, et al. Enhanced risk for Alzheimer disease in persons with type 2 diabetes and APOE epsilon4: the Cardiovascular Health Study Cognition Study. Arch Neurol. 2008;65:89-93.

[6] Kumari M, Marmot M. Diabetes and cognitive function in a middle-aged cohort: findings from the Whitehall II study. Neurology. 2005;65:1597-603.

[7] Marseglia A, Dahl Aslan AK, Fratiglioni L, Santoni G, Pedersen NL, Xu W. Cognitive Trajectories of Older Adults With Prediabetes and Diabetes: A Population-Based Cohort Study. J Gerontol A Biol Sci Med Sci. 2018;73:400-6.

[8] Marseglia A, Fratiglioni L, Kalpouzos G, Wang R, Bäckman L, Xu W. Prediabetes and diabetes accelerate cognitive decline and predict microvascular lesions: A population-based cohort study. Alzheimers Dement. 2019;15:25-33.

[9] Marseglia A, Wang HX, Rizzuto D, Fratiglioni L, Xu W. Participating in Mental, Social, and Physical Leisure Activities and Having a Rich Social Network Reduce the Incidence of Diabetes-Related Dementia in a Cohort of Swedish Older Adults. Diabetes Care. 2019;42:232-9.

[10] Mayeda ER, Haan MN, Kanaya AM, Yaffe K, Neuhaus J. Type 2 diabetes and 10-year risk of dementia and cognitive impairment among older Mexican Americans. Diabetes Care. 2013;36:2600-6.

[11] Muller M, Tang MX, Schupf N, Manly JJ, Mayeux R, Luchsinger JA. Metabolic syndrome and dementia risk in a multiethnic elderly cohort. Dement Geriatr Cogn Disord. 2007;24:185-92.

[12] Neergaard JS, Dragsbæk K, Hansen HB, Henriksen K, Christiansen C, Karsdal MA. Late-Life Risk Factors for All-Cause Dementia and Differential Dementia Diagnoses in Women: A Prospective Cohort Study. Medicine (Baltimore). 2016;95:e3112.

[13] Ng TP, Feng L, Nyunt MS, Feng L, Gao Q, Lim ML, et al. Metabolic Syndrome and the Risk of Mild Cognitive Impairment and Progression to Dementia: Follow-up of the Singapore Longitudinal Ageing Study Cohort. JAMA Neurol. 2016;73:456-63.

[14] Nguyen HT, Black SA, Ray LA, Espino DV, Markides KS. Predictors of decline in MMSE scores among older Mexican Americans. J Gerontol A Biol Sci Med Sci. 2002;57:M181-5.

[15] Ohara T, Doi Y, Ninomiya T, Hirakawa Y, Hata J, Iwaki T, et al. Glucose tolerance status and risk of dementia in the community: the Hisayama study. Neurology. 2011;77:1126-34.

[16] Okereke OI, Kang JH, Cook NR, Gaziano JM, Manson JE, Buring JE, et al. Type 2 diabetes mellitus and cognitive decline in two large cohorts of community-dwelling older adults. J Am Geriatr Soc. 2008;56:1028-36.

[17] Ott A, Stolk RP, van Harskamp F, Pols HA, Hofman A, Breteler MM. Diabetes mellitus and the risk of dementia: The Rotterdam Study. Neurology. 1999;53:1937-42.

[18] Pappas C, Andel R, Infurna FJ, Seetharaman S. Glycated haemoglobin (HbA1c), diabetes and trajectories of change in episodic memory performance. J Epidemiol Community Health. 2017;71:115-20.

[19] Peila R, Rodriguez BL, Launer LJ. Type 2 diabetes, APOE gene, and the risk for dementia and related pathologies: The Honolulu-Asia Aging Study. Diabetes. 2002;51:1256-62.

[20] Raffaitin C, Gin H, Empana JP, Helmer C, Berr C, Tzourio C, et al. Metabolic syndrome and risk for incident Alzheimer's disease or vascular dementia: the Three-City Study. Diabetes Care. 2009;32:169-74.

[21] Roberts RO, Knopman DS, Geda YE, Cha RH, Pankratz VS, Baertlein L, et al. Association of diabetes with amnestic and nonamnestic mild cognitive impairment. Alzheimers Dement. 2014;10:18-26.

[22] Rouch I, Roche F, Dauphinot V, Laurent B, Antérion CT, Celle S, et al. Diabetes, impaired fasting glucose, and cognitive decline in a population of elderly community residents. Aging Clin Exp Res. 2012;24:377-83.

[23] Salinas RM, Hiriart M, Acosta I, Sosa AL, Prince MJ. Type 2 diabetes mellitus as a risk factor for dementia in a Mexican population. J Diabetes Complications. 2016;30:1234-9.

[24] Samaras K, Lutgers HL, Kochan NA, Crawford JD, Campbell LV, Wen W, et al. The impact of glucose disorders on cognition and brain volumes in the elderly: the Sydney Memory and Ageing Study. Age (Dordr). 2014;36:977-93.

[25] Schnaider Beeri M, Goldbourt U, Silverman JM, Noy S, Schmeidler J, Ravona-Springer R, et al. Diabetes mellitus in midlife and the risk of dementia three decades later. Neurology. 2004;63:1902-7.

[26] Simeon V, Chiodini P, Mattiello A, Sieri S, Panico C, Brighenti F, et al. Dietary glycemic load and risk of cognitive impairment in women: findings from the EPIC-Naples cohort. Eur J Epidemiol. 2015;30:425-33.

[27] Tilvis RS, Kähönen-Väre MH, Jolkkonen J, Valvanne J, Pitkala KH, Strandberg TE. Predictors of cognitive decline and mortality of aged people over a 10-year period. J Gerontol A Biol Sci Med Sci. 2004;59:268-74.

[28] Tuligenga RH, Dugravot A, Tabák AG, Elbaz A, Brunner EJ, Kivimäki M, et al. Midlife type 2 diabetes and poor glycaemic control as risk factors for cognitive decline in early old age: a post-hoc analysis of the Whitehall II cohort study. Lancet Diabetes Endocrinol. 2014;2:228-35.

[29] Wessels AM, Lane KA, Gao S, Hall KS, Unverzagt FW, Hendrie HC. Diabetes and cognitive decline in elderly African Americans: a 15-year follow-up study. Alzheimers Dement. 2011;7:418-24.

[30] Yaffe K, Falvey C, Hamilton N, Schwartz AV, Simonsick EM, Satterfield S, et al. Diabetes, glucose control, and 9-year cognitive decline among older adults without dementia. Arch Neurol. 2012;69:1170-5.

[31] Yokomichi H, Kondo K, Nagamine Y, Yamagata Z, Kondo N. Dementia risk by combinations of metabolic diseases and body mass index: Japan Gerontological Evaluation Study Cohort Study. J Diabetes Investig. 2020;11:206-15.

[32] Akomolafe A, Beiser A, Meigs JB, Au R, Green RC, Farrer LA, et al. Diabetes mellitus and risk of developing Alzheimer disease: results from the Framingham Study. Arch Neurol. 2006;63:1551-5.

[33] Anstey KJ, Sargent-Cox K, Garde E, Cherbuin N, Butterworth P. Cognitive development over 8 years in midlife and its association with cardiovascular risk factors. Neuropsychology. 2014;28:653-65.

[34] Borenstein AR, Wu Y, Mortimer JA, Schellenberg GD, McCormick WC, Bowen JD, et al. Developmental and vascular risk factors for Alzheimer's disease. Neurobiol Aging. 2005;26:325-34.

[35] Brayne C, Gill C, Huppert FA, Barkley C, Gehlhaar E, Girling DM, et al. Vascular risks and incident dementia: results from a cohort study of the very old. Dement Geriatr Cogn Disord. 1998;9:175-80.

[36] Callisaya ML, Beare R, Moran C, Phan T, Wang W, Srikanth VK. Type 2 diabetes mellitus, brain atrophy and cognitive decline in older people: a longitudinal study. Diabetologia. 2019;62:448-58.

[37] Cheng C, Lin CH, Tsai YW, Tsai CJ, Chou PH, Lan TH. Type 2 diabetes and antidiabetic medications in relation to dementia diagnosis. J Gerontol A Biol Sci Med Sci. 2014;69:1299-305.

[38] Cholerton B, Omidpanah A, Verney SP, Nelson LA, Baker LD, Suchy-Dicey A, et al. Type 2 diabetes and later cognitive function in older American Indians: The Strong Heart Study. Int J Geriatr Psychiatry. 2019;34:1050-7.

[39] Creavin ST, Gallacher J, Bayer A, Fish M, Ebrahim S, Ben-Shlomo Y. Metabolic syndrome, diabetes, poor cognition, and dementia in the Caerphilly prospective study. J Alzheimers Dis. 2012;28:931-9.

[40] Debling D, Amelang M, Hasselbach P, Stürmer T. Diabetes and cognitive function in a population-based study of elderly women and men. J Diabetes Complications. 2006;20:238-45.

[41] Ganguli M, Fu B, Snitz BE, Hughes TF, Chang CC. Mild cognitive impairment: incidence and vascular risk factors in a population-based cohort. Neurology. 2013;80:2112-20.

[42] Gregg EW, Yaffe K, Cauley JA, Rolka DB, Blackwell TL, Narayan KM, et al. Is diabetes associated with cognitive impairment and cognitive decline among older women? Study of Osteoporotic Fractures Research Group. Arch Intern Med. 2000;160:174-80.

[43] Hassing LB, Johansson B, Nilsson SE, Berg S, Pedersen NL, Gatz M, et al. Diabetes mellitus is a risk factor for vascular dementia, but not for Alzheimer's disease: a population-based study of the oldest old. Int Psychogeriatr. 2002;14:239-48.

[44] Hayden KM, Zandi PP, Lyketsos CG, Khachaturian AS, Bastian LA, Charoonruk G, et al. Vascular risk factors for incident Alzheimer disease and vascular dementia: the Cache County study. Alzheimer Dis Assoc Disord. 2006;20:93-100.

[45] Kanaya AM, Barrett-Connor E, Gildengorin G, Yaffe K. Change in cognitive function by glucose tolerance status in older adults: a 4-year prospective study of the Rancho Bernardo study cohort. Arch Intern Med. 2004;164:1327-33.

[46] Logroscino G, Kang JH, Grodstein F. Prospective study of type 2 diabetes and cognitive decline in women aged 70-81 years. Bmj. 2004;328:548.

[47] Lyu J, Kim HY. Gender-Specific Incidence and Predictors of Cognitive Impairment among Older Koreans: Findings from a 6-Year Prospective Cohort Study. Psychiatry Investig. 2016;13:473-9.

[48] MacKnight C, Rockwood K, Awalt E, McDowell I. Diabetes mellitus and the risk of dementia, Alzheimer's disease and vascular cognitive impairment in the Canadian Study of Health and Aging. Dement Geriatr Cogn Disord. 2002;14:77-83.

[49] Mehlig K, Skoog I, Waern M, Miao Jonasson J, Lapidus L, Björkelund C, et al. Physical activity, weight status, diabetes and dementia: a 34-year follow-up of the population study of women in Gothenburg. Neuroepidemiology. 2014;42:252-9.

[50] Palta P, Carlson MC, Crum RM, Colantuoni E, Sharrett AR, Yasar S, et al. Diabetes and Cognitive Decline in Older Adults: The Ginkgo Evaluation of Memory Study. J Gerontol A Biol Sci Med Sci. 2017;73:123-30.

[51] Rawlings AM, Sharrett AR, Albert MS, Coresh J, Windham BG, Power MC, et al. The Association of Late-Life Diabetes Status and Hyperglycemia With Incident Mild Cognitive Impairment and Dementia: The ARIC Study. Diabetes Care. 2019;42:1248-54.

[52] Solfrizzi V, Panza F, Colacicco AM, D'Introno A, Capurso C, Torres F, et al. Vascular risk factors, incidence of MCI, and rates of progression to dementia. Neurology. 2004;63:1882-91.

[53] Stewart R, Prince M, Mann A. Age, vascular risk, and cognitive decline in an older, British, African-Caribbean population. J Am Geriatr Soc. 2003;51:1547-53.

[54] Tervo S, Kivipelto M, Hänninen T, Vanhanen M, Hallikainen M, Mannermaa A, et al. Incidence and risk factors for mild cognitive impairment: a population-based three-year follow-up study of cognitively healthy elderly subjects. Dement Geriatr Cogn Disord. 2004;17:196-203.

[55] Tufvesson E, Melander O, Minthon L, Persson M, Nilsson PM, Struck J, et al. Diabetes mellitus and elevated copeptin levels in middle age predict low cognitive speed after long-term follow-up. Dement Geriatr Cogn Disord. 2013;35:67-76.

[56] Tyas SL, Manfreda J, Strain LA, Montgomery PR. Risk factors for Alzheimer's disease: a population-based, longitudinal study in Manitoba, Canada. Int J Epidemiol. 2001;30:590-7.

[57] van den Berg E, de Craen AJ, Biessels GJ, Gussekloo J, Westendorp RG. The impact of diabetes mellitus on cognitive decline in the oldest of the old: a prospective population-based study. Diabetologia. 2006;49:2015-23.

[58] Verdelho A, Madureira S, Moleiro C, Ferro JM, Santos CO, Erkinjuntti T, et al. White matter changes and diabetes predict cognitive decline in the elderly: the LADIS study. Neurology. 2010;75:160-7.

[59] Viscogliosi G, Donfrancesco C, Palmieri L, Giampaoli S. The metabolic syndrome and 10-year cognitive and functional decline in very old men. A population-based study. Arch Gerontol Geriatr. 2017;70:62-6.

[60] Wennberg AMV, Hagen CE, Gottesman RF, Zipunnikov V, Kaufmann CN, Albert MS, et al. Longitudinal association between diabetes and cognitive decline: The National Health and Aging Trends Study. Arch Gerontol Geriatr. 2017;72:39-44.

[61] Xu WL, Qiu CX, Wahlin A, Winblad B, Fratiglioni L. Diabetes mellitus and risk of dementia in the Kungsholmen project: a 6-year follow-up study. Neurology. 2004;63:1181-6.

[62] Acosta I, Borges G, Aguirre-Hernandez R, Sosa AL, Prince M. Neuropsychiatric symptoms as risk factors of dementia in a Mexican population: A 10/66 Dementia Research Group study. Alzheimers Dement. 2018;14:271-9.

[63] Almeida OP, Hankey GJ, Yeap BB, Golledge J, Flicker L. Depression as a modifiable factor to decrease the risk of dementia. Transl Psychiatry. 2017;7:e1117.

[64] Bae JB, Kim YJ, Han JW, Kim TH, Park JH, Lee SB, et al. Incidence of and risk factors for Alzheimer's disease and mild cognitive impairment in Korean elderly. Dement Geriatr Cogn Disord. 2015;39:105-15.

[65] Barnes DE, Yaffe K, Byers AL, McCormick M, Schaefer C, Whitmer RA. Midlife vs late-life depressive symptoms and risk of dementia: differential effects for Alzheimer disease and vascular dementia. Arch Gen Psychiatry. 2012;69:493-8.

[66] Brodaty H, Heffernan M, Draper B, Reppermund S, Kochan NA, Slavin MJ, et al. Neuropsychiatric symptoms in older people with and without cognitive impairment. J Alzheimers Dis. 2012;31:411-20.

[67] Burke SL, Cadet T, Alcide A, O'Driscoll J, Maramaldi P. Psychosocial risk factors and Alzheimer's disease: the associative effect of depression, sleep disturbance, and anxiety. Aging Ment Health. 2018;22:1577-84.

[68] Burke SL, Maramaldi P, Cadet T, Kukull W. Associations between depression, sleep disturbance, and apolipoprotein E in the development of Alzheimer's disease: dementia. Int Psychogeriatr. 2016;28:1409-24.

[69] Dal Forno G, Palermo MT, Donohue JE, Karagiozis H, Zonderman AB, Kawas CH. Depressive symptoms, sex, and risk for Alzheimer's disease. Ann Neurol. 2005;57:381-7.

[70] Devanand DP, Sano M, Tang MX, Taylor S, Gurland BJ, Wilder D, et al. Depressed mood and the incidence of Alzheimer's disease in the elderly living in the community. Arch Gen Psychiatry. 1996;53:175-82.

[71] Donovan NJ, Amariglio RE, Zoller AS, Rudel RK, Gomez-Isla T, Blacker D, et al. Subjective cognitive concerns and neuropsychiatric predictors of progression to the early clinical stages of Alzheimer disease. Am J Geriatr Psychiatry. 2014;22:1642-51.

[72] Ezzati A, Katz MJ, Derby CA, Zimmerman ME, Lipton RB. Depressive Symptoms Predict Incident Dementia in a Community Sample of Older Adults: Results From the Einstein Aging Study. J Geriatr Psychiatry Neurol. 2019:891988718824036.

[73] Fuhrer R, Dufouil C, Dartigues JF. Exploring sex differences in the relationship between depressive symptoms and dementia incidence: prospective results from the PAQUID Study. J Am Geriatr Soc. 2003;51:1055-63.

[74] Gatz JL, Tyas SL, St John P, Montgomery P. Do depressive symptoms predict Alzheimer's disease and dementia? J Gerontol A Biol Sci Med Sci. 2005;60:744-7.

[75] Geda YE, Roberts RO, Mielke MM, Knopman DS, Christianson TJ, Pankratz VS, et al. Baseline neuropsychiatric symptoms and the risk of incident mild cognitive impairment: a population-based study. Am J Psychiatry. 2014;171:572-81.

[76] Geerlings MI, den Heijer T, Koudstaal PJ, Hofman A, Breteler MM. History of depression, depressive symptoms, and medial temporal lobe atrophy and the risk of Alzheimer disease. Neurology. 2008;70:1258-64.

[77] Goveas JS, Espeland MA, Woods NF, Wassertheil-Smoller S, Kotchen JM. Depressive symptoms and incidence of mild cognitive impairment and probable dementia in elderly women: the Women's Health Initiative Memory Study. J Am Geriatr Soc. 2011;59:57-66.

[78] Gracia-García P, de-la-Cámara C, Santabárbara J, Lopez-Anton R, Quintanilla MA, Ventura T, et al. Depression and incident Alzheimer disease: the impact of disease severity. Am J Geriatr Psychiatry. 2015;23:119-29.

[79] Heun R, Kölsch H, Jessen F. Risk factors and early signs of Alzheimer's disease in a family study sample. Risk of AD. Eur Arch Psychiatry Clin Neurosci. 2006;256:28-36.

[80] Irie F, Masaki KH, Petrovitch H, Abbott RD, Ross GW, Taaffe DR, et al. Apolipoprotein E epsilon4 allele genotype and the effect of depressive symptoms on the risk of dementia in men: the Honolulu-Asia Aging Study. Arch Gen Psychiatry. 2008;65:906-12.

[81] Kaup AR, Byers AL, Falvey C, Simonsick EM, Satterfield S, Ayonayon HN, et al. Trajectories of Depressive Symptoms in Older Adults and Risk of Dementia. JAMA Psychiatry. 2016;73:525-31.

[82] Köhler S, van Boxtel M, Jolles J, Verhey F. Depressive symptoms and risk for dementia: a 9-year follow-up of the Maastricht Aging Study. Am J Geriatr Psychiatry. 2011;19:902-5.

[83] Kontari P, Smith KJ. Risk of dementia associated with cardiometabolic abnormalities and depressive symptoms: a longitudinal cohort study using the English longitudinal study of ageing. Int J Geriatr Psychiatry. 2019;34:289-98.

[84] Lenoir H, Dufouil C, Auriacombe S, Lacombe JM, Dartigues JF, Ritchie K, et al. Depression history, depressive symptoms, and incident dementia: the 3C Study. J Alzheimers Dis. 2011;26:27-38.

[85] Li G, Wang LY, Shofer JB, Thompson ML, Peskind ER, McCormick W, et al. Temporal relationship between depression and dementia: findings from a large community-based 15-year follow-up study. Arch Gen Psychiatry. 2011;68:970-7.

[86] Lugtenburg A, Zuidersma M, Oude Voshaar RC, Schoevers RA. Symptom Dimensions of Depression and 3-Year Incidence of Dementia: Results From the Amsterdam Study of the Elderly. J Geriatr Psychiatry Neurol. 2016;29:99-107.

[87] Luppa M, Luck T, Ritschel F, Angermeyer MC, Villringer A, Riedel-Heller SG. Depression and incident dementia. An 8-year population-based prospective study. PLoS One. 2013;8:e59246.

[88] Makizako H, Shimada H, Doi T, Tsutsumimoto K, Hotta R, Nakakubo S, et al. Comorbid Mild Cognitive Impairment and Depressive Symptoms Predict Future Dementia in Community Older Adults: A 24-Month Follow-Up Longitudinal Study. J Alzheimers Dis. 2016;54:1473-82.

[89] Mirza SS, Wolters FJ, Swanson SA, Koudstaal PJ, Hofman A, Tiemeier H, et al. 10-year trajectories of depressive symptoms and risk of dementia: a population-based study. Lancet Psychiatry. 2016;3:628-35.

[90] Saczynski JS, Beiser A, Seshadri S, Auerbach S, Wolf PA, Au R. Depressive symptoms and risk of dementia: the Framingham Heart Study. Neurology. 2010;75:35-41.

[91] Spira AP, Rebok GW, Stone KL, Kramer JH, Yaffe K. Depressive symptoms in oldest-old women: risk of mild cognitive impairment and dementia. Am J Geriatr Psychiatry. 2012;20:1006-15.

[92] Verdelho A, Madureira S, Moleiro C, Ferro JM, O'Brien JT, Poggesi A, et al. Depressive symptoms predict cognitive decline and dementia in older people independently of cerebral white matter changes: the LADIS study. J Neurol Neurosurg Psychiatry. 2013;84:1250-4.

[93] Vilalta-Franch J, López-Pousa S, Llinàs-Reglà J, Calvó-Perxas L, Merino-Aguado J, Garre-Olmo J. Depression subtypes and 5-year risk of dementia and Alzheimer disease in patients aged 70 years. Int J Geriatr Psychiatry. 2013;28:341-50.

[94] Wallin K, Boström G, Kivipelto M, Gustafson Y. Risk factors for incident dementia in the very old. Int Psychogeriatr. 2013;25:1135-43.

[95] Wilson RS, Begeny CT, Boyle PA, Schneider JA, Bennett DA. Vulnerability to stress, anxiety, and development of dementia in old age. Am J Geriatr Psychiatry. 2011;19:327-34.

[96] Zahodne LB, Schupf N, Brickman AM, Mayeux R, Wall MM, Stern Y, et al. Dementia Risk and Protective Factors Differ in the Context of Memory Trajectory Groups. J Alzheimers Dis. 2016;52:1013-20.

[97] Andersen K, Lolk A, Kragh-Sørensen P, Petersen NE, Green A. Depression and the risk of Alzheimer disease. Epidemiology. 2005;16:233-8.

[98] Annweiler C, Rolland Y, Schott AM, Blain H, Vellas B, Herrmann FR, et al. Higher vitamin D dietary intake is associated with lower risk of alzheimer's disease: a 7-year follow-up. J Gerontol A Biol Sci Med Sci. 2012;67:1205-11.

[99] Becker JT, Chang YF, Lopez OL, Dew MA, Sweet RA, Barnes D, et al. Depressed mood is not a risk factor for incident dementia in a community-based cohort. Am J Geriatr Psychiatry. 2009;17:653-63.

[100] Chen P, Ganguli M, Mulsant BH, DeKosky ST. The temporal relationship between depressive symptoms and dementia: a community-based prospective study. Arch Gen Psychiatry. 1999;56:261-6.

[101] Gale CR, Allerhand M, Deary IJ. Is there a bidirectional relationship between depressive symptoms and cognitive ability in older people? A prospective study using the English Longitudinal Study of Ageing. Psychol Med. 2012;42:2057-69.

[102] Kim JM, Kim SY, Bae KY, Kim SW, Shin IS, Yang SJ, et al. Apolipoprotein e4 genotype and depressive symptoms as risk factors for dementia in an older korean population. Psychiatry Investig. 2010;7:135-40.

[103] Lindsay J, Laurin D, Verreault R, Hébert R, Helliwell B, Hill GB, et al. Risk factors for Alzheimer's disease: a prospective analysis from the Canadian Study of Health and Aging. Am J Epidemiol. 2002;156:445-53.

[104] Mossaheb N, Zehetmayer S, Jungwirth S, Weissgram S, Rainer M, Tragl KH, et al. Are specific symptoms of depression predictive of Alzheimer's dementia? J Clin Psychiatry. 2012;73:1009-15.

[105] Pálsson S, Aevarsson O, Skoog I. Depression, cerebral atrophy, cognitive performance and incidence of dementia. Population study of 85-year-olds. Br J Psychiatry. 1999;174:249-53.

[106] Sattler C, Toro P, Schönknecht P, Schröder J. Cognitive activity, education and socioeconomic status as preventive factors for mild cognitive impairment and Alzheimer's disease. Psychiatry Res. 2012;196:90-5.

[107] Singh-Manoux A, Dugravot A, Fournier A, Abell J, Ebmeier K, Kivimäki M, et al. Trajectories of Depressive Symptoms Before Diagnosis of Dementia: A 28-Year Follow-up Study. JAMA Psychiatry. 2017;74:712-8.

[108] Vinkers DJ, Gussekloo J, Stek ML, Westendorp RG, van der Mast RC. Temporal relation between depression and cognitive impairment in old age: prospective population based study. Bmj. 2004;329:881.

[109] Abell JG, Kivimäki M, Dugravot A, Tabak AG, Fayosse A, Shipley M, et al. Association between systolic blood pressure and dementia in the Whitehall II cohort study: role of age, duration, and threshold used to define hypertension. Eur Heart J. 2018;39:3119-25.

[110] Debette S, Seshadri S, Beiser A, Au R, Himali JJ, Palumbo C, et al. Midlife vascular risk factor exposure accelerates structural brain aging and cognitive decline. Neurology. 2011;77:461-8.

[111] Dregan A, Stewart R, Gulliford MC. Cardiovascular risk factors and cognitive decline in adults aged 50 and over: a population-based cohort study. Age Ageing. 2013;42:338-45.

[112] Freitag MH, Peila R, Masaki K, Petrovitch H, Ross GW, White LR, et al. Midlife pulse pressure and incidence of dementia: the Honolulu-Asia Aging Study. Stroke. 2006;37:33-7.

[113] Gottesman RF, Albert MS, Alonso A, Coker LH, Coresh J, Davis SM, et al. Associations Between Midlife Vascular Risk Factors and 25-Year Incident Dementia in the Atherosclerosis Risk in Communities (ARIC) Cohort. JAMA Neurol. 2017;74:1246-54.

[114] Kivipelto M, Helkala EL, Laakso MP, Hänninen T, Hallikainen M, Alhainen K, et al. Apolipoprotein E epsilon4 allele, elevated midlife total cholesterol level, and high midlife systolic blood pressure are independent risk factors for late-life Alzheimer disease. Ann Intern Med. 2002;137:149-55.

[115] Kivipelto M, Ngandu T, Laatikainen T, Winblad B, Soininen H, Tuomilehto J. Risk score for the prediction of dementia risk in 20 years among middle aged people: a longitudinal, population-based study. Lancet Neurol. 2006;5:735-41.

[116] Köhler S, Baars MA, Spauwen P, Schievink S, Verhey FR, van Boxtel MJ. Temporal evolution of cognitive changes in incident hypertension: prospective cohort study across the adult age span. Hypertension. 2014;63:245-51.

[117] Liu J, Huang Y, Chen G, Liu X, Wang Z, Cao Y, et al. Cumulative systolic blood pressure exposure in relation to cognitive function in middle-aged and elderly adults: A prospective, population-based study. Medicine (Baltimore). 2016;95:e5514.

[118] McGrath ER, Beiser AS, DeCarli C, Plourde KL, Vasan RS, Greenberg SM, et al. Blood pressure from mid- to late life and risk of incident dementia. Neurology. 2017;89:2447-54.

[119] Meyer JS, Rauch G, Rauch RA, Haque A. Risk factors for cerebral hypoperfusion, mild cognitive impairment, and dementia. Neurobiol Aging. 2000;21:161-9.

[120] Ninomiya T, Ohara T, Hirakawa Y, Yoshida D, Doi Y, Hata J, et al. Midlife and late-life blood pressure and dementia in Japanese elderly: the Hisayama study. Hypertension. 2011;58:22-8.

[121] Roberts RO, Cha RH, Mielke MM, Geda YE, Boeve BF, Machulda MM, et al. Risk and protective factors for cognitive impairment in persons aged 85 years and older. Neurology. 2015;84:1854-61.

[122] Rönnemaa E, Zethelius B, Lannfelt L, Kilander L. Vascular risk factors and dementia: 40-year follow-up of a population-based cohort. Dement Geriatr Cogn Disord. 2011;31:460-6.

[123] Tarraf W, Rodríguez CJ, Daviglus ML, Lamar M, Schneiderman N, Gallo L, et al. Blood Pressure and Hispanic/Latino Cognitive Function: Hispanic Community Health Study/Study of Latinos Results. J Alzheimers Dis. 2017;59:31-42.

[124] Unverzagt FW, McClure LA, Wadley VG, Jenny NS, Go RC, Cushman M, et al. Vascular risk factors and cognitive impairment in a stroke-free cohort. Neurology. 2011;77:1729-36.

[125] Yamada M, Kasagi F, Sasaki H, Masunari N, Mimori Y, Suzuki G. Association between dementia and midlife risk factors: the Radiation Effects Research Foundation Adult Health Study. J Am Geriatr Soc. 2003;51:410-4.

[126] Gabin JM, Tambs K, Saltvedt I, Sund E, Holmen J. Association between blood pressure and Alzheimer disease measured up to 27 years prior to diagnosis: the HUNT Study. Alzheimers Res Ther. 2017;9:37.

[127] Hietanen H, Pietilä A, Kähönen M, Salomaa V. Ankle blood pressure and dementia: a prospective follow-up study. Blood Press Monit. 2013;18:16-20.

[128] Holm H, Nägga K, Nilsson ED, Melander O, Minthon L, Bachus E, et al. Longitudinal and postural changes of blood pressure predict dementia: the Malmö Preventive Project. Eur J Epidemiol. 2017;32:327-36.

[129] Matsumoto A, Satoh M, Kikuya M, Ohkubo T, Hirano M, Inoue R, et al. Day-to-day variability in home blood pressure is associated with cognitive decline: the Ohasama study. Hypertension. 2014;63:1333-8.

[130] Rosengren A, Skoog I, Gustafson D, Wilhelmsen L. Body mass index, other cardiovascular risk factors, and hospitalization for dementia. Arch Intern Med. 2005;165:321-6.

[131] Taylor C, Tillin T, Chaturvedi N, Dewey M, Ferri CP, Hughes A, et al. Midlife hypertensive status and cognitive function 20 years later: the Southall and Brent revisited study. J Am Geriatr Soc. 2013;61:1489-98.

[132] Virta JJ, Heikkilä K, Perola M, Koskenvuo M, Räihä I, Rinne JO, et al. Midlife cardiovascular risk factors and late cognitive impairment. Eur J Epidemiol. 2013;28:405-16.

[133] Rasmussen KL, Tybjærg-Hansen A, Nordestgaard BG, Frikke-Schmidt R. Absolute 10-year risk of dementia by age, sex and APOE genotype: a population-based cohort study. Cmaj. 2018;190:E1033-e41.

[134] Chang M, Jonsson PV, Snaedal J, Bjornsson S, Saczynski JS, Aspelund T, et al. The effect of midlife physical activity on cognitive function among older adults: AGES--Reykjavik Study. J Gerontol A Biol Sci Med Sci. 2010;65:1369-74.

[135] de Bruijn RF, Schrijvers EM, de Groot KA, Witteman JC, Hofman A, Franco OH, et al. The association between physical activity and dementia in an elderly population: the Rotterdam Study. Eur J Epidemiol. 2013;28:277-83.

[136] Morgan GS, Gallacher J, Bayer A, Fish M, Ebrahim S, Ben-Shlomo Y. Physical activity in middle-age and dementia in later life: findings from a prospective cohort of men in Caerphilly, South Wales and a meta-analysis. J Alzheimers Dis. 2012;31:569-80.

[137] Ravaglia G, Forti P, Lucicesare A, Pisacane N, Rietti E, Bianchin M, et al. Physical activity and dementia risk in the elderly: findings from a prospective Italian study. Neurology. 2008;70:1786-94.

[138] Sabia S, Dugravot A, Dartigues JF, Abell J, Elbaz A, Kivimäki M, et al. Physical activity, cognitive decline, and risk of dementia: 28 year follow-up of Whitehall II cohort study. Bmj. 2017;357:j2709.

[139] Taaffe DR, Irie F, Masaki KH, Abbott RD, Petrovitch H, Ross GW, et al. Physical activity, physical function, and incident dementia in elderly men: the Honolulu-Asia Aging Study. J Gerontol A Biol Sci Med Sci. 2008;63:529-35.

[140] Abbott RD, White LR, Ross GW, Masaki KH, Curb JD, Petrovitch H. Walking and dementia in physically capable elderly men. Jama. 2004;292:1447-53.

[141] Bowen ME. A prospective examination of the relationship between physical activity and dementia risk in later life. Am J Health Promot. 2012;26:333-40.

[142] Elwood P, Galante J, Pickering J, Palmer S, Bayer A, Ben-Shlomo Y, et al. Healthy lifestyles reduce the incidence of chronic diseases and dementia: evidence from the Caerphilly cohort study. PLoS One. 2013;8:e81877.

[143] Ho SC, Woo J, Sham A, Chan SG, Yu AL. A 3-year follow-up study of social, lifestyle and health predictors of cognitive impairment in a Chinese older cohort. Int J Epidemiol. 2001;30:1389-96.

[144] Karp A, Paillard-Borg S, Wang HX, Silverstein M, Winblad B, Fratiglioni L. Mental, physical and social components in leisure activities equally contribute to decrease dementia risk. Dement Geriatr Cogn Disord. 2006;21:65-73.

[145] Kishimoto H, Ohara T, Hata J, Ninomiya T, Yoshida D, Mukai N, et al. The long-term association between physical activity and risk of dementia in the community: the Hisayama Study. Eur J Epidemiol. 2016;31:267-74.

[146] Larson EB, Wang L, Bowen JD, McCormick WC, Teri L, Crane P, et al. Exercise is associated with reduced risk for incident dementia among persons 65 years of age and older. Ann Intern Med. 2006;144:73-81.

[147] Laurin D, Verreault R, Lindsay J, MacPherson K, Rockwood K. Physical activity and risk of cognitive impairment and dementia in elderly persons. Arch Neurol. 2001;58:498-504.

[148] Lee AT, Richards M, Chan WC, Chiu HF, Lee RS, Lam LC. Intensity and Types of Physical Exercise in Relation to Dementia Risk Reduction in Community-Living Older Adults. J Am Med Dir Assoc. 2015;16:899.e1-7.

[149] Luck T, Riedel-Heller SG, Luppa M, Wiese B, Köhler M, Jessen F, et al. Apolipoprotein E epsilon 4 genotype and a physically active lifestyle in late life: analysis of gene-environment interaction for the risk of dementia and Alzheimer's disease dementia. Psychol Med. 2014;44:1319-29.

[150] Podewils LJ, Guallar E, Kuller LH, Fried LP, Lopez OL, Carlson M, et al. Physical activity, APOE genotype, and dementia risk: findings from the Cardiovascular Health Cognition Study. Am J Epidemiol. 2005;161:639-51.

[151] Rovio S, Kåreholt I, Helkala EL, Viitanen M, Winblad B, Tuomilehto J, et al. Leisure-time physical activity at midlife and the risk of dementia and Alzheimer's disease. Lancet Neurol. 2005;4:705-11.

[152] Scarmeas N, Luchsinger JA, Schupf N, Brickman AM, Cosentino S, Tang MX, et al. Physical activity, diet, and risk of Alzheimer disease. Jama. 2009;302:627-37.

[153] Wang HX, Jin Y, Hendrie HC, Liang C, Yang L, Cheng Y, et al. Late life leisure activities and risk of cognitive decline. J Gerontol A Biol Sci Med Sci. 2013;68:205-13.

[154] Handing EP, Andel R, Kadlecova P, Gatz M, Pedersen NL. Midlife Alcohol Consumption and Risk of Dementia Over 43 Years of Follow-Up: A Population-Based Study From the Swedish Twin Registry. J Gerontol A Biol Sci Med Sci. 2015;70:1248-54.

[155] Langballe EM, Ask H, Holmen J, Stordal E, Saltvedt I, Selbæk G, et al. Alcohol consumption and risk of dementia up to 27 years later in a large, population-based sample: the HUNT study, Norway. Eur J Epidemiol. 2015;30:1049-56.

[156] Zhou S, Zhou R, Zhong T, Li R, Tan J, Zhou H. Association of smoking and alcohol drinking with dementia risk among elderly men in China. Curr Alzheimer Res. 2014;11:899-907.

[157] Anttila T, Helkala EL, Viitanen M, Kåreholt I, Fratiglioni L, Winblad B, et al. Alcohol drinking in middle age and subsequent risk of mild cognitive impairment and dementia in old age: a prospective population based study. Bmj. 2004;329:539.

[158] Broe GA, Creasey H, Jorm AF, Bennett HP, Casey B, Waite LM, et al. Health habits and risk of cognitive impairment and dementia in old age: a prospective study on the effects of exercise, smoking and alcohol consumption. Aust N Z J Public Health. 1998;22:621-3.

[159] Deng J, Zhou DH, Li J, Wang YJ, Gao C, Chen M. A 2-year follow-up study of alcohol consumption and risk of dementia. Clin Neurol Neurosurg. 2006;108:378-83.

[160] Heffernan M, Mather KA, Xu J, Assareh AA, Kochan NA, Reppermund S, et al. Alcohol Consumption and Incident Dementia: Evidence from the Sydney Memory and Ageing Study. J Alzheimers Dis. 2016;52:529-38.

[161] Järvenpää T, Rinne JO, Koskenvuo M, Räihä I, Kaprio J. Binge drinking in midlife and dementia risk. Epidemiology. 2005;16:766-71.

[162] Koch M, Fitzpatrick AL, Rapp SR, Nahin RL, Williamson JD, Lopez OL, et al. Alcohol Consumption and Risk of Dementia and Cognitive Decline Among Older Adults With or Without Mild Cognitive Impairment. JAMA Netw Open. 2019;2:e1910319.

[163] Larsson SC, Wolk A. The Role of Lifestyle Factors and Sleep Duration for Late-Onset Dementia: A Cohort Study. J Alzheimers Dis. 2018;66:579-86.

[164] Luchsinger JA, Tang MX, Siddiqui M, Shea S, Mayeux R. Alcohol intake and risk of dementia. J Am Geriatr Soc. 2004;52:540-6.

[165] Mukamal KJ, Kuller LH, Fitzpatrick AL, Longstreth WT, Jr., Mittleman MA, Siscovick DS. Prospective study of alcohol consumption and risk of dementia in older adults. Jama. 2003;289:1405-13.

[166] Ogunniyi A, Hall KS, Gureje O, Baiyewu O, Gao S, Unverzagt FW, et al. Risk factors for incident Alzheimer's disease in African Americans and Yoruba. Metab Brain Dis. 2006;21:235-40.

[167] Paganini-Hill A, Kawas CH, Corrada MM. Lifestyle Factors and Dementia in the Oldest-old: The 90+ Study. Alzheimer Dis Assoc Disord. 2016;30:21-6.

[168] Ruitenberg A, van Swieten JC, Witteman JC, Mehta KM, van Duijn CM, Hofman A, et al. Alcohol consumption and risk of dementia: the Rotterdam Study. Lancet. 2002;359:281-6.

[169] Sabia S, Fayosse A, Dumurgier J, Dugravot A, Akbaraly T, Britton A, et al. Alcohol consumption and risk of dementia: 23 year follow-up of Whitehall II cohort study. Bmj. 2018;362:k2927.

[170] Yoshitake T, Kiyohara Y, Kato I, Ohmura T, Iwamoto H, Nakayama K, et al. Incidence and risk factors of vascular dementia and Alzheimer's disease in a defined elderly Japanese population: the Hisayama Study. Neurology. 1995;45:1161-8.

[171] Espeland MA, Gu L, Masaki KH, Langer RD, Coker LH, Stefanick ML, et al. Association between reported alcohol intake and cognition: results from the Women's Health Initiative Memory Study. Am J Epidemiol. 2005;161:228-38.

[172] Huang W, Qiu C, Winblad B, Fratiglioni L. Alcohol consumption and incidence of dementia in a community sample aged 75 years and older. J Clin Epidemiol. 2002;55:959-64.

[173] Buchman AS, Tanne D, Boyle PA, Shah RC, Leurgans SE, Bennett DA. Kidney function is associated with the rate of cognitive decline in the elderly. Neurology. 2009;73:920-7.

[174] Darsie B, Shlipak MG, Sarnak MJ, Katz R, Fitzpatrick AL, Odden MC. Kidney function and cognitive health in older adults: the Cardiovascular Health Study. Am J Epidemiol. 2014;180:68-75.

[175] Davey A, Elias MF, Robbins MA, Seliger SL, Dore GA. Decline in renal functioning is associated with longitudinal decline in global cognitive functioning, abstract reasoning and verbal memory. Nephrol Dial Transplant. 2013;28:1810-9.

[176] Etgen T, Sander D, Chonchol M, Briesenick C, Poppert H, Förstl H, et al. Chronic kidney disease is associated with incident cognitive impairment in the elderly: the INVADE study. Nephrol Dial Transplant. 2009;24:3144-50.

[177] Feng L, Yap KB, Yeoh LY, Ng TP. Kidney function and cognitive and functional decline in elderly adults: findings from the Singapore longitudinal aging study. J Am Geriatr Soc. 2012;60:1208-14.

[178] Higuchi M, Chen R, Abbott RD, Bell C, Launer L, Ross GW, et al. Mid-life proteinuria and late-life cognitive function and dementia in elderly men: the Honolulu-Asia Aging Study. Alzheimer Dis Assoc Disord. 2015;29:200-5.

[179] Jassal SK, Kritz-Silverstein D, Barrett-Connor E. A prospective study of albuminuria and cognitive function in older adults: the Rancho Bernardo study. Am J Epidemiol. 2010;171:277-86.

[180] Khatri M, Nickolas T, Moon YP, Paik MC, Rundek T, Elkind MS, et al. CKD associates with cognitive decline. J Am Soc Nephrol. 2009;20:2427-32.

[181] Kurella M, Chertow GM, Fried LF, Cummings SR, Harris T, Simonsick E, et al. Chronic kidney disease and cognitive impairment in the elderly: the health, aging, and body composition study. J Am Soc Nephrol. 2005;16:2127-33.

[182] Sajjad I, Grodstein F, Kang JH, Curhan GC, Lin J. Kidney dysfunction and cognitive decline in women. Clin J Am Soc Nephrol. 2012;7:437-43.

[183] Seliger SL, Siscovick DS, Stehman-Breen CO, Gillen DL, Fitzpatrick A, Bleyer A, et al. Moderate renal impairment and risk of dementia among older adults: the Cardiovascular Health Cognition Study. J Am Soc Nephrol. 2004;15:1904-11.

[184] Seliger SL, Wendell CR, Waldstein SR, Ferrucci L, Zonderman AB. Renal function and long-term decline in cognitive function: the Baltimore Longitudinal Study of Aging. Am J Nephrol. 2015;41:305-12.

[185] Sundelöf J, Arnlöv J, Ingelsson E, Sundström J, Basu S, Zethelius B, et al. Serum cystatin C and the risk of Alzheimer disease in elderly men. Neurology. 2008;71:1072-9.

[186] Wang F, Zhang L, Liu L, Wang H. Level of kidney function correlates with cognitive decline. Am J Nephrol. 2010;32:117-21.

[187] Helmer C, Stengel B, Metzger M, Froissart M, Massy ZA, Tzourio C, et al. Chronic kidney disease, cognitive decline, and incident dementia: the 3C Study. Neurology. 2011;77:2043-51.

[188] Kurella Tamura M, Muntner P, Wadley V, Cushman M, Zakai NA, Bradbury BD, et al. Albuminuria, kidney function, and the incidence of cognitive impairment among adults in the United States. Am J Kidney Dis. 2011;58:756-63.

[189] O'Hare AM, Walker R, Haneuse S, Crane PK, McCormick WC, Bowen JD, et al. Relationship between longitudinal measures of renal function and onset of dementia in a community cohort of older adults. J Am Geriatr Soc. 2012;60:2215-22.

[190] Slinin Y, Paudel ML, Ishani A, Taylor BC, Yaffe K, Murray AM, et al. Kidney function and cognitive performance and decline in older men. J Am Geriatr Soc. 2008;56:2082-8.

[191] Slinin Y, Peters KW, Ishani A, Yaffe K, Fink HA, Stone KL, et al. Cystatin C and cognitive impairment 10 years later in older women. J Gerontol A Biol Sci Med Sci. 2015;70:771-8.

[192] Lawton DM, Gasquoine PG, Weimer AA. Age of dementia diagnosis in community dwelling bilingual and monolingual Hispanic Americans. Cortex. 2015;66:141-5.

[193] Ljungberg JK, Hansson P, Adolfsson R, Nilsson L-G. The effect of language skills on dementia in a Swedish longitudinal cohort. Linguistic Approaches to Bilingualism. 2016;6:190-204.

[194] Paillard-Borg S, Fratiglioni L, Winblad B, Wang HX. Leisure activities in late life in relation to dementia risk: principal component analysis. Dement Geriatr Cogn Disord. 2009;28:136-44.

[195] Sanders AE, Hall CB, Katz MJ, Lipton RB. Non-native language use and risk of incident dementia in the elderly. J Alzheimers Dis. 2012;29:99-108.

[196] Sörman DE, Sundström A, Rönnlund M, Adolfsson R, Nilsson LG. Leisure activity in old age and risk of dementia: a 15-year prospective study. J Gerontol B Psychol Sci Soc Sci. 2014;69:493-501.

[197] Yeung CM, St John PD, Menec V, Tyas SL. Is bilingualism associated with a lower risk of dementia in community-living older adults? Cross-sectional and prospective analyses. Alzheimer Dis Assoc Disord. 2014;28:326-32.

[198] Akbaraly TN, Portet F, Fustinoni S, Dartigues JF, Artero S, Rouaud O, et al. Leisure activities and the risk of dementia in the elderly: results from the Three-City Study. Neurology. 2009;73:854-61.

[199] Almeida OP, Yeap BB, Alfonso H, Hankey GJ, Flicker L, Norman PE. Older men who use computers have lower risk of dementia. PLoS One. 2012;7:e44239.

[200] Carlson MC, Parisi JM, Xia J, Xue QL, Rebok GW, Bandeen-Roche K, et al. Lifestyle activities and memory: variety may be the spice of life. The women's health and aging study II. J Int Neuropsychol Soc. 2012;18:286-94.

[201] Hack EE, Dubin JA, Fernandes MA, Costa SM, Tyas SL. Multilingualism and Dementia Risk: Longitudinal Analysis of the Nun Study. J Alzheimers Dis. 2019;71:201-12.

[202] Hughes TF, Chang CC, Vander Bilt J, Ganguli M. Engagement in reading and hobbies and risk of incident dementia: the MoVIES project. Am J Alzheimers Dis Other Demen. 2010;25:432-8.

[203] Iwasa H, Yoshida Y, Kai I, Suzuki T, Kim H, Yoshida H. Leisure activities and cognitive function in elderly community-dwelling individuals in Japan: a 5-year prospective cohort study. J Psychosom Res. 2012;72:159-64.

[204] Scarmeas N, Levy G, Tang MX, Manly J, Stern Y. Influence of leisure activity on the incidence of Alzheimer's disease. Neurology. 2001;57:2236-42.

[205] Verghese J, Lipton RB, Katz MJ, Hall CB, Derby CA, Kuslansky G, et al. Leisure activities and the risk of dementia in the elderly. N Engl J Med. 2003;348:2508-16.

[206] Wang JY, Zhou DH, Li J, Zhang M, Deng J, Tang M, et al. Leisure activity and risk of cognitive impairment: the Chongqing aging study. Neurology. 2006;66:911-3.

[207] Wilson RS, Bennett DA, Bienias JL, Aggarwal NT, Mendes De Leon CF, Morris MC, et al. Cognitive activity and incident AD in a population-based sample of older persons. Neurology. 2002;59:1910-4.

[208] Wilson RS, Mendes De Leon CF, Barnes LL, Schneider JA, Bienias JL, Evans DA, et al. Participation in cognitively stimulating activities and risk of incident Alzheimer disease. Jama. 2002;287:742-8.

[209] Wilson RS, Scherr PA, Schneider JA, Tang Y, Bennett DA. Relation of cognitive activity to risk of developing Alzheimer disease. Neurology. 2007;69:1911-20.

[210] Féart C, Samieri C, Rondeau V, Amieva H, Portet F, Dartigues JF, et al. Adherence to a Mediterranean diet, cognitive decline, and risk of dementia. Jama. 2009;302:638-48.

[211] Haring B, Wu C, Mossavar-Rahmani Y, Snetselaar L, Brunner R, Wallace RB, et al. No Association between Dietary Patterns and Risk for Cognitive Decline in Older Women with 9-Year Follow-Up: Data from the Women's Health Initiative Memory Study. J Acad Nutr Diet. 2016;116:921-30.e1.

[212] Hosking DE, Eramudugolla R, Cherbuin N, Anstey KJ. MIND not Mediterranean diet related to 12-year incidence of cognitive impairment in an Australian longitudinal cohort study. Alzheimers Dement. 2019;15:581-9.

[213] Koyama A, Houston DK, Simonsick EM, Lee JS, Ayonayon HN, Shahar DR, et al. Association between the Mediterranean diet and cognitive decline in a biracial population. J Gerontol A Biol Sci Med Sci. 2015;70:354-9.

[214] Roberts RO, Geda YE, Cerhan JR, Knopman DS, Cha RH, Christianson TJ, et al. Vegetables, unsaturated fats, moderate alcohol intake, and mild cognitive impairment. Dement Geriatr Cogn Disord. 2010;29:413-23.

[215] Samieri C, Grodstein F, Rosner BA, Kang JH, Cook NR, Manson JE, et al. Mediterranean diet and cognitive function in older age. Epidemiology. 2013;24:490-9.

[216] Samieri C, Okereke OI, E ED, Grodstein F. Long-term adherence to the Mediterranean diet is associated with overall cognitive status, but not cognitive decline, in women. J Nutr. 2013;143:493-9.

[217] Galbete C, Toledo E, Toledo JB, Bes-Rastrollo M, Buil-Cosiales P, Marti A, et al. Mediterranean diet and cognitive function: the SUN project. J Nutr Health Aging. 2015;19:305-12.

[218] Morris MC, Tangney CC, Wang Y, Sacks FM, Bennett DA, Aggarwal NT. MIND diet associated with reduced incidence of Alzheimer's disease. Alzheimers Dement. 2015;11:1007-14.

[219] Olsson E, Karlström B, Kilander L, Byberg L, Cederholm T, Sjögren P. Dietary patterns and cognitive dysfunction in a 12-year follow-up study of 70 year old men. J Alzheimers Dis. 2015;43:109-19.

[220] Qin B, Adair LS, Plassman BL, Batis C, Edwards LJ, Popkin BM, et al. Dietary Patterns and Cognitive Decline Among Chinese Older Adults. Epidemiology. 2015;26:758-68.

[221] Scarmeas N, Stern Y, Mayeux R, Manly JJ, Schupf N, Luchsinger JA. Mediterranean diet and mild cognitive impairment. Arch Neurol. 2009;66:216-25.

[222] Tanaka T, Talegawkar SA, Jin Y, Colpo M, Ferrucci L, Bandinelli S. Adherence to a Mediterranean Diet Protects from Cognitive Decline in the Invecchiare in Chianti Study of Aging. Nutrients. 2018;10.

[223] Tangney CC, Kwasny MJ, Li H, Wilson RS, Evans DA, Morris MC. Adherence to a Mediterranean-type dietary pattern and cognitive decline in a community population. Am J Clin Nutr. 2011;93:601-7.

[224] Tangney CC, Li H, Wang Y, Barnes L, Schneider JA, Bennett DA, et al. Relation of DASH- and Mediterranean-like dietary patterns to cognitive decline in older persons. Neurology. 2014;83:1410-6.

[225] Trichopoulou A, Kyrozis A, Rossi M, Katsoulis M, Trichopoulos D, La Vecchia C, et al. Mediterranean diet and cognitive decline over time in an elderly Mediterranean population. Eur J Nutr. 2015;54:1311-21.

[226] Tsivgoulis G, Judd S, Letter AJ, Alexandrov AV, Howard G, Nahab F, et al. Adherence to a Mediterranean diet and risk of incident cognitive impairment. Neurology. 2013;80:1684-92.

[227] Chen R, Hu Z, Wei L, Ma Y, Liu Z, Copeland JR. Incident dementia in a defined older Chinese population. PLoS One. 2011;6:e24817.

[228] Eriksson UK, Bennet AM, Gatz M, Dickman PW, Pedersen NL. Nonstroke cardiovascular disease and risk of Alzheimer disease and dementia. Alzheimer Dis Assoc Disord. 2010;24:213-9.

[229] Haring B, Leng X, Robinson J, Johnson KC, Jackson RD, Beyth R, et al. Cardiovascular disease and cognitive decline in postmenopausal women: results from the Women's Health Initiative Memory Study. J Am Heart Assoc. 2013;2:e000369.

[230] Kahn S, Frishman WH, Weissman S, Ooi WL, Aronson M. Left ventricular hypertrophy on electrocardiogram: prognostic implications from a 10-year cohort study of older subjects: a report from the Bronx Longitudinal Aging Study. J Am Geriatr Soc. 1996;44:524-9.

[231] Lipnicki DM, Sachdev PS, Crawford J, Reppermund S, Kochan NA, Trollor JN, et al. Risk factors for late-life cognitive decline and variation with age and sex in the Sydney Memory and Ageing Study. PLoS One. 2013;8:e65841.

[232] Newman AB, Fitzpatrick AL, Lopez O, Jackson S, Lyketsos C, Jagust W, et al. Dementia and Alzheimer's disease incidence in relationship to cardiovascular disease in the Cardiovascular Health Study cohort. J Am Geriatr Soc. 2005;53:1101-7.

[233] Ross GW, Petrovitch H, White LR, Masaki KH, Li CY, Curb JD, et al. Characterization of risk factors for vascular dementia: the Honolulu-Asia Aging Study. Neurology. 1999;53:337-43.

[234] Ikram MA, van Oijen M, de Jong FJ, Kors JA, Koudstaal PJ, Hofman A, et al. Unrecognized myocardial infarction in relation to risk of dementia and cerebral small vessel disease. Stroke. 2008;39:1421-6.

[235] Kalmijn S, Feskens EJ, Launer LJ, Kromhout D. Cerebrovascular disease, the apolipoprotein e4 allele, and cognitive decline in a community-based study of elderly men. Stroke. 1996;27:2230-5.

[236] Qiu CX, Winblad B, Fratiglioni L. [Risk factors for dementia and Alzheimer' s disease-findings from a community-based cohort study in Stockholm, Sweden]. Zhonghua Liu Xing Bing Xue Za Zhi. 2005;26:882-7.

[237] Rusanen M, Kivipelto M, Levälahti E, Laatikainen T, Tuomilehto J, Soininen H, et al. Heart diseases and long-term risk of dementia and Alzheimer's disease: a population-based CAIDE study. J Alzheimers Dis. 2014;42:183-91.

[238] Satizabal C, Beiser AS, Seshadri S. Incidence of Dementia over Three Decades in the Framingham Heart Study. N Engl J Med. 2016;375:93-4.

[239] Aggarwal NT, Bienias JL, Bennett DA, Wilson RS, Morris MC, Schneider JA, et al. The relation of cigarette smoking to incident Alzheimer's disease in a biracial urban community population. Neuroepidemiology. 2006;26:140-6.

[240] Juan D, Zhou DH, Li J, Wang JY, Gao C, Chen M. A 2-year follow-up study of cigarette smoking and risk of dementia. Eur J Neurol. 2004;11:277-82.

[241] Luchsinger JA, Reitz C, Honig LS, Tang MX, Shea S, Mayeux R. Aggregation of vascular risk factors and risk of incident Alzheimer disease. Neurology. 2005;65:545-51.

[242] Moffat SD, Zonderman AB, Metter EJ, Kawas C, Blackman MR, Harman SM, et al. Free testosterone and risk for Alzheimer disease in older men. Neurology. 2004;62:188-93.

[243] Sabia S, Nabi H, Kivimaki M, Shipley MJ, Marmot MG, Singh-Manoux A. Health behaviors from early to late midlife as predictors of cognitive function: The Whitehall II study. Am J Epidemiol. 2009;170:428-37.

[244] Chang CC, Zhao Y, Lee CW, Ganguli M. Smoking, death, and Alzheimer disease: a case of competing risks. Alzheimer Dis Assoc Disord. 2012;26:300-6.

[245] Ohara T, Ninomiya T, Hata J, Ozawa M, Yoshida D, Mukai N, et al. Midlife and Late-Life Smoking and Risk of Dementia in the Community: The Hisayama Study. J Am Geriatr Soc. 2015;63:2332-9.

[246] Reitz C, den Heijer T, van Duijn C, Hofman A, Breteler MM. Relation between smoking and risk of dementia and Alzheimer disease: the Rotterdam Study. Neurology. 2007;69:998-1005.

[247] Rusanen M, Rovio S, Ngandu T, Nissinen A, Tuomilehto J, Soininen H, et al. Midlife smoking, apolipoprotein E and risk of dementia and Alzheimer's disease: a population-based cardiovascular risk factors, aging and dementia study. Dement Geriatr Cogn Disord. 2010;30:277-84.

[248] Tyas SL, White LR, Petrovitch H, Webster Ross G, Foley DJ, Heimovitz HK, et al. Mid-life smoking and late-life dementia: the Honolulu-Asia Aging Study. Neurobiol Aging. 2003;24:589-96.

[249] Whitmer RA, Gunderson EP, Barrett-Connor E, Quesenberry CP, Jr., Yaffe K. Obesity in middle age and future risk of dementia: a 27 year longitudinal population based study. Bmj. 2005;330:1360.

[250] Xu WL, Atti AR, Gatz M, Pedersen NL, Johansson B, Fratiglioni L. Midlife overweight and obesity increase late-life dementia risk: a population-based twin study. Neurology. 2011;76:1568-74.

[251] Albanese E, Davis B, Jonsson PV, Chang M, Aspelund T, Garcia M, et al. Overweight and Obesity in Midlife and Brain Structure and Dementia 26 Years Later: The AGES-Reykjavik Study. Am J Epidemiol. 2015;181:672-9.

[252] Beydoun MA, Lhotsky A, Wang Y, Dal Forno G, An Y, Metter EJ, et al. Association of adiposity status and changes in early to mid-adulthood with incidence of Alzheimer's disease. Am J Epidemiol. 2008;168:1179-89.

[253] Fitzpatrick AL, Kuller LH, Lopez OL, Diehr P, O'Meara ES, Longstreth WT, Jr., et al. Midlife and late-life obesity and the risk of dementia: cardiovascular health study. Arch Neurol. 2009;66:336-42.

[254] Kim S, Kim Y, Park SM. Body Mass Index and Decline of Cognitive Function. PLoS One. 2016;11:e0148908.

[255] Kivipelto M, Ngandu T, Fratiglioni L, Viitanen M, Kåreholt I, Winblad B, et al. Obesity and vascular risk factors at midlife and the risk of dementia and Alzheimer disease. Arch Neurol. 2005;62:1556-60.

[256] Ravona-Springer R, Schnaider-Beeri M, Goldbourt U. Body weight variability in midlife and risk for dementia in old age. Neurology. 2013;80:1677-83.

[257] Tolppanen AM, Ngandu T, Kåreholt I, Laatikainen T, Rusanen M, Soininen H, et al. Midlife and late-life body mass index and late-life dementia: results from a prospective population-based cohort. J Alzheimers Dis. 2014;38:201-9.

[258] Kivipelto M, Helkala EL, Laakso MP, Hänninen T, Hallikainen M, Alhainen K, et al. Midlife vascular risk factors and Alzheimer's disease in later life: longitudinal, population based study. Bmj. 2001;322:1447-51.

[259] Notkola IL, Sulkava R, Pekkanen J, Erkinjuntti T, Ehnholm C, Kivinen P, et al. Serum total cholesterol, apolipoprotein E epsilon 4 allele, and Alzheimer's disease. Neuroepidemiology. 1998;17:14-20.

[260] Solomon A, Kivipelto M, Wolozin B, Zhou J, Whitmer RA. Midlife serum cholesterol and increased risk of Alzheimer's and vascular dementia three decades later. Dement Geriatr Cogn Disord. 2009;28:75-80.

[261] Toro P, Degen C, Pierer M, Gustafson D, Schröder J, Schönknecht P. Cholesterol in mild cognitive impairment and Alzheimer's disease in a birth cohort over 14 years. Eur Arch Psychiatry Clin Neurosci. 2014;264:485-92.

[262] Beydoun MA, Beason-Held LL, Kitner-Triolo MH, Beydoun HA, Ferrucci L, Resnick SM, et al. Statins and serum cholesterol's associations with incident dementia and mild cognitive impairment. J Epidemiol Community Health. 2011;65:949-57.

[263] Mielke MM, Zandi PP, Shao H, Waern M, Östling S, Guo X, et al. The 32-year relationship between cholesterol and dementia from midlife to late life. Neurology. 2010;75:1888-95.

[264] Barnes LL, Mendes de Leon CF, Wilson RS, Bienias JL, Evans DA. Social resources and cognitive decline in a population of older African Americans and whites. Neurology. 2004;63:2322-6.

[265] Bassuk SS, Glass TA, Berkman LF. Social disengagement and incident cognitive decline in community-dwelling elderly persons. Ann Intern Med. 1999;131:165-73.

[266] Béland F, Zunzunegui MV, Alvarado B, Otero A, Del Ser T. Trajectories of cognitive decline and social relations. J Gerontol B Psychol Sci Soc Sci. 2005;60:P320-p30.

[267] Ertel KA, Glymour MM, Berkman LF. Effects of social integration on preserving memory function in a nationally representative US elderly population. Am J Public Health. 2008;98:1215-20.

[268] James BD, Wilson RS, Barnes LL, Bennett DA. Late-life social activity and cognitive decline in old age. J Int Neuropsychol Soc. 2011;17:998-1005.

[269] Lee Y, Kim J, Back JH. The influence of multiple lifestyle behaviors on cognitive function in older persons living in the community. Prev Med. 2009;48:86-90.

[270] Saczynski JS, Pfeifer LA, Masaki K, Korf ES, Laurin D, White L, et al. The effect of social engagement on incident dementia: the Honolulu-Asia Aging Study. Am J Epidemiol. 2006;163:433-40.

[271] Shatenstein B, Ferland G, Belleville S, Gray-Donald K, Kergoat MJ, Morais J, et al. Diet quality and cognition among older adults from the NuAge study. Exp Gerontol. 2012;47:353-60.

[272] Small BJ, Dixon RA, McArdle JJ, Grimm KJ. Do changes in lifestyle engagement moderate cognitive decline in normal aging? Evidence from the Victoria Longitudinal Study. Neuropsychology. 2012;26:144-55.

[273] Van Ness PH, Kasl SV. Religion and cognitive dysfunction in an elderly cohort. J Gerontol B Psychol Sci Soc Sci. 2003;58:S21-9.

[274] Wilson RS, Krueger KR, Arnold SE, Schneider JA, Kelly JF, Barnes LL, et al. Loneliness and risk of Alzheimer disease. Arch Gen Psychiatry. 2007;64:234-40.

[275] Yen CH, Yeh CJ, Wang CC, Liao WC, Chen SC, Chen CC, et al. Determinants of cognitive impairment over time among the elderly in Taiwan: results of the national longitudinal study. Arch Gerontol Geriatr. 2010;50 Suppl 1:S53-7.

[276] Zunzunegui MV, Alvarado BE, Del Ser T, Otero A. Social networks, social integration, and social engagement determine cognitive decline in community-dwelling Spanish older adults. J Gerontol B Psychol Sci Soc Sci. 2003;58:S93-s100.

[277] Bosma H, van Boxtel MP, Ponds RW, Jelicic M, Houx P, Metsemakers J, et al. Engaged lifestyle and cognitive function in middle and old-aged, non-demented persons: a reciprocal association? Z Gerontol Geriatr. 2002;35:575-81.

[278] Fabrigoule C, Letenneur L, Dartigues JF, Zarrouk M, Commenges D, Barberger-Gateau P. Social and leisure activities and risk of dementia: a prospective longitudinal study. J Am Geriatr Soc. 1995;43:485-90.

[279] Ghisletta P, Bickel JF, Lövdén M. Does activity engagement protect against cognitive decline in old age? Methodological and analytical considerations. J Gerontol B Psychol Sci Soc Sci. 2006;61:P253-61.

[280] Gureje O, Ogunniyi A, Kola L, Abiona T. Incidence of and risk factors for dementia in the Ibadan study of aging. J Am Geriatr Soc. 2011;59:869-74.

[281] He YL, Zhang XK, Zhang MY. Psychosocial risk factors for Alzheimer's disease. Hong Kong Journal of Psychiatry. 2000;10:2-7.

[282] Hill TD, Burdette AM, Angel JL, Angel RJ. Religious attendance and cognitive functioning among older Mexican Americans. J Gerontol B Psychol Sci Soc Sci. 2006;61:P3-9.

[283] Seeman TE, Lusignolo TM, Albert M, Berkman L. Social relationships, social support, and patterns of cognitive aging in healthy, high-functioning older adults: MacArthur studies of successful aging. Health Psychol. 2001;20:243-55.

[284] Thomas PA. Gender, social engagement, and limitations in late life. Soc Sci Med. 2011;73:1428-35.

[285] Valenzuela M, Brayne C, Sachdev P, Wilcock G, Matthews F. Cognitive lifestyle and long-term risk of dementia and survival after diagnosis in a multicenter population-based cohort. Am J Epidemiol. 2011;173:1004-12.

[286] Cohen-Manheim I, Sinnreich R, Doniger GM, Simon ES, Pinchas-Mizrachi R, Kark JD. Fasting plasma glucose in young adults free of diabetes is associated with cognitive function in midlife. Eur J Public Health. 2018;28:496-503.

[287] Wang F, Zhao M, Han Z, Li D, Zhang S, Zhang Y, et al. Long-Term Subclinical Hyperglycemia and Hypoglycemia as Independent Risk Factors for Mild Cognitive Impairment in Elderly People. Tohoku J Exp Med. 2017;242:121-8.

[288] Christman AL, Matsushita K, Gottesman RF, Mosley T, Alonso A, Coresh J, et al. Glycated haemoglobin and cognitive decline: the Atherosclerosis Risk in Communities (ARIC) study. Diabetologia. 2011;54:1645-52.

[289] Forti P, Pisacane N, Rietti E, Lucicesare A, Olivelli V, Mariani E, et al. Metabolic syndrome and risk of dementia in older adults. J Am Geriatr Soc. 2010;58:487-92.

[290] Mehlig K, Lapidus L, Thelle DS, Waern M, Zetterberg H, Björkelund C, et al. Low fasting serum insulin and dementia in nondiabetic women followed for 34 years. Neurology. 2018;91:e427-e35.

[291] Rönnemaa E, Zethelius B, Sundelöf J, Sundström J, Degerman-Gunnarsson M, Berne C, et al. Impaired insulin secretion increases the risk of Alzheimer disease. Neurology. 2008;71:1065-71.

[292] Rönnemaa E, Zethelius B, Sundelöf J, Sundström J, Degerman-Gunnarsson M, Lannfelt L, et al. Glucose metabolism and the risk of Alzheimer's disease and dementia: a population-based 12 year follow-up study in 71-year-old men. Diabetologia. 2009;52:1504-10.

[293] Schrijvers EM, Witteman JC, Sijbrands EJ, Hofman A, Koudstaal PJ, Breteler MM. Insulin metabolism and the risk of Alzheimer disease: the Rotterdam Study. Neurology. 2010;75:1982-7.

[294] Solfrizzi V, Scafato E, Capurso C, D'Introno A, Colacicco AM, Frisardi V, et al. Metabolic syndrome and the risk of vascular dementia: the Italian Longitudinal Study on Ageing. J Neurol Neurosurg Psychiatry. 2010;81:433-40.

[295] Wang F, Luo J, Ding D, Zhao Q, Guo Q, Liang X, et al. Elevated Fasting Blood Glucose Level Increases the Risk of Cognitive Decline Among Older Adults with Diabetes Mellitus: The Shanghai Aging Study. J Alzheimers Dis. 2019;67:1255-65.

[296] Amieva H, Ouvrard C, Giulioli C, Meillon C, Rullier L, Dartigues JF. Self-Reported Hearing Loss, Hearing Aids, and Cognitive Decline in Elderly Adults: A 25-Year Study. J Am Geriatr Soc. 2015;63:2099-104.

[297] Davies HR, Cadar D, Herbert A, Orrell M, Steptoe A. Hearing Impairment and Incident Dementia: Findings from the English Longitudinal Study of Ageing. J Am Geriatr Soc. 2017;65:2074-81.

[298] Deal JA, Betz J, Yaffe K, Harris T, Purchase-Helzner E, Satterfield S, et al. Hearing Impairment and Incident Dementia and Cognitive Decline in Older Adults: The Health ABC Study. J Gerontol A Biol Sci Med Sci. 2017;72:703-9.

[299] Deal JA, Sharrett AR, Albert MS, Coresh J, Mosley TH, Knopman D, et al. Hearing impairment and cognitive decline: a pilot study conducted within the atherosclerosis risk in communities neurocognitive study. Am J Epidemiol. 2015;181:680-90.

[300] Fischer ME, Cruickshanks KJ, Schubert CR, Pinto AA, Carlsson CM, Klein BE, et al. Age-Related Sensory Impairments and Risk of Cognitive Impairment. J Am Geriatr Soc. 2016;64:1981-7.

[301] Gallacher J, Ilubaera V, Ben-Shlomo Y, Bayer A, Fish M, Babisch W, et al. Auditory threshold, phonologic demand, and incident dementia. Neurology. 2012;79:1583-90.

[302] Gates GA, Anderson ML, McCurry SM, Feeney MP, Larson EB. Central auditory dysfunction as a harbinger of Alzheimer dementia. Arch Otolaryngol Head Neck Surg. 2011;137:390-5.

[303] Gates GA, Cobb JL, Linn RT, Rees T, Wolf PA, D'Agostino RB. Central auditory dysfunction, cognitive dysfunction, and dementia in older people. Arch Otolaryngol Head Neck Surg. 1996;122:161-7.

[304] Golub JS, Luchsinger JA, Manly JJ, Stern Y, Mayeux R, Schupf N. Observed Hearing Loss and Incident Dementia in a Multiethnic Cohort. J Am Geriatr Soc. 2017;65:1691-7.

[305] Gurgel RK, Ward PD, Schwartz S, Norton MC, Foster NL, Tschanz JT. Relationship of hearing loss and dementia: a prospective, population-based study. Otol Neurotol. 2014;35:775-81.

[306] Heywood R, Gao Q, Nyunt MSZ, Feng L, Chong MS, Lim WS, et al. Hearing Loss and Risk of Mild Cognitive Impairment and Dementia: Findings from the Singapore Longitudinal Ageing Study. Dement Geriatr Cogn Disord. 2017;43:259-68.

[307] Lin FR, Metter EJ, O'Brien RJ, Resnick SM, Zonderman AB, Ferrucci L. Hearing loss and incident dementia. Arch Neurol. 2011;68:214-20.

[308] Lin FR, Yaffe K, Xia J, Xue QL, Harris TB, Purchase-Helzner E, et al. Hearing loss and cognitive decline in older adults. JAMA Intern Med. 2013;173:293-9.

[309] Tomioka K, Okamoto N, Morikawa M, Kurumatani N. Self-Reported Hearing Loss Predicts 5-Year Decline in Higher-Level Functional Capacity in High-Functioning Elderly Adults: The Fujiwara-Kyo Study. J Am Geriatr Soc. 2015;63:2260-8.

[310] Anstey KJ, Luszcz MA, Sanchez L. Two-year decline in vision but not hearing is associated with memory decline in very old adults in a population-based sample. Gerontology. 2001;47:289-93.

[311] Hong T, Mitchell P, Burlutsky G, Liew G, Wang JJ. Visual Impairment, Hearing Loss and Cognitive Function in an Older Population: Longitudinal Findings from the Blue Mountains Eye Study. PLoS One. 2016;11:e0147646.

[312] Lin MY, Gutierrez PR, Stone KL, Yaffe K, Ensrud KE, Fink HA, et al. Vision impairment and combined vision and hearing impairment predict cognitive and functional decline in older women. J Am Geriatr Soc. 2004;52:1996-2002.

[313] Benito-León J, Bermejo-Pareja F, Vega S, Louis ED. Total daily sleep duration and the risk of dementia: a prospective population-based study. Eur J Neurol. 2009;16:990-7.

[314] Loerbroks A, Debling D, Amelang M, Stürmer T. Nocturnal sleep duration and cognitive impairment in a population-based study of older adults. Int J Geriatr Psychiatry. 2010;25:100-9.

[315] Ohara T, Honda T, Hata J, Yoshida D, Mukai N, Hirakawa Y, et al. Association Between Daily Sleep Duration and Risk of Dementia and Mortality in a Japanese Community. J Am Geriatr Soc. 2018;66:1911-8.

[316] Sindi S, Kåreholt I, Johansson L, Skoog J, Sjöberg L, Wang HX, et al. Sleep disturbances and dementia risk: A multicenter study. Alzheimers Dement. 2018;14:1235-42.

[317] Virta JJ, Heikkilä K, Perola M, Koskenvuo M, Räihä I, Rinne JO, et al. Midlife sleep characteristics associated with late life cognitive function. Sleep. 2013;36:1533-41, 41a.

[318] Westwood AJ, Beiser A, Jain N, Himali JJ, DeCarli C, Auerbach SH, et al. Prolonged sleep duration as a marker of early neurodegeneration predicting incident dementia. Neurology. 2017;88:1172-9.

[319] Blackwell T, Yaffe K, Laffan A, Ancoli-Israel S, Redline S, Ensrud KE, et al. Associations of objectively and subjectively measured sleep quality with subsequent cognitive decline in older community-dwelling men: the MrOS sleep study. Sleep. 2014;37:655-63.

[320] Chen JC, Espeland MA, Brunner RL, Lovato LC, Wallace RB, Leng X, et al. Sleep duration, cognitive decline, and dementia risk in older women. Alzheimers Dement. 2016;12:21-33.

[321] Diem SJ, Blackwell TL, Stone KL, Yaffe K, Tranah G, Cauley JA, et al. Measures of Sleep-Wake Patterns and Risk of Mild Cognitive Impairment or Dementia in Older Women. Am J Geriatr Psychiatry. 2016;24:248-58.

[322] Keage HA, Banks S, Yang KL, Morgan K, Brayne C, Matthews FE. What sleep characteristics predict cognitive decline in the elderly? Sleep Med. 2012;13:886-92.

[323] Lu Y, Sugawara Y, Zhang S, Tomata Y, Tsuji I. Changes in sleep duration and the risk of incident dementia in the elderly Japanese: the Ohsaki Cohort 2006 Study. Sleep. 2018;41.

[324] Lutsey PL, Misialek JR, Mosley TH, Gottesman RF, Punjabi NM, Shahar E, et al. Sleep characteristics and risk of dementia and Alzheimer's disease: The Atherosclerosis Risk in Communities Study. Alzheimers Dement. 2018;14:157-66.

[325] Ramos AR, Gardener H, Rundek T, Elkind MS, Boden-Albala B, Dong C, et al. Sleep disturbances and cognitive decline in the Northern Manhattan Study. Neurology. 2016;87:1511-6.

[326] Tworoger SS, Lee S, Schernhammer ES, Grodstein F. The association of self-reported sleep duration, difficulty sleeping, and snoring with cognitive function in older women. Alzheimer Dis Assoc Disord. 2006;20:41-8.

[327] Xu L, Jiang CQ, Lam TH, Zhang WS, Cherny SS, Thomas GN, et al. Sleep duration and memory in the elderly Chinese: longitudinal analysis of the Guangzhou Biobank Cohort Study. Sleep. 2014;37:1737-44.

[328] Dearborn PJ, Elias MF, Sullivan KJ, Sullivan CE, Robbins MA. Poorer Visual Acuity Is Associated with Declines in Cognitive Performance Across Multiple Cognitive Domains: The Maine-Syracuse Longitudinal Study. J Int Neuropsychol Soc. 2018;24:746-54.

[329] Hwang PH, Longstreth WT, Jr., Brenowitz WD, Thielke SM, Lopez OL, Francis CE, et al. Dual sensory impairment in older adults and risk of dementia from the GEM Study. Alzheimers Dement (Amst). 2020;12:e12054.

[330] Lee ATC, Richards M, Chan WC, Chiu HFK, Lee RSY, Lam LCW. Higher Dementia Incidence in Older Adults with Poor Visual Acuity. J Gerontol A Biol Sci Med Sci. 2020;75:2162-8.

[331] Luck T, Riedel-Heller SG, Luppa M, Wiese B, Wollny A, Wagner M, et al. Risk factors for incident mild cognitive impairment--results from the German Study on Ageing, Cognition and Dementia in Primary Care Patients (AgeCoDe). Acta Psychiatr Scand. 2010;121:260-72.

[332] Maharani A, Dawes P, Nazroo J, Tampubolon G, Pendleton N. Associations Between Self-Reported Sensory Impairment and Risk of Cognitive Decline and Impairment in the Health and Retirement Study Cohort. J Gerontol B Psychol Sci Soc Sci. 2020;75:1230-42.

[333] Rogers MA, Langa KM. Untreated poor vision: a contributing factor to late-life dementia. Am J Epidemiol. 2010;171:728-35.

[334] Tran EM, Stefanick ML, Henderson VW, Rapp SR, Chen JC, Armstrong NM, et al. Association of Visual Impairment With Risk of Incident Dementia in a Women's Health Initiative Population. JAMA Ophthalmol. 2020;138:624-33.

[335] Ward ME, Gelfand JM, Lui LY, Ou Y, Green AJ, Stone K, et al. Reduced contrast sensitivity among older women is associated with increased risk of cognitive impairment. Ann Neurol. 2018;83:730-8.

[336] Naël V, Pérès K, Dartigues JF, Letenneur L, Amieva H, Arleo A, et al. Vision loss and 12-year risk of dementia in older adults: the 3C cohort study. Eur J Epidemiol. 2019;34:141-52.

[337] Brenowitz WD, Kaup AR, Lin FR, Yaffe K. Multiple Sensory Impairment Is Associated With Increased Risk of Dementia Among Black and White Older Adults. J Gerontol A Biol Sci Med Sci. 2019;74:890-6.

[338] Davies-Kershaw HR, Hackett RA, Cadar D, Herbert A, Orrell M, Steptoe A. Vision Impairment and Risk of Dementia: Findings from the English Longitudinal Study of Ageing. J Am Geriatr Soc. 2018;66:1823-9.

[339] Lipnicki DM, Crawford J, Kochan NA, Trollor JN, Draper B, Reppermund S, et al. Risk Factors for Mild Cognitive Impairment, Dementia and Mortality: The Sydney Memory and Ageing Study. J Am Med Dir Assoc. 2017;18:388-95.

[340] Bailey MJ, Soliman EZ, McClure LA, Howard G, Howard VJ, Judd SE, et al. Relation of Atrial Fibrillation to Cognitive Decline (from the REasons for Geographic and Racial Differences in Stroke [REGARDS] Study). Am J Cardiol. 2021;148:60-8.

[341] Chen LY, Norby FL, Gottesman RF, Mosley TH, Soliman EZ, Agarwal SK, et al. Association of Atrial Fibrillation With Cognitive Decline and Dementia Over 20 Years: The ARIC-NCS (Atherosclerosis Risk in Communities Neurocognitive Study). J Am Heart Assoc. 2018;7.

[342] de Bruijn RF, Heeringa J, Wolters FJ, Franco OH, Stricker BH, Hofman A, et al. Association Between Atrial Fibrillation and Dementia in the General Population. JAMA Neurol. 2015;72:1288-94.

[343] Ding M, Fratiglioni L, Johnell K, Santoni G, Fastbom J, Ljungman P, et al. Atrial fibrillation, antithrombotic treatment, and cognitive aging: A population-based study. Neurology. 2018;91:e1732-e40.

[344] Dublin S, Anderson ML, Haneuse SJ, Heckbert SR, Crane PK, Breitner JC, et al. Atrial fibrillation and risk of dementia: a prospective cohort study. J Am Geriatr Soc. 2011;59:1369-75.

[345] Elias MF, Sullivan LM, Elias PK, Vasan RS, D'Agostino RB, Sr., Seshadri S, et al. Atrial fibrillation is associated with lower cognitive performance in the Framingham offspring men. J Stroke Cerebrovasc Dis. 2006;15:214-22.

[346] Marengoni A, Qiu C, Winblad B, Fratiglioni L. Atrial fibrillation, stroke and dementia in the very old: a population-based study. Neurobiol Aging. 2011;32:1336-7.

[347] Rydén L, Zettergren A, Seidu NM, Guo X, Kern S, Blennow K, et al. Atrial fibrillation increases the risk of dementia amongst older adults even in the absence of stroke. J Intern Med. 2019;286:101-10.

[348] Singh-Manoux A, Fayosse A, Sabia S, Canonico M, Bobak M, Elbaz A, et al. Atrial fibrillation as a risk factor for cognitive decline and dementia. Eur Heart J. 2017;38:2612-8.

[349] Forti P, Maioli F, Pisacane N, Rietti E, Montesi F, Ravaglia G. Atrial fibrillation and risk of dementia in non-demented elderly subjects with and without mild cognitive impairment (MCI). Arch Gerontol Geriatr. 2007;44 Suppl 1:155-65.

[350] Rastas S, Verkkoniemi A, Polvikoski T, Juva K, Niinistö L, Mattila K, et al. Atrial fibrillation, stroke, and cognition: a longitudinal population-based study of people aged 85 and older. Stroke. 2007;38:1454-60.

[351] Burke SL, Maramaldi P, Cadet T, Kukull W. Neuropsychiatric symptoms and Apolipoprotein E: Associations with eventual Alzheimer's disease development. Arch Gerontol Geriatr. 2016;65:231-8.

[352] Kassem AM, Ganguli M, Yaffe K, Hanlon JT, Lopez OL, Wilson JW, et al. Anxiety symptoms and risk of dementia and mild cognitive impairment in the oldest old women. Aging Ment Health. 2018;22:474-82.

[353] Petkus AJ, Reynolds CA, Wetherell JL, Kremen WS, Pedersen NL, Gatz M. Anxiety is associated with increased risk of dementia in older Swedish twins. Alzheimers Dement. 2016;12:399-406.

[354] Santabárbara J, Lopez-Anton R, de la Cámara C, Lobo E, Gracia-García P, Villagrasa B, et al. Clinically significant anxiety as a risk factor for dementia in the elderly community. Acta Psychiatr Scand. 2019;139:6-14.

[355] Sutin AR, Stephan Y, Terracciano A. Psychological Distress, Self-Beliefs, and Risk of Cognitive Impairment and Dementia. J Alzheimers Dis. 2018;65:1041-50.

[356] Terracciano A, Sutin AR, An Y, O'Brien RJ, Ferrucci L, Zonderman AB, et al. Personality and risk of Alzheimer's disease: new data and meta-analysis. Alzheimers Dement. 2014;10:179-86.

[357] de Bruijn RF, Direk N, Mirza SS, Hofman A, Koudstaal PJ, Tiemeier H, et al. Anxiety is not associated with the risk of dementia or cognitive decline: the Rotterdam Study. Am J Geriatr Psychiatry. 2014;22:1382-90.

[358] Gallacher J, Bayer A, Fish M, Pickering J, Pedro S, Dunstan F, et al. Does anxiety affect risk of dementia? Findings from the Caerphilly Prospective Study. Psychosom Med. 2009;71:659-66.

[359] Mortamais M, Abdennour M, Bergua V, Tzourio C, Berr C, Gabelle A, et al. Anxiety and 10-Year Risk of Incident Dementia-An Association Shaped by Depressive Symptoms: Results of the Prospective Three-City Study. Front Neurosci. 2018;12:248.

[360] Barnes DE, Cauley JA, Lui LY, Fink HA, McCulloch C, Stone KL, et al. Women who maintain optimal cognitive function into old age. J Am Geriatr Soc. 2007;55:259-64.

[361] Holtzman RE, Rebok GW, Saczynski JS, Kouzis AC, Wilcox Doyle K, Eaton WW. Social network characteristics and cognition in middle-aged and older adults. J Gerontol B Psychol Sci Soc Sci. 2004;59:P278-84.

[362] Albert MS, Jones K, Savage CR, Berkman L, Seeman T, Blazer D, et al. Predictors of cognitive change in older persons: MacArthur studies of successful aging. Psychol Aging. 1995;10:578-89.

[363] Amieva H, Stoykova R, Matharan F, Helmer C, Antonucci TC, Dartigues JF. What aspects of social network are protective for dementia? Not the quantity but the quality of social interactions is protective up to 15 years later. Psychosom Med. 2010;72:905-11.

[364] Glei DA, Landau DA, Goldman N, Chuang YL, Rodríguez G, Weinstein M. Participating in social activities helps preserve cognitive function: an analysis of a longitudinal, population-based study of the elderly. Int J Epidemiol. 2005;34:864-71.

[365] Green AF, Rebok G, Lyketsos CG. Influence of social network characteristics on cognition and functional status with aging. Int J Geriatr Psychiatry. 2008;23:972-8.

[366] Hughes TF, Andel R, Small BJ, Borenstein AR, Mortimer JA. The association between social resources and cognitive change in older adults: evidence from the Charlotte County Healthy Aging Study. J Gerontol B Psychol Sci Soc Sci. 2008;63:P241-p4.

[367] Monastero R, Palmer K, Qiu C, Winblad B, Fratiglioni L. Heterogeneity in risk factors for cognitive impairment, no dementia: population-based longitudinal study from the Kungsholmen Project. Am J Geriatr Psychiatry. 2007;15:60-9.

[368] Stoykova R, Matharan F, Dartigues JF, Amieva H. Impact of social network on cognitive performances and age-related cognitive decline across a 20-year follow-up. Int Psychogeriatr. 2011;23:1405-12.

[369] Holwerda TJ, Deeg DJ, Beekman AT, van Tilburg TG, Stek ML, Jonker C, et al. Feelings of loneliness, but not social isolation, predict dementia onset: results from the Amsterdam Study of the Elderly (AMSTEL). J Neurol Neurosurg Psychiatry. 2014;85:135-42.

[370] Lobo A, López-Antón R, de-la-Cámara C, Quintanilla MA, Campayo A, Saz P. Non-cognitive psychopathological symptoms associated with incident mild cognitive impairment and dementia, Alzheimer's type. Neurotox Res. 2008;14:263-72.

[371] Rafnsson SB, Orrell M, d'Orsi E, Hogervorst E, Steptoe A. Loneliness, Social Integration, and Incident Dementia Over 6 Years: Prospective Findings From the English Longitudinal Study of Ageing. J Gerontol B Psychol Sci Soc Sci. 2020;75:114-24.

[372] Sutin AR, Stephan Y, Luchetti M, Terracciano A. Loneliness and Risk of Dementia. J Gerontol B Psychol Sci Soc Sci. 2020;75:1414-22.

[373] Zhou Z, Wang P, Fang Y. Loneliness and the risk of dementia among older Chinese adults: gender differences. Aging Ment Health. 2018;22:519-25.

[374] Rawtaer I, Gao Q, Nyunt MS, Feng L, Chong MS, Lim WS, et al. Psychosocial Risk and Protective Factors and Incident Mild Cognitive Impairment and Dementia in Community Dwelling Elderly: Findings from the Singapore Longitudinal Ageing Study. J Alzheimers Dis. 2017;57:603-11.

[375] Komiyama T, Ohi T, Miyoshi Y, Murakami T, Tsuboi A, Tomata Y, et al. Association Between Tooth Loss, Receipt of Dental Care, and Functional Disability in an Elderly Japanese Population: The Tsurugaya Project. J Am Geriatr Soc. 2016;64:2495-502.

[376] Luo J, Wu B, Zhao Q, Guo Q, Meng H, Yu L, et al. Association between tooth loss and cognitive function among 3063 Chinese older adults: a community-based study. PLoS One. 2015;10:e0120986.

[377] Minn YK, Suk SH, Park H, Cheong JS, Yang H, Lee S, et al. Tooth loss is associated with brain white matter change and silent infarction among adults without dementia and stroke. J Korean Med Sci. 2013;28:929-33.

[378] Naorungroj S, Schoenbach VJ, Wruck L, Mosley TH, Gottesman RF, Alonso A, et al. Tooth loss, periodontal disease, and cognitive decline in the Atherosclerosis Risk in Communities (ARIC) study. Community Dent Oral Epidemiol. 2015;43:47-57.

[379] Nilsson H, Berglund JS, Renvert S. Periodontitis, tooth loss and cognitive functions among older adults. Clin Oral Investig. 2018;22:2103-9.

[380] Okamoto N, Morikawa M, Tomioka K, Yanagi M, Amano N, Kurumatani N. Association between tooth loss and the development of mild memory impairment in the elderly: the Fujiwara-kyo Study. J Alzheimers Dis. 2015;44:777-86.

[381] Yamamoto T, Kondo K, Hirai H, Nakade M, Aida J, Hirata Y. Association between self-reported dental health status and onset of dementia: a 4-year prospective cohort study of older Japanese adults from the Aichi Gerontological Evaluation Study (AGES) Project. Psychosom Med. 2012;74:241-8.

[382] Paganini-Hill A, White SC, Atchison KA. Dentition, dental health habits, and dementia: the Leisure World Cohort Study. J Am Geriatr Soc. 2012;60:1556-63.

[383] Stewart R, Stenman U, Hakeberg M, Hägglin C, Gustafson D, Skoog I. Associations between oral health and risk of dementia in a 37-year follow-up study: the prospective population study of women in Gothenburg. J Am Geriatr Soc. 2015;63:100-5.

[384] Takeuchi K, Ohara T, Furuta M, Takeshita T, Shibata Y, Hata J, et al. Tooth Loss and Risk of Dementia in the Community: the Hisayama Study. J Am Geriatr Soc. 2017;65:e95-e100.

[385] Jelicic M, Bosma H, Ponds RW, Van Boxtel MP, Houx PJ, Jolles J. Subjective sleep problems in later life as predictors of cognitive decline. Report from the Maastricht Ageing Study (MAAS). Int J Geriatr Psychiatry. 2002;17:73-7.

[386] Lim AS, Kowgier M, Yu L, Buchman AS, Bennett DA. Sleep Fragmentation and the Risk of Incident Alzheimer's Disease and Cognitive Decline in Older Persons. Sleep. 2013;36:1027-32.

[387] Luojus MK, Lehto SM, Tolmunen T, Brem AK, Lönnroos E, Kauhanen J. Self-reported sleep disturbance and incidence of dementia in ageing men. J Epidemiol Community Health. 2017;71:329-35.

[388] Bokenberger K, Ström P, Dahl Aslan AK, Johansson AL, Gatz M, Pedersen NL, et al. Association Between Sleep Characteristics and Incident Dementia Accounting for Baseline Cognitive Status: A Prospective Population-Based Study. J Gerontol A Biol Sci Med Sci. 2017;72:134-9.

[389] Hahn EA, Wang HX, Andel R, Fratiglioni L. A change in sleep pattern may predict Alzheimer disease. Am J Geriatr Psychiatry. 2014;22:1262-71.

[390] Cremer A, Soumaré A, Berr C, Dartigues JF, Gabelle A, Gosse P, et al. Orthostatic Hypotension and Risk of Incident Dementia: Results From a 12-Year Follow-Up of the Three-City Study Cohort. Hypertension. 2017;70:44-9.

[391] Elmståhl S, Widerström E. Orthostatic intolerance predicts mild cognitive impairment: incidence of mild cognitive impairment and dementia from the Swedish general population cohort Good Aging in Skåne. Clin Interv Aging. 2014;9:1993-2002.

[392] Rawlings AM, Juraschek SP, Heiss G, Hughes T, Meyer ML, Selvin E, et al. Association of orthostatic hypotension with incident dementia, stroke, and cognitive decline. Neurology. 2018;91:e759-e68.

[393] Wolters FJ, Mattace-Raso FU, Koudstaal PJ, Hofman A, Ikram MA. Orthostatic Hypotension and the Long-Term Risk of Dementia: A Population-Based Study. PLoS Med. 2016;13:e1002143.

[394] Curreri C, Giantin V, Veronese N, Trevisan C, Sartori L, Musacchio E, et al. Orthostatic Changes in Blood Pressure and Cognitive Status in the Elderly: The Progetto Veneto Anziani Study. Hypertension. 2016;68:427-35.

[395] Feeney J, O'Leary N, Kenny RA. Erratum to: Impaired orthostatic blood pressure recovery and cognitive performance at two-year follow up in older adults: The Irish Longitudinal Study on Ageing. Clin Auton Res. 2016;26:243.

[396] McNicholas T, Tobin K, Carey D, O'Callaghan S, Kenny RA. Is Baseline Orthostatic Hypotension Associated With a Decline in Global Cognitive Performance at 4-Year Follow-Up? Data From TILDA (The Irish Longitudinal Study on Ageing). J Am Heart Assoc. 2018;7:e008976.

[397] Peters R, Anstey KJ, Booth A, Beckett N, Warwick J, Antikainen R, et al. Orthostatic hypotension and symptomatic subclinical orthostatic hypotension increase risk of cognitive impairment: an integrated evidence review and analysis of a large older adult hypertensive cohort. Eur Heart J. 2018;39:3135-43.

[398] Shah RC, Wilson RS, Bienias JL, Arvanitakis Z, Evans DA, Bennett DA. Relation of blood pressure to risk of incident Alzheimer's disease and change in global cognitive function in older persons. Neuroepidemiology. 2006;26:30-6.

[399] Fratiglioni L, Wang HX, Ericsson K, Maytan M, Winblad B. Influence of social network on occurrence of dementia: a community-based longitudinal study. Lancet. 2000;355:1315-9.

[400] Arai A, Katsumata Y, Konno K, Tamashiro H. Sociodemographic factors associated with incidence of dementia among senior citizens of a small town in Japan. Care Manag J. 2004;5:159-65.

[401] Bickel H, Cooper B. Incidence and relative risk of dementia in an urban elderly population: findings of a prospective field study. Psychol Med. 1994;24:179-92.

[402] Helmer C, Damon D, Letenneur L, Fabrigoule C, Barberger-Gateau P, Lafont S, et al. Marital status and risk of Alzheimer's disease: a French population-based cohort study. Neurology. 1999;53:1953-8.

[403] Sörman DE, Rönnlund M, Sundström A, Adolfsson R, Nilsson LG. Social relationships and risk of dementia: a population-based study. Int Psychogeriatr. 2015;27:1391-9.

[404] Rodriguez FS, Pabst A, Luck T, König HH, Angermeyer MC, Witte AV, et al. Social Network Types in Old Age and Incident Dementia. J Geriatr Psychiatry Neurol. 2018;31:163-70.

[405] Adams DR, Kern DW, Wroblewski KE, McClintock MK, Dale W, Pinto JM. Olfactory Dysfunction Predicts Subsequent Dementia in Older U.S. Adults. J Am Geriatr Soc. 2018;66:140-4.

[406] Devanand DP, Lee S, Manly J, Andrews H, Schupf N, Doty RL, et al. Olfactory deficits predict cognitive decline and Alzheimer dementia in an urban community. Neurology. 2015;84:182-9.

[407] Graves AB, Bowen JD, Rajaram L, McCormick WC, McCurry SM, Schellenberg GD, et al. Impaired olfaction as a marker for cognitive decline: interaction with apolipoprotein E epsilon4 status. Neurology. 1999;53:1480-7.

[408] Roberts RO, Christianson TJ, Kremers WK, Mielke MM, Machulda MM, Vassilaki M, et al. Association Between Olfactory Dysfunction and Amnestic Mild Cognitive Impairment and Alzheimer Disease Dementia. JAMA Neurol. 2016;73:93-101.

[409] Sohrabi HR, Bates KA, Weinborn MG, Johnston AN, Bahramian A, Taddei K, et al. Olfactory discrimination predicts cognitive decline among community-dwelling older adults. Transl Psychiatry. 2012;2:e118.

[410] Wilson RS, Arnold SE, Schneider JA, Boyle PA, Buchman AS, Bennett DA. Olfactory impairment in presymptomatic Alzheimer's disease. Ann N Y Acad Sci. 2009;1170:730-5.

[411] Yaffe K, Freimer D, Chen H, Asao K, Rosso A, Rubin S, et al. Olfaction and risk of dementia in a biracial cohort of older adults. Neurology. 2017;88:456-62.

[412] Benedict C, Byberg L, Cedernaes J, Hogenkamp PS, Giedratis V, Kilander L, et al. Self-reported sleep disturbance is associated with Alzheimer's disease risk in men. Alzheimers Dement. 2015;11:1090-7.

[413] Cricco M, Simonsick EM, Foley DJ. The impact of insomnia on cognitive functioning in older adults. J Am Geriatr Soc. 2001;49:1185-9.

[414] Osorio RS, Pirraglia E, Agüera-Ortiz LF, During EH, Sacks H, Ayappa I, et al. Greater risk of Alzheimer's disease in older adults with insomnia. J Am Geriatr Soc. 2011;59:559-62.

[415] Elwood PC, Bayer AJ, Fish M, Pickering J, Mitchell C, Gallacher JE. Sleep disturbance and daytime sleepiness predict vascular dementia. J Epidemiol Community Health. 2011;65:820-4.

[416] Foley D, Monjan A, Masaki K, Ross W, Havlik R, White L, et al. Daytime sleepiness is associated with 3-year incident dementia and cognitive decline in older Japanese-American men. J Am Geriatr Soc. 2001;49:1628-32.

[417] Morgan K, Lilley JM. Risk factors among incident cases of dementia in a representative british sample. International Journal of Geriatric Psychiatry. 1994;9:11-5.

[418] Tsapanou A, Gu Y, Manly J, Schupf N, Tang MX, Zimmerman M, et al. Daytime Sleepiness and Sleep Inadequacy as Risk Factors for Dementia. Dement Geriatr Cogn Dis Extra. 2015;5:286-95.

[419] Jaussent I, Bouyer J, Ancelin ML, Berr C, Foubert-Samier A, Ritchie K, et al. Excessive sleepiness is predictive of cognitive decline in the elderly. Sleep. 2012;35:1201-7.

[420] Raffaitin C, Féart C, Le Goff M, Amieva H, Helmer C, Akbaraly TN, et al. Metabolic syndrome and cognitive decline in French elders: the Three-City Study. Neurology. 2011;76:518-25.

[421] Yaffe K, Haan M, Blackwell T, Cherkasova E, Whitmer RA, West N. Metabolic syndrome and cognitive decline in elderly Latinos: findings from the Sacramento Area Latino Study of Aging study. J Am Geriatr Soc. 2007;55:758-62.

[422] Yaffe K, Kanaya A, Lindquist K, Simonsick EM, Harris T, Shorr RI, et al. The metabolic syndrome, inflammation, and risk of cognitive decline. Jama. 2004;292:2237-42.

[423] Harrison SL, Stephan BC, Siervo M, Granic A, Davies K, Wesnes KA, et al. Is there an association between metabolic syndrome and cognitive function in very old adults? The Newcastle 85+ Study. J Am Geriatr Soc. 2015;63:667-75.

[424] Ritchie SJ, Gow AJ, Deary IJ. Religiosity is negatively associated with later-life intelligence, but not with age-related cognitive decline. Intelligence. 2014;46:9-17.

[425] Yeager DM, Glei DA, Au M, Lin HS, Sloan RP, Weinstein M. Religious involvement and health outcomes among older persons in Taiwan. Soc Sci Med. 2006;63:2228-41.

[426] Choi Y, Park S, Cho KH, Chun SY, Park EC. A change in social activity affect cognitive function in middle-aged and older Koreans: analysis of a Korean longitudinal study on aging (2006-2012). Int J Geriatr Psychiatry. 2016;31:912-9.

[427] Hjelm C, Dahl A, Broström A, Mårtensson J, Johansson B, Strömberg A. The influence of heart failure on longitudinal changes in cognition among individuals 80 years of age and older. J Clin Nurs. 2012;21:994-1003.

[428] Qiu C, Winblad B, Marengoni A, Klarin I, Fastbom J, Fratiglioni L. Heart failure and risk of dementia and Alzheimer disease: a population-based cohort study. Arch Intern Med. 2006;166:1003-8.

[429] Noale M, Limongi F, Zambon S, Crepaldi G, Maggi S. Incidence of dementia: evidence for an effect modification by gender. The ILSA Study. Int Psychogeriatr. 2013;25:1867-76.

[430] Martin MS, Sforza E, Roche F, Barthélémy JC, Thomas-Anterion C. Sleep breathing disorders and cognitive function in the elderly: an 8-year follow-up study. the proof-synapse cohort. Sleep. 2015;38:179-87.

[431] Yaffe K, Laffan AM, Harrison SL, Redline S, Spira AP, Ensrud KE, et al. Sleep-disordered breathing, hypoxia, and risk of mild cognitive impairment and dementia in older women. Jama. 2011;306:613-9.

[432] Blackwell T, Yaffe K, Laffan A, Redline S, Ancoli-Israel S, Ensrud KE, et al. Associations between sleep-disordered breathing, nocturnal hypoxemia, and subsequent cognitive decline in older community-dwelling men: the Osteoporotic Fractures in Men Sleep Study. J Am Geriatr Soc. 2015;63:453-61.

[433] Lutsey PL, Bengtson LG, Punjabi NM, Shahar E, Mosley TH, Gottesman RF, et al. Obstructive Sleep Apnea and 15-Year Cognitive Decline: The Atherosclerosis Risk in Communities (ARIC) Study. Sleep. 2016;39:309-16.

[434] Stewart R, Weyant RJ, Garcia ME, Harris T, Launer LJ, Satterfield S, et al. Adverse oral health and cognitive decline: the health, aging and body composition study. J Am Geriatr Soc. 2013;61:177-84.

[435] Arrivé E, Letenneur L, Matharan F, Laporte C, Helmer C, Barberger-Gateau P, et al. Oral health condition of French elderly and risk of dementia: a longitudinal cohort study. Community Dent Oral Epidemiol. 2012;40:230-8.

[436] Islamoska S, Ishtiak-Ahmed K, Hansen Å M, Grynderup MB, Mortensen EL, Garde AH, et al. Vital Exhaustion and Incidence of Dementia: Results from the Copenhagen City Heart Study. J Alzheimers Dis. 2019;67:369-79.

[437] Johansson L, Guo X, Waern M, Ostling S, Gustafson D, Bengtsson C, et al. Midlife psychological stress and risk of dementia: a 35-year longitudinal population study. Brain. 2010;133:2217-24.

[438] Wilson RS, Barnes LL, Bennett DA, Li Y, Bienias JL, Mendes de Leon CF, et al. Proneness to psychological distress and risk of Alzheimer disease in a biracial community. Neurology. 2005;64:380-2.

[439] Andrew MK, Rockwood K. Social vulnerability predicts cognitive decline in a prospective cohort of older Canadians. Alzheimers Dement. 2010;6:319-25.e1.

[440] Haug MR, Breslau N, Folmar SJ. Coping resources and selective survival in mental health of the elderly. Res Aging. 1989;11:468-91.

[441] Watfa G, Husson N, Buatois S, Laurain MC, Miget P, Benetos A. Study of Mini-Mental State Exam evolution in community-dwelling subjects aged over 60 years without dementia. J Nutr Health Aging. 2011;15:901-4.

[442] Ravaglia G, Forti P, Maioli F, Martelli M, Servadei L, Brunetti N, et al. Homocysteine and folate as risk factors for dementia and Alzheimer disease. Am J Clin Nutr. 2005;82:636-43.

[443] Middleton LE, Kirkland SA, Maxwell CJ, Hogan DB, Rockwood K. Exercise: a potential contributing factor to the relationship between folate and dementia. J Am Geriatr Soc. 2007;55:1095-8.

[444] Wang HX, Wahlin A, Basun H, Fastbom J, Winblad B, Fratiglioni L. Vitamin B(12) and folate in relation to the development of Alzheimer's disease. Neurology. 2001;56:1188-94.

[445] Hayden KM, Norton MC, Darcey D, Ostbye T, Zandi PP, Breitner JC, et al. Occupational exposure to pesticides increases the risk of incident AD: the Cache County study. Neurology. 2010;74:1524-30.

[446] Baldi I, Lebailly P, Mohammed-Brahim B, Letenneur L, Dartigues JF, Brochard P. Neurodegenerative diseases and exposure to pesticides in the elderly. Am J Epidemiol. 2003;157:409-14.
