# Supplementary material for: Umbrella review and Delphi study on modifiable factors for dementia risk reduction
Source: Alzheimers Dement. 2023 Dec 30;20(3):2223–39. doi: 10.1002/alz.13577 (PMC10984497; doi:10.1002/alz.13577)
Supplement: Supplementary file 9 — Supporting Information [file ALZ-20-2223-s007.docx]

**Appendix I: Full Results Delphi Round 1**

**Supplementary table 4**: Full results Delphi round 1, based on the input of 18 dementia experts.

| **Modifiable risk/protective factor** | **^*^Frequency** | ^†^**Ranks** | ^‡^**Rank score** |
| --- | --- | --- | --- |
| Hearing impairment | 11 | 1, 1, 1, 2, 2, 3, 3, 3, 4, 4, 8 | 761 |
| Social isolation/loneliness | 13 | 1, 2, 2, 2, 3, 3, 3, 3, 3, 4, 4, 5, 7 | 723 |
| Traumatic brain injury | 11 | 1, 1, 2, 2, 3, 3, 3, 4, 4, 5, 7 | 704 |
| Education | 8 | 1, 1, 2, 2, 2, 3, 4, 5 | 592 |
| Sleep | 8 | 1, 1, 1, 2, 2, 4, 5, 6 | 572 |
| Stroke/cerebrovascular disease | 5 | 1, 1, 1, 5, 8 | 345 |
| Air pollution | 7 | 1, 3, 4, 5, 5, 6, 7 | 326 |
| Psychological stress | 3 | 2, 3, 8 | 154 |
| Visual impairment | 4 | 3, 4, 5, 12 | 149 |
| Lifecourse inequalities | 1 | 1 | 100 |
| Atrial fibrillation | 3 | 4, 6, 8 | 83 |
| Delirium | 1 | 2 | 81 |
| Early brain development | 1 | 2 | 81 |
| Lung function | 1 | 2 | 81 |
| Vitamin D deficiency | 1 | 3 | 64 |
| High alcohol use | 1 | 4 | 49 |
| Mild behavioural impairment | 1 | 4 | 49 |
| Negative life events | 1 | 4 | 49 |
| Passive smoking | 1 | 4 | 49 |
| Quality of work | 1 | 4 | 49 |
| Impaired oral health | 1 | 5 | 36 |
| Substance use | 1 | 5 | 36 |
| Anemia | 1 | 6 | 25 |
| Orthostatic hypotension | 1 | 6 | 25 |
| Chronic inflammation | 1 | 9 | 4 |
| COVID-19 infection | 1 | 9 | 4 |
| Drugs | 1 | 13 | 0 |

NOTE. The experts were asked to list new modifiable risk and protective factors (not previously included in the LIBRA index) in order of subjective importance. The table provides an overview of these factors, ordered based on the factors’ rank scores.

**^*^**Frequency = The frequency that a particular factor was named
^†^Ranks = The ranks that were given to a particular factor
^‡^Rank score = The score of a particular factor based on the ranks given (see Appendix F for full calculations)
