# Supplementary material for: Umbrella review and Delphi study on modifiable factors for dementia risk reduction
Source: Alzheimers Dement. 2023 Dec 30;20(3):2223–39. doi: 10.1002/alz.13577 (PMC10984497; doi:10.1002/alz.13577)
Supplement: Supplementary file 10 — Supporting Information [file ALZ-20-2223-s004.docx]

**Appendix J: Full Results Delphi Round 2 Operationalisations**

**Supplementary table 5:** Full results for operationalisations of social contact in Delphi round 2, based on the input of 17 dementia experts.

Social contact

| **Operationalisation** | **Allocated points (mean)** | **Standard deviation** |
| --- | --- | --- |
| Low social engagement | 32.94 | 20.44 |
| Loneliness | 24.41 | 14.44 |
| Social vulnerability | 13.24 | 14.34 |
| Low social network size | 11.47 | 11.08 |
| Living alone | 10.59 | 10.56 |
| Low emotional support | 7.35 | 9.25 |

NOTE. The experts were asked to freely distribute 100 points across the different operationalisations for social contact identified in our umbrella review.

**Supplementary table 6:** Full results for operationalisations of sleep in Delphi round 2, based on the input of 17 dementia experts.

Sleep

| **Operationalisation** | **Allocated points (mean)** | **Standard deviation** |
| --- | --- | --- |
| Sleep-disordered breathing | 19.41 | 22.22 |
| Short sleep duration | 17.50 | 14.50 |
| Insomnia | 16.62 | 11.91 |
| General poor sleep quality | 15.74 | 17.86 |
| Daytime sleepiness | 13.24 | 15.43 |
| Long sleep duration | 10.74 | 11.11 |
| Sleep-related movement disorders | 6.76 | 6.63 |

NOTE. The experts were asked to freely distribute 100 points across the different operationalisations for sleep identified in our umbrella review.
