# Supplementary material for: Umbrella review and Delphi study on modifiable factors for dementia risk reduction
Source: Alzheimers Dement. 2023 Dec 30;20(3):2223–39. doi: 10.1002/alz.13577 (PMC10984497; doi:10.1002/alz.13577)
Supplement: Supplementary file 5 — Supporting Information [file ALZ-20-2223-s011.docx]

Appendix E: Qualtrics Questionnaire Delphi Study Round 1

Start of Block: Introduction block

**Dear professor,**   

Thank you for your interest to participate in our Delphi study. In this two-round study, we want to identify modifiable risk and protective factors for dementia risk reduction in two separate populations and we are keen to capture your perspectives. In the first round of this Delphi study, we ask you to list modifiable risk and protective factors for dementia risk reduction in (1) the general population and conversion to dementia in (2) individuals with subjective cognitive decline (SCD) or mild cognitive impairment (MCI). Your input will be used (1) to update the existing "LIfestyle for BRAin health" (LIBRA) index for the general population and (2) to create a modified version for individuals with SCD/MCI. Further details can be found in the attachment of the invitation email. 

This first Delphi round should take a maximum of 30 minutes to complete. Due to the nature of the information and questions, this survey is best filled out on a laptop or PC for the optimum experience. You are always able to quit and revisit this questionnaire at any given moment. Your link will be active until the 22nd of June. 
 

  
Kind regards,  
Dr. Sebastian Köhler; dr. Kay Deckers; prof. dr. Marjolein de Vugt; dr. Martin van Boxtel; Colin Rosenau, MSc; Lotte Truin, MSc

*NOTE: We asked our experts in the first Delphi round to identify modifiable dementia risk/protective factors for both populations (general population, people with SCD/MCI), because our research group was conducting a parallel systematic literature review focused on identification of modifiable dementia risk/protective factors in individuals with SCD/MCI. To make participation more time-efficient for the Delphi experts, we combined both studies into the same questionnaire for the first Delphi round.*

End of Block: Introduction block

Start of Block: Informed consent

**Informed consent for participation in the “Modifiable risk and protective factors for dementia risk reduction in the general population and individuals with subjective cognitive decline or mild cognitive impairment” study** 
I am informed about the nature and the procedures involved in this study.

I have read the written information about this study and was given the opportunity to ask questions.
I have had time to reflect on my participation in this study.
I understand that participation in this study is completely voluntary.
I understand that I can withdraw my participation in this study at any given time, without giving any reason or any negative consequences.
I give the researchers permission to collect, store and process my responses. No personal data will be collected or asked. 
I give permission to collect my answers through the online platform Qualtrics.
I understand that my responses that are collected in both Delphi rounds, as specified in the information letter, can be anonymously used for scientific research.
I agree to participate in the “Modifiable risk and protective factors for dementia risk reduction in the general population and individuals with subjective cognitive decline or mild cognitive impairment” study.

- Yes, I consent to participate in this study
- No, I do not consent to participate in this study

Skip To: End of Survey If Informed consent for participation in the “Modifiable risk and protective factors for dementia ri... = No, I do not consent to participate in this study

Display This Question:

If Informed consent for participation in the “Modifiable risk and protective factors for dementia ri... = Yes, I consent to participate in this study

First name First name

________________________________________________________________

Display This Question:

If Informed consent for participation in the “Modifiable risk and protective factors for dementia ri... = Yes, I consent to participate in this study

Last name Last name

________________________________________________________________

Date of signature Date of signature

| Month | ▼ January ... |
| --- | --- |
| Day | ▼ January ... |
| Year | ▼ January ... |

| Page Break |  |
| --- | --- |

End of Block: Informed consent

Start of Block: Risk factors

**Modifiable risk and protective factors for the general population**

For the general population, we want to **adapt the existing LIBRA index.** 
  
**First**, we ask to indicate whether the **current twelve LIBRA factors should be included or excluded** in the updated LIBRA index. 
  
**Second**, you can **freely name any other modifiable risk or protective factors** that you believe should be included in the updated LIBRA index and that are not part of the current index.

| Page Break |  |
| --- | --- |

The current LIBRA index was developed based of twelve modifiable dementia risk and protective factors for the general population. Please indicate for every modifiable risk or protective factor whether it **should be included or excluded** in the updated LIBRA index.

|  | Should this modifiable risk or protective factor be included in the updated LIBRA index? | | If you think that this modifiable risk or protective factor should not be included, could you indicate why? |
| --- | --- | --- | --- |
|  | Yes | No | If not, why |
| Cholesterol |  |  |  |
| Chronic kidney disease |  |  |  |
| Coronary heart disease |  |  |  |
| Depression |  |  |  |
| Diabetes |  |  |  |
| High cognitive activity |  |  |  |
| Healthy diet/Mediterranean diet |  |  |  |
| Low/moderate alcohol intake |  |  |  |
| Midlife hypertension |  |  |  |
| Midlife obesity |  |  |  |
| Physical inactivity |  |  |  |
| Smoking |  |  |  |

| Page Break |  |
| --- | --- |

In the text boxes below, we ask you to **freely name new** modifiable dementia risk and protective factors not previously included in the LIBRA index. They should be suitable for risk reduction in the **general population.**

**Risk factors currently included in LIBRA:** cholesterol, chronic kidney disease, coronary heart disease, depression, diabetes, midlife hypertension, midlife obesity, physical inactivity, smoking

**Protective factors currently included in LIBRA:** high cognitive activity, healthy diet/Mediterranean diet, low/moderate alcohol intake
 
Please state as many modifiable risk and protective factors as you think are important. The modifiable risk and protective factors should be given in order of importance, starting with the most important factor and ending with the least important factor. There is no limitation on the number of modifiable risk and protective factors you can give. **NOTE: These are not the modifiable risk and protective factors currently included in the LIBRA index**

- Risk/protective factor 1 (most important) ____________________________________
- Risk/protective factor 2 __________________________________________________
- Risk/protective factor 3 __________________________________________________
- Risk/protective factor 4 __________________________________________________
- Risk/protective factor 5 __________________________________________________
- Risk/protective factor 6 __________________________________________________
- Risk/protective factor 7 __________________________________________________
- Risk/protective factor 8 __________________________________________________
- Risk/protective factor 9 __________________________________________________
- Risk/protective factor 10 __________________________________________________
- Risk/protective factor 11 __________________________________________________
- Risk/protective factor 12 __________________________________________________
- Risk/protective factor 13 __________________________________________________
- Risk/protective factor 14 __________________________________________________
- Risk/protective factor 15 __________________________________________________
- Risk/protective factor 16 __________________________________________________
- Risk/protective factor 17 __________________________________________________
- Risk/protective factor 18 __________________________________________________
- Risk/protective factor 19 __________________________________________________
- Risk/protective factor 20 (least important) _____________________________________

In case you know more than 20 modifiable risk or protective factors, please indicate them in the text field below separated by commas.

________________________________________________________________

________________________________________________________________

________________________________________________________________

________________________________________________________________

________________________________________________________________

| Page Break |  |
| --- | --- |

**Modifiable risk and protective factors for individuals with subjective cognitive decline or mild cognitive impairment**
 
[Please be aware that this part of the Delphi round considers a different population than the previous question]
 
In the text boxes below, we ask you to **freely name** modifiable risk and protective factors that could be used for risk reduction of (conversion to) dementia in individuals with subjective cognitive decline (SCD) or mild cognitive impairment (MCI).

Please state as many modifiable risk and protective factors as you think are important. The risk and protective factors should be given in order of importance, starting with the most important modifiable factor and ending with the least important factor. There is no limitation on the number of modifiable risk and protective factors you can give.

- Risk/protective factor 1 (most important) ______________________________________
- Risk/protective factor 2 __________________________________________________
- Risk/protective factor 3 __________________________________________________
- Risk/protective factor 4 __________________________________________________
- Risk/protective factor 5 __________________________________________________
- Risk/protective factor 6 __________________________________________________
- Risk/protective factor 7 __________________________________________________
- Risk/protective factor 8 __________________________________________________
- Risk/protective factor 9 __________________________________________________
- Risk/protective factor 10 __________________________________________________
- Risk/protective factor 11 __________________________________________________
- Risk/protective factor 12 __________________________________________________
- Risk/protective factor 13 __________________________________________________
- Risk/protective factor 14 __________________________________________________
- Risk/protective factor 15 __________________________________________________
- Risk/protective factor 16 __________________________________________________
- Risk/protective factor 17 __________________________________________________
- Risk/protective factor 18 __________________________________________________
- Risk/protective factor 19 __________________________________________________
- Risk/protective factor 20 __________________________________________________
- Risk/protective factor 21 __________________________________________________
- Risk/protective factor 22 __________________________________________________
- Risk/protective factor 23 __________________________________________________
- Risk/protective factor 24 __________________________________________________
- Risk/protective factor 25 __________________________________________________
- Risk/protective factor 26 __________________________________________________
- Risk/protective factor 27 __________________________________________________
- Risk/protective factor 28 __________________________________________________
- Risk/protective factor 29 __________________________________________________
- Risk/protective factor 30 (least important) _____________________________________

In case you know more than 30 modifiable risk or protective factors, please indicate them in the text field below separated by commas.

________________________________________________________________

________________________________________________________________

________________________________________________________________

________________________________________________________________

________________________________________________________________

| Page Break |  |
| --- | --- |

End of Block: Risk factors

Start of Block: End of Survey

***Co-authorship*** 
We very much appreciate your willingness to share your knowledge with us. This is indispensable for our research. Therefore, we would like to invite you to become co-author of the paper(s) resulting from this Delphi study. This entails that you will be asked at a later stage to provide your feedback on draft(s) of the manuscript(s).

- Yes, I wish to contribute to resulting paper(s) as a co-author
- No, I do not wish to contribute to resulting paper(s) as a co-author

***Future updates***
Would you like to receive a notification when a paper is published based on this Delphi Study?

- Yes, I wish to receive a notification when a paper is published based on this Delphi Study
- No, I do not wish to receive a notification when a paper is published based on this Delphi Study

End of Block: End of Survey
