# Supplementary material for: Umbrella review and Delphi study on modifiable factors for dementia risk reduction
Source: Alzheimers Dement. 2023 Dec 30;20(3):2223–39. doi: 10.1002/alz.13577 (PMC10984497; doi:10.1002/alz.13577)
Supplement: Supplementary file 7 — Supporting Information [file ALZ-20-2223-s006.docx]

Appendix G: Qualtrics Questionnaire Delphi Study Round 2

Start of Block: Introduction block

**Dear Professor,**

Thank you for participating in the first round of our Delphi consensus study and for your willingness to participate in this second round. In this second round, we will present our findings from (1) the first Delphi study round and; (2) our umbrella review of systematic literature reviews on individual dementia risk and protective factors. We will provide you with an updated list of the most important modifiable dementia risk and protective factors identified for the **general population** and ask you to critically assess the results. Subsequently, this information will be used to update the current LIBRA index. 
This second Delphi study round should take you **10 minutes or less** to complete. Ideally, this questionnaire is filled out on a laptop or PC for the best experience. You can quit and revisit this questionnaire at any given moment. Your personal link to access the questionnaire will remain active until the 23rd of December 2022.

Many thanks again for taking the time to participate in our Delphi consensus study.

Kind regards, 
Dr. Sebastian Köhler; dr. Kay Deckers; prof. dr. Marjolein de Vugt; dr. Martin van Boxtel; dr. Irene Heger; Colin Rosenau, MSc; Lotte Truin, MSc

End of Block: Introduction block

Start of Block: Information block

**New modifiable risk and protective factors for the general population**
On the next two pages, we will first show you the most important new modifiable risk and protective factors for dementia risk reduction in the general population according to the results of the first Delphi round and our umbrella review. Subsequently, we ask you to distribute points among these factors to come to a final ordered list. We also kindly ask you to answer some additional questions on the operationalization of some specific risk factors.

End of Block: Information block

Start of Block: First Delphi Round

**Results first Delphi study round**
Initially, 60 different answers were provided, however, many of them were thematically related and, therefore, combined into 27 factors **not previously included in the LIBRA** index. The answers were weighted on an exponential scale. Table 1 shows the list of the most important new modifiable risk and protective factors (not previously included in the LIBRA index), according to the Delphi experts (N=18). More extensive methodological information can be found in this [PDF file](https://maastrichtuniversity.eu.qualtrics.com/CP/File.php?F=F_5cKZiS7mq4i4RCK). The full results can be found in this [PDF file](https://maastrichtuniversity.eu.qualtrics.com/CP/File.php?F=F_3fIzh57aIO4jlem).

**Table 1: The most important new modifiable risk and protective factors (not previously included in the LIBRA index) according to the Delphi experts.**

| **Modfiable factor** | **N** | **Ranks** | **Rank score** | **Final rank** |
| --- | --- | --- | --- | --- |
| Hearing impairment | 11 | 1,1,1,2,2,3,3,3,4,4,8 | 761 | 1 |
| Social isolation/loneliness | 13 | 1,2,2,2,3,3,3,3,3,4,4,5,7 | 723 | 2 |
| Traumatic brain injury* | 11 | 1,1,2,2,3,3,3,4,4,5,7 | 704 | 3 |
| Education* | 8 | 1,1,2,2,2,3,4,5 | 592 | 4 |
| Sleep | 8 | 1,1,1,2,2,4,5,6 | 572 | 5 |
| Stroke/cerebrovascular disease* | 5 | 1,1,1,5,8 | 345 | 6 |
| Air pollution* | 7 | 1,3,4,5,5,6,7 | 326 | 7 |
| Psychological stress | 3 | 2,3,8 | 154 | 8 |
| Visual impairment | 4 | 3,4,5,12 | 149 | 9 |
| Lifecourse inequalities | 1 | 1 | 100 | 10 |
| Atrial fibrillation | 3 | 4,6,8 | 83 | 11 |

*In alignment with the development of the original LIBRA index, we do not consider these factors to be readily modifiable at an individual level (which does not mean that they are not modifiable at the population level). Because the LIBRA index is a tool to identify an individual’s room for improvement in lifestyle, we omitted these factors from further analysis in this second Delphi study round.

End of Block: First Delphi Round

Start of Block: Systematic searches

**Results umbrella review**
In parallel with the first round of this Delphi study, we conducted an umbrella review to identify the most important new modifiable risk and protective factors for risk reduction of dementia according to the current literature. Our search returned 6,540 systematic reviews and meta-analyses of which 463 (7.08%) were included for full-text screening based on their title and abstract. To date, 125 systematic reviews and meta-analyses have been included in our review. We determined how frequently each modifiable risk or protective factor was discussed and how consistent its effect was with dementia/cognitive impairment/cognitive decline. Table 2 shows the most important newly identified modifiable risk and protective factors (not previously included in the LIBRA index) according to the literature.  More extensive methodological information can be found in this [PDF file](https://maastrichtuniversity.eu.qualtrics.com/CP/File.php?F=F_1ZjGgi5ruwrNJ9I).

**Table 2: The most important new modifiable risk and protective factors (not previously included in the LIBRA index) according to the literature.** These were ordered based on their frequency and their consistency of association in the literature.

| **Modifiable factor** | **N** | **Increased risk** | **Neutral risk** | **Decreased risk** | **Consistency of effect** |
| --- | --- | --- | --- | --- | --- |
| Low social engagement/participation in activities | 28 | 14 | 14 | 0 | 50% |
| Hearing impairment | 18 | 16 | 2 | 0 | 89% |
| Anxiety | 14 | 10 | 4 | 0 | 71% |
| Visual impairment | 14 | 10 | 4 | 0 | 71% |
| Atrial fibrillation | 12 | 9 | 3 | 0 | 75% |
| Tooth loss | 10 | 7 | 3 | 0 | 70% |
| Olfactory impairment | 9 | 9 | 0 | 0 | 100% |
| Loneliness | 9 | 6 | 3 | 0 | 67% |
| Insomnia | 8 | 4 | 4 | 0 | 50% |
| Daytime sleepiness | 4 | 4 | 0 | 0 | 100% |
| High psychological stress | 4 | 3 | 1 | 0 | 75% |
| Social vulnerability | 3 | 3 | 0 | 0 | 100% |

End of Block: Systematic searches

Start of Block: Point allocation

**Point allocation (general population)**

This second round aims to identify and prioritize those factors that should be added to the existing LIBRA index. Therefore, we ask you to distribute 100 points to the factors in the list below, which was constructed based on the combined results of the first Delphi round and umbrella review. Whenever you believe that a particular factor should not be included, you can assign 0 points to that factor. More points should be given to those factors that you think are important for dementia risk reduction.
Liberally distribute 100 points to the factors (**in alphabetical order**) in the list below

Anxiety : _______

Atrial fibrillation : _______

Hearing impairment : _______

Lifecourse inequalities : _______

Olfactory impairment : _______

Psychological stress : _______

Sleep : _______

Social contact : _______

Tooth loss : _______

Visual impairment : _______

Total : ________

End of Block: Point allocation

Start of Block: Sleep & Social Contact

**Social contact & Sleep**

From both the first round of this Delphi study and our umbrella review of the literature, it became apparent that both ***sleep*** and ***social contact*** would be appropriate targets for dementia risk reduction. However, these terms tend to be rather broadly defined and operationalized differently in various studies. In the two questions below, we provide you with the different operationalizations that we encountered. For both factors, we ask you to distribute 100 points to these operationalizations in order to determine which one would be the most important to target for dementia risk reduction. For each operationalization, we indicated the frequency (N) encountered in the literature and consistency (Con) with increased risk for dementia/cognitive impairment/cognitive decline. All operationalizations encountered with a frequency ≤ 2 were omitted.

Liberally distribute 100 points to your preferred operationalizations of **social contact** in the list below

Low social engagement/participation (N = 28, Con = 50%) : _______

Loneliness (N = 9, Con = 67%) : _______

Low social network size (N = 11, Con = 9%) : _______

Living alone (N = 10, Con = 20%) : _______

Low emotional support (N = 5, Con = 0%) : _______

Social vulnerability (N = 3, Con = 100%) : _______

Total : ________

Additional comments on your distribution for **social contact** can be provided in the box below

________________________________________________________________

________________________________________________________________

________________________________________________________________

________________________________________________________________

________________________________________________________________

Liberally distribute 100 points to your preferred operationalizations of **sleep** in the list below

Short sleep duration (N = 18, Con = 33%) : _______

Long sleep duration (N = 17, Con = 47%) : _______

Insomnia (N = 8, Con = 50%) : _______

Sleep-disordered breathing (N = 7, Con = 29%) : _______

General poor sleep quality (N = 5, Con = 40%) : _______

Daytime sleepiness (N = 4, Con = 100%) : _______

Sleep-related movement disorder (N = 3, Con = 33%) : _______

Total : ________

Additional comments on your distribution for **sleep** can be provided in the box below

________________________________________________________________

________________________________________________________________

________________________________________________________________

________________________________________________________________

________________________________________________________________

End of Block: Sleep & Social Contact
